# Paper and Cardboard Packaging: From Cellulosic Substrates to Functional and Hybrid Architectures

**DOI:** 10.3390/ma19132801

**Published:** 2026-07-01

**Authors:** Leonardo Pagnotta

**Affiliations:** Department of Mechanical, Energy and Management Engineering, University of Calabria, Arcavacata, 87036 Rende, Italy; leonardo.pagnotta@unical.it

**Keywords:** paper-based packaging, functional coatings, multilayer architectures, hybrid packaging systems, barrier properties, converting compatibility, recyclability, compostability, food-contact materials, sustainable packaging

## Abstract

**Highlights:**

- Interprets paper-based packaging as an engineered architecture in which substrate, coating, interface, and multilayer design jointly determine performance.
- Develops a classification framework linking coating composition, microstructural organisation, and architectural complexity to barrier mechanisms and functional behaviour.
- Analyses the relationships between barrier performance, mechanical integrity, converting compatibility, food-contact safety, and end-of-life management.
- Identifies the key trade-offs between barrier efficiency, recyclability, and circularity in coated and multilayer paper packaging systems.
- Highlights design-for-recycling and controlled delamination as promising strategies for next-generation sustainable paper-based packaging.

**Abstract:**

Paper and cardboard are widely used in packaging due to their renewable origin, low density, printability, and established recycling infrastructures. However, monolithic cellulosic substrates are intrinsically limited by porosity and moisture sensitivity, resulting in inadequate barrier performance for demanding applications. Consequently, paper-based packaging has evolved toward functionalised systems based on coatings, multilayers, and hybrid architectures. This review adopts a system-level approach based on a structured and criteria-driven analysis of the scientific and technical literature to examine the transition from base cellulosic substrates to advanced paper-based packaging structures. The study integrates material composition, layer architecture, and interfacial phenomena, and develops a classification and interpretation framework that systematically links structural design variables to key performance domains, including barrier behaviour, mechanical integrity, converting compatibility, food-contact safety, and end-of-life management. Particular emphasis is placed on the role of functional layers as critical design variables governing both performance enhancement and circularity constraints. By systematically correlating structure, mechanisms, and functional outcomes, the analysis highlights the central trade-offs between barrier efficiency and recyclability and identifies design-for-recycling and controlled delamination as key strategies for the development of next-generation sustainable paper-based packaging.

## 1. Introduction

Paper and cardboard remain among the most widely adopted materials in packaging systems, particularly in food applications, beverage, pharmaceutical, cosmetic, and consumer goods sectors [1,2,3,4,5]. Their widespread use derives from a combination of intrinsic and process-related properties, including renewable origin, relatively low density, favourable stiffness-to-weight ratio, and high printability, together with compatibility with established collection and recycling infrastructures. In addition, fiber-based substrates exhibit high adaptability to industrial converting operations, such as folding, forming, embossing, and surface functionalisation, which enables their integration into a broad range of packaging formats [3,5].

In response to increasing environmental concerns and the need to reduce dependence on fossil-based plastics, paper-based packaging has gained renewed attention as a viable component of sustainable packaging strategies aligned with circular economy principles [6,7].

However, the porous and hydrophilic nature of cellulose inherently limits its barrier performance against water, water vapour, gases, light, and contaminants [3,8,9]. Consequently, monolithic paper substrates are generally inadequate for applications requiring extended shelf life or grease resistance when compared to glass, metals, or high-performance polymers [10,11,12,13]. To overcome these limitations, paper packaging has evolved toward functionally engineered, architecturally complex systems. These include surface coatings, bio-based/biodegradable polymer layers, nanocomposite reinforcements, and multilayer structures [6,7,14].

Yet, these functionalisation strategies introduce critical trade-offs. While multilayer and coated structures significantly improve barrier and mechanical performance, they may simultaneously hinder recyclability and complicate end-of-life management, as well as introduce challenges related to food-contact safety and chemical migration [6,14].

Current literature on paper-based packaging is substantial but largely fragmented, typically focused on isolated material classes, specific coating formulations, or single performance targets. Consequently, existing reviews generally treat paper merely as a passive substrate to be functionalised, separate from the converting operations or regulatory constraints that dictate its real-world viability. This fragmentation hinders a unified understanding of how functional gains affect processing and circularity targets.

To address this limitation, this review shifts the paradigm from a material-centric analysis to an architecture-driven, system-level framework. Here, paper-based packaging is interpreted as an engineered material system where substrate morphology, coating chemistry, interfacial interactions, and multilayer configurations jointly dictate performance. The innovation of this approach lies in explicitly mapping the structure–architecture–performance correlations, directly linking design variables to the intersecting demands of barrier behavior, converting compatibility, food safety, and end-of-life pathways. By integrating these dimensions into a unified scheme, this work provides a comparative, design-oriented perspective to navigate performance–circularity trade-offs and support the rational development of next-generation paper packaging.

**Figure 1:**
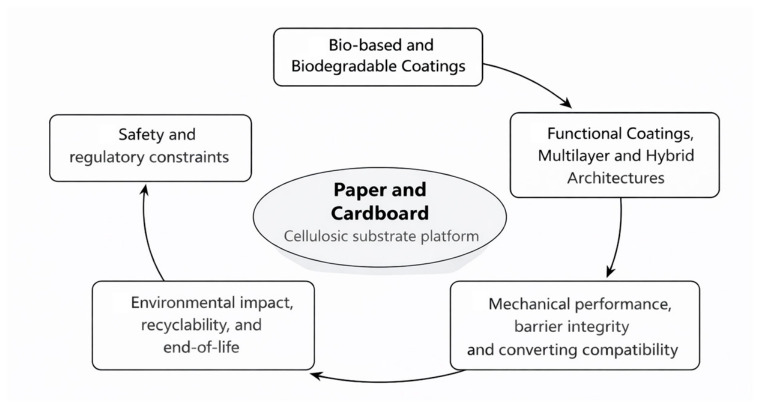
**System-level roadmap for paper-based packaging architectures.** The diagram illustrates the progressive transition from simple cellulosic substrates to functionalised packaging systems. It highlights the successive design levels -including bio-based coatings, hybrid multilayers, mechanical converting, environmental end-of-life pathways, and safety compliance- reflecting the integrated logic adopted throughout this review.

The organisation of the review follows the roadmap illustrated in Figure 1, guiding the analysis of these interdependencies across the subsequent sections.

### 1.1. Evolution of Paper and Cardboard in Packaging

Paper and cardboard have played a foundational role in the development of packaging systems, although the concept of packaging itself predates industrial materials by several millennia. Early societies relied on naturally available resources such as leaves, animal skins, woven fibers, wood, ceramics, and glass to contain and transport goods. Among the earliest documented uses of paper for packaging can be traced to ancient China, where treated mulberry bark sheets were used for wrapping food, marking an early transition from natural containers to engineered fibrous materials [10,15]. Papermaking knowledge gradually spread westward, reaching Europe during the late Middle Ages and ultimately enabling industrial-scale production.

The emergence of commercial paper packaging during the nineteenth century represented a decisive technological transition. Paper bags began to be manufactured industrially in England in the mid-1800s, followed by the development of paperboard cartons and corrugated fiberboard. Corrugated board, introduced in the mid-nineteenth century and widely adopted for transport packaging by the early twentieth century, offered a favourable strength-to-weight ratio, low cost, and ease of processing, progressively replacing wooden crates in distribution systems [15,16].

Between approximately 1910 and the mid-twentieth century, paper-based packaging underwent substantial functional and industrial consolidation. Corrugated cartons became the dominant solution for transport applications following regulatory acceptance by rail systems, while folding cartons expanded rapidly in consumer markets such as cereals and dry foods. In parallel, moulded fiber technologies emerged, with early patents dating to the early twentieth century and applications including egg cartons, cushioning elements, and protective packaging for fragile goods. These developments illustrate the transition from simple wrapping materials to engineered fiber-based systems designed for logistics efficiency, product protection, and industrial scalability [15,17,18].

As industrial food production expanded during the twentieth century, the intrinsic limitations of monolithic paper substrates became increasingly evident. The porous and hydrophilic nature of cellulose fibers results in high permeability to gases, water vapour, and grease, together with sensitivity to humidity and mechanical degradation under wet conditions [2,3]. Consequently, untreated paper remained largely confined to dry goods and secondary packaging applications. Progressive material differentiation therefore emerged through the kraft pulping, parchmentisation, waxing, coating, and lamination processes widely reported in the literature aimed at improving strength, surface properties, and limited barrier performance.

A major shift occurred in the second half of the twentieth century with the introduction of polymer coatings, aluminium foils, and multilayer laminates. Paper-based packaging evolved from single-material fibrous substrates into hybrid architectures combining cellulose fibers with polymers, metals, or functional additives. These configurations enabled extended shelf life, improved mechanical reliability, and compatibility with industrial filling and sealing processes, although they also introduced challenges related to recyclability, material compatibility, and end-of-life management [16].

More recently, sustainability pressures, circular-economy policies, and plastic-reduction strategies have renewed interest in fiber-based packaging systems. Current research focuses on bio-based coatings, nanocellulose reinforcement, recyclable barrier layers, and functional hybrid architectures capable of combining performance with environmental compatibility [7,14,19].

This historical progression—from early fibrous wrapping materials to engineered multilayer systems—demonstrates that the evolution of paper packaging has not been driven solely by material development but by the need to integrate multiple and often competing functional requirements within a single system.

**Figure 2:**
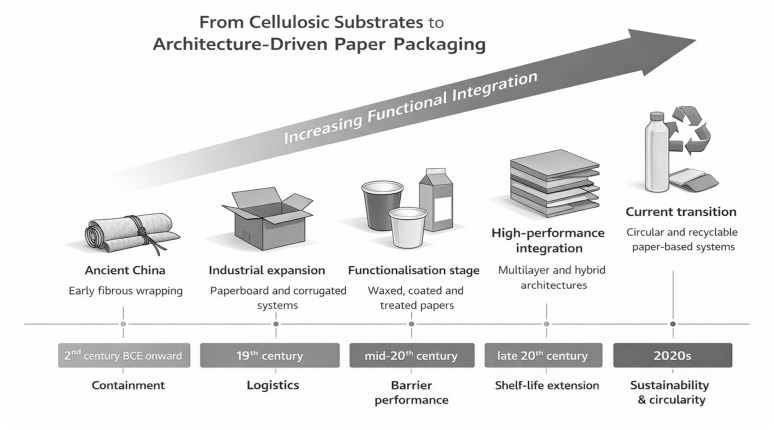
**Historical evolution of paper and cardboard packaging materials.** Schematic representation of the technological transition from early fibrous wrapping materials to industrial paperboard, functionalised substrates, and modern multilayer hybrid architectures. The timeline highlights how the primary driver of packaging design has shifted over time from basic containment to advanced barrier performance and current sustainability requirements.

Modern paper and cardboard packaging can therefore be interpreted as engineered material architectures in which substrate morphology, functional layers, processing requirements, and end-of-life constraints interact to determine overall performance. The main stages of this evolution are schematically illustrated in Figure 2.

### 1.2. Scope and Methodological Approach

The present review is based on a structured and criteria-driven analysis of the scientific and technical literature on paper-based packaging systems, with particular emphasis on coating technologies, multilayer architectures, and their associated functional performance. The analysis is conducted within the system-oriented perspective introduced in Section 1, in which material composition, structural design, and functional behaviour are interpreted as interconnected dimensions.

The literature survey was conducted using major scientific databases, including Scopus, Web of Science, and Google Scholar, complemented by targeted searches within leading publishers’ platforms (Elsevier, Springer, Wiley, and MDPI). The search strategy was defined through combinations of keywords such as “paper-based packaging”, “barrier coatings”, “bio-based coatings”, “multilayer paper packaging”, “nanocellulose coatings”, “functional packaging”, and “food contact materials”. Additional references were identified through backward and forward citation tracking of key review articles and primary studies.

The selection of the literature followed explicit inclusion criteria. Studies were considered when they provided relevant information on material composition, coating or multilayer structure, processing methods, barrier properties (e.g., water vapour transmission rate ‘WVTR’ and oxygen transmission rate ‘OTR’), mechanical behaviour, converting compatibility, or end-of-life aspects. Preference was given to peer-reviewed journal articles, while books, book chapters, technical reports, conference proceedings, and regulatory or guidance documents were included when they contributed essential complementary information, particularly in relation to industrial practices, standardisation, and safety assessment.

Works not directly related to paper and paperboard substrates, or lacking sufficient technical detail to support structure–architecture–performance correlations, were excluded. Similarly, studies focused exclusively on non-packaging applications (e.g., biomedical or electronic uses of coatings) were considered only when their findings were directly transferable to packaging systems. The resulting body of literature reflects a deliberate balance between recent developments and foundational contributions, with priority given to more recent studies to capture current advances in coating technologies, multilayer design, migration phenomena, and sustainable strategies.

**Figure 3:**
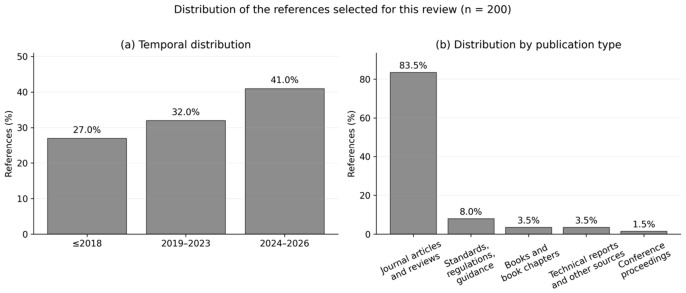
**Distribution of the 202 references examined in this review**: (**a**) temporal distribution, grouped into three intervals; (**b**) distribution by publication type, reported in descending order. The figure documents the structure of the reference set adopted in this review and is not intended as a bibliometric analysis.

The composition of the selected literature is summarised in Figure 3.

Generative artificial intelligence (AI) tools were used exclusively to support language editing, text refinement, and manuscript preparation. The literature search and selection, critical evaluation of the sources, interpretation of the reviewed studies, development of the classification framework, preparation of tables and figures, and all scientific conclusions were performed and validated by the author.

Given the heterogeneity of experimental conditions across the literature—particularly in terms of substrate type, coating formulation, processing parameters, and testing protocols—direct quantitative comparison is not always feasible. For this reason, the analysis is primarily conducted on a qualitative or semi-quantitative basis, using consistent descriptors to identify relative trends in barrier performance, mechanical behaviour, converting compatibility, safety, and end-of-life implications.

Within this methodological framework, coating and multilayer systems are systematically classified by material class, microstructural organisation, and architectural complexity. These configurations are analysed in relation to their dominant barrier mechanisms, functional roles, and limitations, enabling a consistent interpretation of structure–architecture–performance relationships. This integrated approach provides the analytical basis for the comparative and radar-based representations developed in the following sections.

## 2. Monolayer Coating Systems for Paper-Based Packaging: Materials, Mechanisms and Performance

Paper and cardboard are intrinsically porous, hydrophilic, and structurally heterogeneous materials whose barrier performance is inherently limited with respect to water vapour, grease, and gas transport. These uncoated cellulosic substrates, including primary paper grades such as kraft papers and paperboards, provide structural support and mechanical strength but offer only limited barrier functionality [2,5,8,20].

In these systems, the absence of continuous functional layers leaves the fibrous network fully exposed, and transport phenomena are governed by the interconnected pore structure and capillary pathways formed between fibers. As a result, uncoated substrates exhibit high permeability to gases and water vapour, poor resistance to liquid penetration, and negligible sealing capability, which significantly restricts their direct use in high-performance packaging applications [2,8].

The hydrophilic nature of cellulose, resulting from the abundance of hydroxyl groups, promotes moisture sorption and swelling. These phenomena cause dimensional instability and deterioration of mechanical properties under humid conditions [5,8]. In addition, the limited surface smoothness and chemical compatibility of uncoated papers restrict their use in advanced converting operations such as high-resolution printing, lamination, and heat sealing (Chamberlain, 2013; Kirwan, 2011) [20,21]. In addition to uncoated substrates, paper may also undergo internal modification or impregnation treatments aimed at improving moisture resistance or mechanical stability. However, these approaches remain relatively limited in packaging applications and do not introduce discrete functional layers. For this reason, they are not treated as a separate category in the present work.

Before the widespread development of modern bio-based coating technologies, water- and grease-resistance in paper-based packaging was commonly achieved through polymer extrusion coatings and fluorinated surface treatments. Polyethylene (PE) coatings represent one of the most established industrial solutions, forming a continuous barrier layer that effectively improves moisture resistance. However, these multilayer structures significantly hinder recyclability, as the separation of the polymer layer from the fibrous substrate requires dedicated processes and additional costs. In parallel, fluorinated treatments have been widely used to impart water and oil repellence through surface energy reduction. Unlike polymer coatings, these systems act primarily through surface energy modification. Despite their effectiveness, increasing concerns related to environmental persistence, bioaccumulation, and potential health risks have led to progressive restrictions on their use. As a result, current research is increasingly focused on per- and polyfluoroalkyl substances (PFAS)-free and more recyclable alternatives based on bio-based coatings, nanostructured systems, and multilayer architectures capable of combining barrier performance with improved environmental compatibility [22,23]. Coatings therefore represent a key strategy for transforming cellulosic substrates into packaging materials suitable for diverse applications. Food contact remains among the most demanding, due to strict barrier and safety requirements. Although food packaging is the main driver behind the development of barrier coatings, similar functional demands are increasingly relevant in other sectors, including consumer goods and technical packaging. In this context, coatings act as engineered interfacial layers capable of modifying transport phenomena, surface energy, and mechanical performance [9,14,24].

From a materials science perspective, their effectiveness is governed by the formation of continuous films, the reduction in accessible porosity, and the control of diffusion pathways for gases and vapors. These effects depend on both coating chemistry and structural organization [9,24]. In more advanced systems, barrier performance is further enhanced through microstructural control, for example, by generating tortuous diffusion pathways through fillers or nanostructured phases [25,26,27].

### 2.1. Classification Framework and Barrier Mechanisms

Previous reviews have traditionally classified coating systems according to the chemical nature of the materials employed, distinguishing among polysaccharides, proteins, lipids, and bio-based polyesters, often combined in composite or multilayer configurations [10,19]. While this approach remains useful, it does not fully account for the influence of structural design and processing route on coating performance. Since the literature does not always distinguish consistently between coatings, films, and laminated structures, this review adopts a functional-layer criterion. Accordingly, monolayer systems are defined as configurations with one functional layer applied to the paper substrate, whereas multilayer systems include two or more distinct functional layers. In light of these considerations, the classification adopted in this work extends conventional material-based approaches by incorporating both compositional and structural criteria. Specifically, coating systems are organised into four main material-driven categories—polysaccharide-based systems, protein- and lipid-based systems, synthetic and bio-polymer coatings, and hybrid or nanostructured systems—complemented by a fifth category that accounts for multilayer and extrusion-coated architectures. This framework enables a more comprehensive interpretation of recent developments, where performance is increasingly governed by the synergistic combination of material selection, structural design, and processing strategy [7,9,14].

It should be noted that coating performance is governed not only by material composition and structural organisation, but also by the deposition technology used to apply the functional layer. Coating methods such as rod coating, bar coating, blade coating, spray coating, curtain coating, dip coating, and extrusion coating influence coat weight, coating continuity, substrate penetration, defect formation, surface coverage, and interfacial interactions with the fibrous network. Consequently, differences in barrier, mechanical, and converting performance reported in the literature cannot always be attributed solely to coating chemistry but may also reflect variations in processing route and deposition conditions. For this reason, coating technology should be considered an additional design variable contributing to the final performance of paper-based packaging systems.

Beyond barrier enhancement, coating systems also influence the physical and mechanical behaviour of paper-based substrates. Depending on coating composition, coat weight, and deposition conditions, improvements have been reported in tensile strength, stiffness, burst resistance, compressive performance, surface strength, and dimensional stability. These effects are generally associated with pore filling, fiber bonding enhancement, surface densification, and stress redistribution within the fibrous network. However, the magnitude of these improvements varies considerably among coating classes and processing routes, and may be accompanied by reduced flexibility, increased brittleness, or moisture sensitivity. Consequently, coating design should be considered not only as a barrier-engineering strategy but also as a means of tailoring the mechanical and converting performance of paper-based packaging systems.

Table 1 summarises the classification of coating systems according to the main material and structural categories analysed in the following sections and is intended as a reference framework to guide the subsequent discussion.

**Figure 4:**
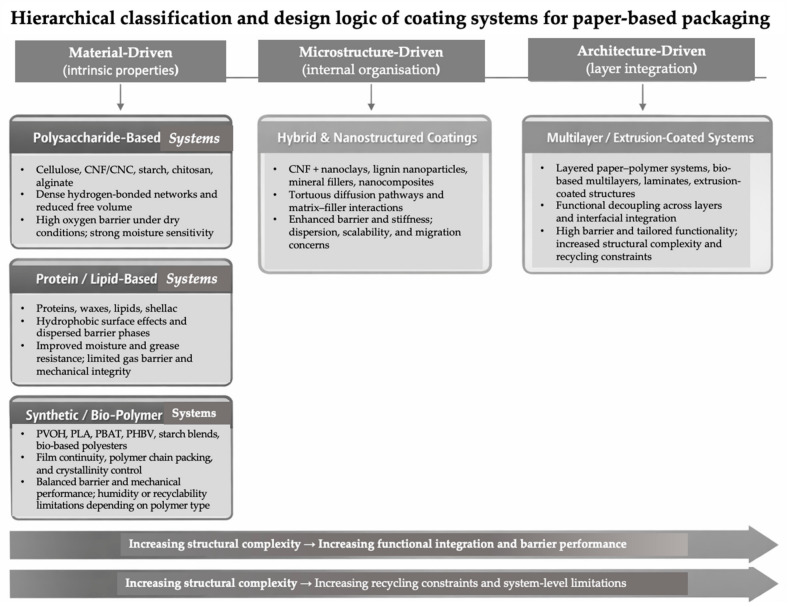
**Hierarchical classification of coating systems for paper-based packaging.** Coating systems are grouped according to their dominant design criterion: material-driven, microstructure-driven, or architecture-driven. The figure relates each group to its material basis, main barrier mechanism, and functional role, showing the transition from composition-controlled coatings to more complex nanostructured and multilayer systems.

This classification is further rationalised in Figure 4, which illustrates the hierarchical organisation of coating systems according to their dominant design criteria. In particular, the diagram highlights the transition from material-driven approaches, governed by intrinsic material properties, to microstructure- and architecture-driven systems, where barrier behaviour increasingly depends on nanostructural organisation and multilayer integration.

It is important to note that certain functionalities—such as antimicrobial activity, controlled release of active agents, or migration control—are not treated here as independent categories but rather as transversal design features that can be integrated within different material systems. These aspects are therefore discussed within the relevant subsections, in order to avoid overlaps and to maintain a clear distinction between material classification and functional performance.

**Figure 5:**
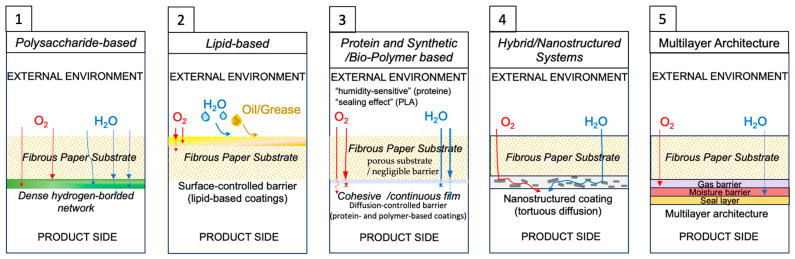
**Schematic representation of the dominant barrier mechanisms in coated paper-based packaging systems.** Panel (1) represents dense hydrogen-bonded networks, typically associated with cellulose- or nanocellulose-based polysaccharide coatings, where barrier performance is governed by reduced free volume and restricted molecular diffusion. Panel (2) illustrates surface-controlled barrier behaviour, characteristic of lipid-based coatings, where reduced wettability and suppression of capillary penetration primarily limit liquid transport. Panel (3) represents cohesive or continuous film-forming coatings, including protein-based and synthetic/bio-polymer-based systems, in which barrier performance is mainly governed by diffusion through a relatively continuous layer and by substrate sealing effects. Panel (4) shows hybrid and nanostructured coatings generating tortuous diffusion pathways through the incorporation of dispersed filler phases. Panel (5) represents multilayer architectures, where distinct functional layers provide complementary barrier and structural functions. The figure highlights the transition from single-component barrier mechanisms to more complex microstructure- and architecture-controlled designs.

Figure 5 summarises the dominant barrier mechanisms associated with the coating categories discussed.

### 2.2. Polysaccharide-Based Coatings

Polysaccharide-based coatings are among the most extensively investigated classes of bio-based systems for paper functionalisation, owing to their abundance, renewability, and intrinsic film-forming ability [28,29,30,31,32,33,34]. Materials such as cellulose and its derivatives, starch, chitosan, and alginate are widely employed to generate continuous coatings capable of significantly improving gas barrier performance, particularly against oxygen and non-polar molecules [9,24].

As schematically illustrated in Figure 5, these systems typically improve barrier properties by forming compact coating layers on the paper surface, where dense polymer networks limit molecular diffusion. In this context, the first panel represents the dense-network mechanism typically associated with cellulose- or nanocellulose-based coatings, whereas other polysaccharide systems may rely more strongly on film continuity, substrate sealing, or formulation-dependent effects [35]. Their effectiveness is strongly dependent on the quality of film formation and on the interaction between the coating and the fibrous substrate [36,37].

**Figure 6:**
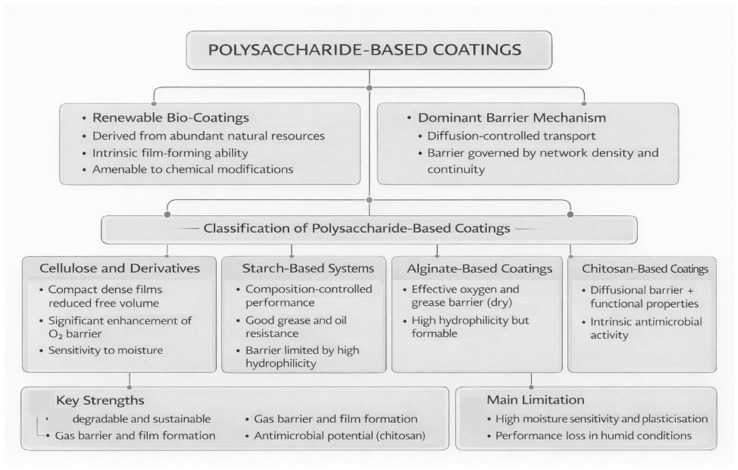
**Schematic classification of polysaccharide-based coating systems for paper and paperboard, highlighting their structural organisation and dominant barrier mechanisms.** The systems are grouped into cellulose-derived coatings, starch-based systems, alginate-based coatings, and chitosan-based systems. The diagram emphasises the diffusion-controlled transport mechanism associated with dense polymer networks, as well as the role of coating continuity, formulation, and interfacial interactions in determining barrier performance on fibrous substrates. The intrinsic moisture sensitivity common to hydrophilic polysaccharides is also highlighted as a key limiting factor.

The main polysaccharide-based coating systems discussed in this section are schematically classified in Figure 6, according to their material origin, structural organisation, dominant barrier mechanism, and main limitations.

#### 2.2.1. Cellulose-Based Coating Systems

Among polysaccharide-based systems, cellulose-derived coatings have been extensively investigated due to their ability to form dense and continuous structures associated with low gas permeability. Fundamental studies on nanocellulose films have shown that, when well-formed, these materials can generate compact networks with reduced free volume, leading to significant improvements in oxygen barrier performance. In particular, Herrera et al. [38] demonstrated that plasticised and cross-linked nanocellulose coatings can significantly improve both oxygen and water vapour barrier performance when dense and homogeneous structures are achieved.

When moving from model systems to real paper substrates, however, the effectiveness of cellulose-based coatings becomes strongly dependent on coating continuity, formulation, and deposition conditions. In this regard, Sabo et al. [39] showed that cellulose-based coatings, including cellulose nanocrystals (CNC), tempo-oxidized cellulose nanofibers (TOCN), and carboxymethyl cellulose (CMC), can significantly improve grease and air barrier performance when the coating process ensures effective film formation and adequate surface coverage. This point is particularly important because it highlights that the barrier response of cellulose coatings is not controlled only by the intrinsic properties of nanocellulose, but also by the extent to which the porous paper surface is actually sealed.

This behaviour is consistent with other experimental studies on coated paper substrates. Afra et al. (2016) [40] showed that cellulose nanofibril (CNF) coatings reduce surface porosity and improve air resistance, tensile strength, and surface properties, with performance strongly influenced by coating concentration and number of layers. Similarly, Jin et al. (2021) [41] reported that nanofibrillated cellulose coatings lead to reduced water absorption, improved air resistance, and enhanced mechanical properties, with optimal performance observed at intermediate CNF loadings that provide more uniform surface coverage.

The role of formulation is further highlighted in systems incorporating additives. Mousavi et al. [42] demonstrated that the addition of carboxymethyl cellulose (CMC) significantly improves coating uniformity by reducing nanofiber flocculation, enabling more homogeneous film formation and, consequently, improved barrier and structural properties. These findings highlight the importance of formulation and dispersion quality in determining coating performance. Despite these improvements, limitations remain evident when cellulose coatings are applied to paperboard systems. Lavoine et al. [43] showed that microfibrillated cellulose (MFC) coatings can enhance mechanical properties such as bending and compressive stiffness, but their contribution to barrier performance is limited when full surface coverage is not achieved or when coat weight is insufficient. More generally, the intrinsic hydrophilicity of cellulose remains a critical drawback, since moisture uptake induces swelling and plasticisation, thereby deteriorating barrier performance under high-relative-humidity conditions [9].

The structure–property relationships of cellulose-based mono-coatings are summarised in Table 2, where both intrinsic film behaviour and coating performance on paper substrates are compared. Since the reference condition is not uniform across the selected studies, it is specified directly within the composition description for each system.

#### 2.2.2. Starch-Based Coatings

Starch-based coatings are among the most widely investigated polysaccharide systems for paper functionalisation because of their availability, low cost, and film-forming ability [24]. However, their barrier performance remains strongly limited by hydrophilicity, which restricts moisture resistance despite the formation of continuous films. Recent reviews identify poor moisture resistance, limited thermal stability, and insufficient mechanical performance as the main constraints to the standalone use of starch in demanding packaging applications [44].

When applied to paper substrates, starch coatings exhibit behaviour that is strongly dependent on formulation and network structure. In this regard, Christophliemk et al. [45] systematically investigated starch –poly(vinyl alcohol) (PVOH) coating systems directly on paper and showed that starch-rich coatings exhibit very high WVTR values (≈247 g·m^−2^·day^−1^), confirming that starch alone remains a weak moisture barrier. However, the introduction of PVOH drastically reduces WVTR at intermediate compositions (~2–10 g·m^−2^·day^−1^), while barrier performance deteriorates again at high starch contents, indicating the presence of a structural breakpoint governed by phase organisation and surface composition.

This formulation-dependent behaviour is further confirmed by studies on more complex coating systems. Chi et al. [46] demonstrated that starch-based polyelectrolyte complex (PEC) coatings can form dense, homogeneous and defect-free layers on paperboard, leading to improved mechanical properties together with enhanced resistance to water vapour, grease, and oil. Similarly, Xia et al. [47] reported that modified starch coatings incorporating lignin derivatives and cellulose nanofibrils generate hybrid networks with improved air barrier behaviour, surface hydrophobicity, and mechanical performance. Chemical modification can significantly improve starch-coating performance. For example, Zhang et al. [48] reported that cationic-acetylated glutinous rice starch combined with chitosan formed a smooth and dense coating layer that completely covered the fibrous paper network, increasing grease resistance from Kit 0 to Kit 12 and reducing water vapour transmission by about 59%. These results confirm that the barrier response of starch-based coatings is strongly governed by surface coverage, film continuity, and formulation design rather than by starch alone.

Among starch-based systems, recent developments have also addressed functional limitations associated with converting operations, particularly heat sealability. In this context, Sadeghi et al. [49] investigated modified starch coatings based on sodium starch octenyl succinate (SSOS) and maltodextrin (MAL), demonstrating that the incorporation of plasticizers enables significant reductions in seal initiation temperature and fiber tear temperature, reaching values as low as 112–125 °C. These improvements are attributed to enhanced chain mobility, reduced glass transition temperature, and improved interaction with the fibrous network, which promotes effective sealing through mechanical interlocking and cohesive failure within the paper structure.

In addition to barrier-related properties, starch coatings significantly modify the functional behaviour of paper substrates. For instance, Jung et al. [50] reported a reduction in water absorption from approximately 105 to 40 g·m^−2^, together with an increase in oil resistance (Kit number from 1 to ~3) and improved tensile and burst strength. These effects are associated with the formation of a continuous surface layer that reduces porosity and limits fluid penetration.

Overall, starch-based coatings should therefore be interpreted as formulation-driven systems applied to paper substrates, whose effectiveness depends primarily on composition, intermolecular interactions, phase organisation, and coating microstructure rather than on starch alone.

The main structure–property relationships of starch-based mono-coating systems are summarised in Table 3.

A comparison between Table 2 and Table 3 highlights how cellulose-based coatings are primarily governed by film continuity and coating integrity, whereas starch-based systems are more strongly controlled by formulation and phase organisation.

#### 2.2.3. Alginate-Based Coatings

Alginate-based coatings represent another important class of polysaccharide systems for paper and board functionalisation. Due to its hydrophilic nature, alginate is able to form continuous films with good oxygen barrier performance, but its resistance to water vapour remains intrinsically limited, as confirmed by solution-cast film studies reporting WVTR values on the order of ≈250 g·m^−2^·day^−1^ [51].

When applied to paper substrates, alginate coatings exhibit behaviour that differs from both cellulose- and starch-based systems. Early studies demonstrated that alginate-based coatings can significantly modify the surface morphology of paperboard, producing smoother and more homogeneous structures while affecting water vapour permeability, water absorption, and mechanical behaviour [52]. However, the improvement in moisture barrier properties is strongly dependent on coating formulation and post-treatment, and alginate-based systems generally exhibit limited water resistance under humid conditions unless additional crosslinking or structural modifications are introduced.

More recent studies confirm that alginate-based coatings can act as effective functional barriers when applied to paper substrates. In particular, alginate coatings were shown to suppress air permeability to near-zero values, significantly improve grease resistance (Kit values up to 12), and strongly reduce the migration of hydrocarbon model compounds, highlighting their effectiveness as selective barrier layers [53]. At the same time, water vapour barrier performance remains comparatively limited and water uptake may even increase, reflecting the intrinsic hydrophilicity of alginate and its selective barrier behaviour.

To overcome these limitations, alginate systems are increasingly engineered through structural design strategies. This trend is also evident in alginate-based composite coatings incorporating inorganic functional fillers. In this regard, Kurtuluş et al. [54] investigated alginate/zinc oxide (ZnO) coatings applied to paper by bar coating and showed that the addition of ZnO improved dry strength, reduced air permeability, and imparted antibacterial activity against both and *E. coli*. At the same time, the results confirmed that the hydrophilic character of alginate remains critical, since water uptake remained high and wet strength decreased markedly after water exposure. Advanced composite coatings based on alginate combined with fibrous reinforcements and additional polymer layers, such as polyvinyl butyral (PVB), have been shown to drastically reduce water vapour transmission rates (from ~980 to ~50 g·m^−2^·day^−1^), demonstrating that barrier performance is primarily governed by coating architecture and pore structure rather than by the base polymer alone [55].

Overall, alginate-based coatings should therefore be interpreted as structure-controlled systems, in which performance depends strongly on ionic crosslinking, network organisation, and coating architecture. This behaviour distinguishes them from cellulose-based systems, which are primarily governed by film continuity, and from starch-based systems, which are mainly formulation-driven. The main structure–property relationships of alginate-based coating systems are summarised in Table 4.

#### 2.2.4. Chitosan Systems

Among polysaccharide-based coating materials, chitosan has emerged as one of the most versatile systems for paper and paperboard functionalisation, combining film-forming ability with intrinsic antimicrobial activity and strong affinity with cellulose substrates through hydrogen bonding and electrostatic interactions [24,56,57,58]. In paper-based packaging, polysaccharide coatings are typically applied to compensate for the intrinsic porosity of fibrous substrates through partial pore filling and interfacial sealing mechanisms [24].

From a structural perspective, the presence of amino and hydroxyl groups promotes hydrogen bonding and electrostatic interactions with cellulose fibers, enabling good adhesion and the formation of relatively dense coating layers. When applied to paper, chitosan coatings act by partially filling surface pores and modifying substrate morphology, leading to reduced air permeability, improved grease resistance, and limited or substrate-dependent improvements in oxygen barrier performance, while the overall barrier behaviour remains strongly influenced by the underlying porous fiber network [53,59]. In addition, improvements in tensile strength and structural integrity have been reported, indicating enhanced fiber–fiber bonding and surface consolidation [60].

A distinctive feature of chitosan-based systems is their multifunctionality, which distinguishes chitosan from other polysaccharides that are primarily passive barrier systems, particularly the combination of barrier performance and intrinsic antimicrobial activity, which is widely recognised in the literature [58]. Active coatings incorporating bioactive compounds have been shown to further improve grease resistance and water vapour barrier while providing protection against biological degradation mechanisms [61].

However, similarly to other hydrophilic polysaccharides, chitosan exhibits strong moisture sensitivity due to the abundance of hydroxyl and amino groups, leading to swelling and limited water vapour barrier performance under humid conditions, a limitation consistently reported in the literature [24,57,58].

To overcome these limitations, several structural and chemical modification strategies have been developed. Crosslinking approaches, such as the use of phytic acid, enable the formation of more compact and stable networks, improving water and oil resistance [62]. In parallel, grafting and hydrophobisation strategies based on chemical modification of the chitosan backbone, such as grafting with epoxidized soybean oil (ESO), reduce the exposure of hydrophilic groups and enhance water resistance and barrier performance [63].

Composite and hybrid systems further improve performance through synergistic interactions between components [64,65], while multilayer architectures combining chitosan with hydrophobic phases or structured coatings represent an effective route to enhance moisture resistance and overall barrier efficiency [66].

Experimental evidence on coated paper systems confirms the importance of formulation and network design. Inthamat et al. [67] showed that chitosan coatings significantly reduce water content, water vapour permeability, and oxygen permeability while improving tensile strength, with further enhancements achieved through the incorporation of astaxanthin and natural crosslinking agents such as genipin.

Overall, chitosan-based coatings should therefore be interpreted as multifunctional systems in which performance arises from the combined effects of pore sealing, interfacial interactions, mechanical reinforcement, and antimicrobial activity. While neat chitosan provides a useful baseline, practical applications increasingly rely on crosslinking, blending, and multilayer design strategies to overcome moisture sensitivity and achieve application-relevant barrier performance under realistic conditions. The main structure–property relationships of chitosan-based coating systems are summarised in Table 5.

#### 2.2.5. Comparison of Polysaccharide Coating Systems

In order to provide a comparative interpretation of the coating systems discussed above, a radar-based representation is introduced as a synthetic visual framework. This approach does not aim to derive averaged or directly comparable numerical values from the data reported in Table 2, Table 3, Table 4 and Table 5 but rather to capture the dominant trends emerging from the literature.

The available experimental data are inherently heterogeneous, as they are obtained under different testing conditions, measurement techniques, coating formulations, and substrate characteristics. In addition, performance is often reported through different metrics (e.g., OTR, WVTR, air resistance, grease resistance indices), and complete datasets are not consistently available for all systems. Under these conditions, direct quantitative comparison or statistical averaging would not be methodologically appropriate.

For this reason, the radar representation is based on a qualitative-to-semi-quantitative interpretative approach, in which performance levels are assigned according to recurring trends observed across the studies, rather than individual values. The assigned levels (low, medium, high) reflect the frequency, intensity, and robustness of the reported behaviour for each coating family.

The evaluation is performed with respect to the uncoated paper substrate, which is adopted as a common reference. Consequently, the radar is not based on inter-material normalization, but on independent assessments of the relative improvement provided by each coating system. This implies that similar performance levels may be assigned to different material families when comparable trends are observed.

Although Table 2, Table 3, Table 4 and Table 5 also include selected intrinsic or model-film systems to clarify the underlying material potential, these systems are not considered in the radar-based comparative framework.

Based on these assumptions, the radar representation provides a structured overview of the structure–property relationships governing polysaccharide-based coatings, highlighting both the specific strengths of each material family and the absence of a universally optimal solution across all functional requirements.

**Figure 7:**
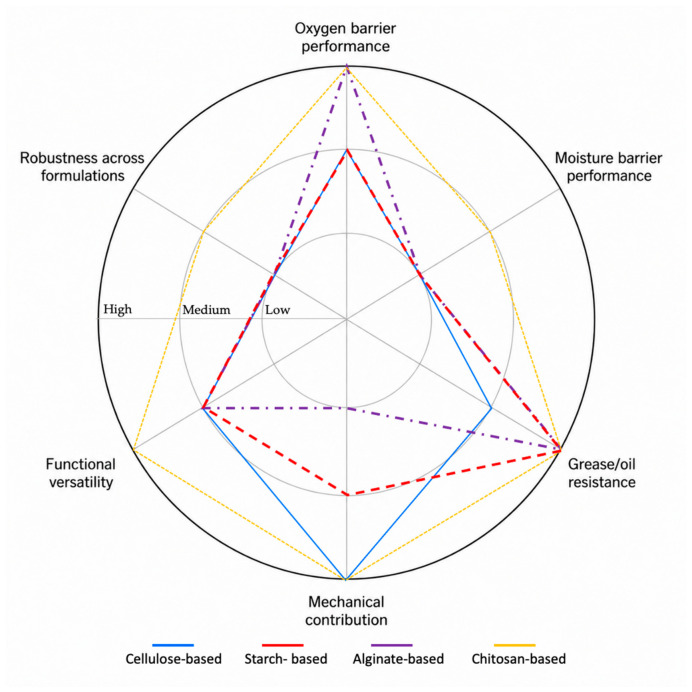
**Qualitative comparative radar of the main polysaccharide coating families for paper-based packaging.** Performance levels (low, medium, high) are defined relative to the uncoated paper substrate and are based exclusively on real coating systems applied to paper or paperboard, excluding intrinsic model-film data. The representation reflects the dominant trends emerging from Table 2, Table 3, Table 4 and Table 5 and the associated discussion, and is constructed through a qualitative-to-semi-quantitative interpretative approach that accounts for the heterogeneity of experimental conditions, measurement methods, and reported metrics. The radar highlights the complementary behaviour of cellulose-, starch-, alginate-, and chitosan-based coatings across oxygen barrier, moisture resistance, grease/oil resistance, mechanical contribution, functional versatility, and robustness across formulations.

The resulting comparative radar representation is shown in Figure 7.

The radar representation in Figure 7 provides a comparative overview of the relative performance of the main polysaccharide coating families with respect to the uncoated paper substrate. It should be emphasised that the reported performance levels reflect qualitative improvements over the control rather than absolute material properties.

As shown in the figure, cellulose-based coatings exhibit a pronounced contribution to mechanical performance, associated with fiber network densification and improved inter-fiber bonding, while providing moderate oxygen barrier improvements under real coating conditions. However, their effectiveness remains limited in terms of moisture resistance and robustness across different formulations.

Starch-based systems display a more balanced but generally moderate profile, with a clear advantage in grease and oil resistance, mainly due to pore filling and surface sealing effects. At the same time, their performance is strongly dependent on formulation and modification strategies, particularly to mitigate moisture sensitivity.

Alginate-based coatings show a more selective behaviour, characterised by relatively high oxygen barrier and grease resistance, but significantly limited mechanical contribution and poor resistance to water vapour. This reflects the intrinsic hydrophilic nature of alginate and its sensitivity to environmental humidity.

Chitosan-based systems exhibit the most extended performance profile, combining high mechanical contribution, good coating continuity, and enhanced functional versatility. In addition to barrier effects, the presence of intrinsic antimicrobial activity further expands their functional potential. Nevertheless, moisture sensitivity remains a limiting factor, although less pronounced compared to other polysaccharide systems.

Overall, the radar highlights that no single polysaccharide family simultaneously maximises all performance parameters. Instead, each system provides specific improvements relative to the control, confirming that performance optimisation in paper-based coatings relies on formulation design, chemical modification, and the development of hybrid or multilayer systems.

Beyond the main polysaccharide families discussed above, other systems have also been explored in the literature. Among these, pectin has been extensively investigated in the context of bio-based films and coatings for food packaging, owing to its film-forming ability, biodegradability, and potential gas barrier performance [69,70,71]. However, similarly to other hydrophilic polysaccharides, pectin exhibits limited water vapour barrier properties and strong sensitivity to humidity, which constrain its standalone application in barrier systems.

In addition, although widely studied in model films and edible coating systems, its use as a dedicated coating material for paper and paperboard substrates remains comparatively less developed.

Natural gums (e.g., guar gum, xanthan gum, and gum arabic) are also frequently reported in the broader literature on bio-based films and coating formulations, where they mainly contribute to film formation and rheological control [28,31]. In paper and paperboard coating systems, however, their role is typically secondary, as they are more often used as formulation modifiers or blending components rather than as standalone barrier layers.

Overall, this framework is consistent with the broader literature on bio-based barrier coatings for paper and paperboard, where cellulose, chitosan, starch, and related polysaccharide systems emerge as the most relevant candidates for coating applications, while other polysaccharides are less frequently reported as primary coating materials [24].

### 2.3. Lipid-Based Coatings

Lipid-based coatings represent a complementary strategy to polysaccharide-based systems for improving the barrier performance of paper-based substrates. While polysaccharide coatings are typically effective against gases under dry conditions, lipid-based materials are primarily associated with hydrophobicity, wetting control, and moisture-related barrier performance [6,7,9]. Lipid-based coatings should be interpreted as surface-controlled barrier systems, where transport is governed by interfacial phenomena rather than bulk diffusion.

From a mechanistic perspective, lipid-based coatings are dominated by surface-controlled transport mechanisms, where reduced surface energy suppresses wetting and capillary-driven liquid penetration. In contrast, protein-based systems rely on the formation of cohesive films, which can effectively reduce gas permeability under dry conditions but are strongly affected by moisture-induced plasticisation.

These transport-control mechanisms are schematically illustrated in Figure 5, which provides a conceptual framework linking material classes to barrier behaviour. In particular, Panel (2) represents surface-controlled hydrophobic effects typical of lipid-based coatings, whereas Panel (3) illustrates diffusion-controlled behaviour through cohesive films, characteristic of protein-based coatings.

In practice, single-component coating systems rarely provide sufficient multifunctionality under demanding packaging conditions. This distinction highlights how different classes of bio-based coatings control transport through fundamentally different mechanisms, ranging from bulk diffusion in dense polymer networks to interfacial and surface-controlled phenomena in lipid-based systems. As a result, combinations of protein matrices with hydrophobic components (e.g., lipid phases) are frequently adopted to balance barrier performance, processability, and end-of-life requirements [72].

Lipid-based coatings, including natural waxes, fatty acids and related derivatives, represent one of the most established strategies for improving the water resistance of paper and paperboard substrates. Their effectiveness arises from their intrinsically low polarity and reduced surface energy, which limit water sorption, reduce wettability, and suppress capillary-driven penetration into the porous fibrous network [73,74].

**Figure 8:**
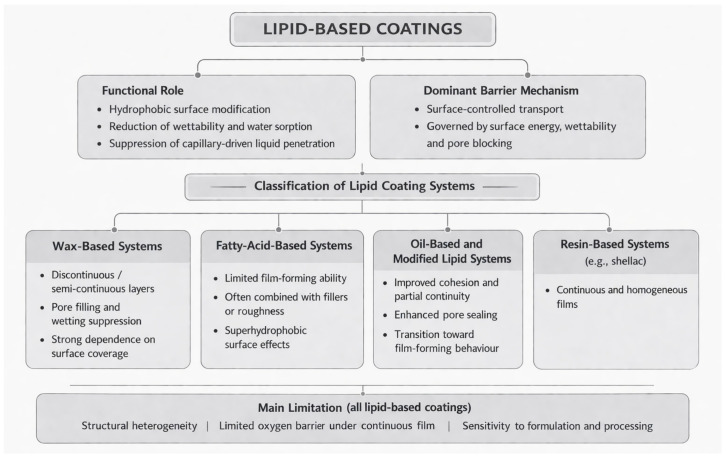
**Schematic classification of lipid-based coating systems for paper and paperboard, highlighting their structural organisation and dominant barrier mechanisms.** The systems are grouped into wax-based, fatty-acid-based, oil-based and chemically modified lipid coatings, and resin-based systems. The diagram illustrates the progressive transition from purely surface-controlled behaviour, governed by wettability and pore blocking, to partially diffusion-influenced transport in more structured or composite systems.

Lipid-based coatings encompass a broad range of materials, including waxes, fatty acids, oils and natural resins, whose barrier performance is primarily governed by their hydrophobic nature and structural organisation and phase distribution [75]. A schematic overview of this classification and its mechanistic evolution is provided in Figure 8. When applied as mono-coatings, these systems predominantly act through interfacial mechanisms rather than by forming dense diffusion-limiting networks. As schematically illustrated in Figure 5 (Panel 2), their behaviour is typically associated with a surface-controlled barrier mechanism, where performance is governed by wettability, surface energy, and pore blocking rather than bulk transport resistance. This behaviour is also related to the limited ability of lipid materials to form continuous and cohesive films, which differentiates them from polymeric coating systems governed by bulk diffusion.

In lipid-based systems, coating technologies primarily aim at modifying surface energy and pore accessibility, rather than creating dense diffusion-limiting layers. When applied to paper substrates, lipid coatings typically act by partially filling surface pores and forming discontinuous or semi-continuous hydrophobic layers that reduce liquid water uptake.

#### 2.3.1. Wax-Based Systems

In wax-based systems, barrier performance is primarily governed by surface-controlled mechanisms. These coatings typically form discontinuous or semi-continuous layers that partially fill surface pores and reduce liquid water uptake, without fully sealing the fibrous network. As a result, their effectiveness is mainly associated with hydrophobicity and wetting control rather than with the formation of a continuous diffusion barrier.

Wax-based coatings can enhance this mechanism by combining low surface energy with partial pore blocking, fiber impregnation, and, in some cases, surface roughness effects. In this context, Jahangiri et al. [76] reported that wax-coated papers significantly improve moisture resistance by reducing water vapour transmission and increasing surface hydrophobicity, mainly through pore blocking and fiber impregnation rather than the formation of a fully continuous film.

Beyond conventional wax coatings, more structured systems have been developed to improve coating homogeneity and performance. Aguirre-Joya et al. [77] showed that candelilla-wax-based systems can be effectively incorporated into film-forming matrices, contributing to improved physicochemical stability and functional performance. Vijayan et al. [76] reported that beeswax-based coatings can achieve low WVTR values, on the order of tens of g·m^−2^·day^−1^, when integrated within continuous bio-based polymer matrices, highlighting the role of surface roughness and composition in controlling hydrophobicity. Similarly, stabilized wax emulsions, including Pickering systems, have demonstrated improved coating uniformity and barrier performance. In this context, Daizy et al. [78] reported that structured wax emulsions can significantly reduce Cobb values while maintaining stable hydrophobicity, highlighting the critical role of microstructural organization and emulsion stability.

Despite these improvements, wax-based coatings remain predominantly surface-controlled systems. Their effectiveness strongly depends on coating uniformity, surface coverage, and defect control, as discontinuities and microcracks act as preferential pathways for gas and vapour permeation.

#### 2.3.2. Fatty-Acid-Based Systems

Fatty-acid-based coatings represent a simplified subset of lipid-based systems, whose performance is typically enhanced through structural or chemical modification, due to their inherently limited film-forming ability.

Hu et al. [79] demonstrated that stearic-acid-based coatings combined with mineral fillers (e.g., CaCO_3_) can generate superhydrophobic surfaces with contact angles up to ~150°, resulting from the combined effect of low surface energy and micro-scale roughness. In this case, barrier performance remains primarily surface-controlled, as the effect is dominated by wetting suppression rather than the formation of continuous films.

More advanced systems based on chemically modified fatty acids or lipid-derived compounds further improve barrier performance through increased coating cohesion and structural organization.

#### 2.3.3. Oil-Based and Chemically Modified Lipid Systems

Oil-based coatings and chemically modified lipid systems exhibit intermediate behaviour between surface-controlled coatings and more structured barrier layers, as their performance increasingly depends on coating continuity and cohesion.

Arsh et al. [80] reported that maleic anhydride-grafted camelina oil coatings can form relatively homogeneous layers on paper substrates, reducing water vapour permeability by up to 94% compared to uncoated paper, while also improving oxygen barrier performance and surface hydrophobicity.

Similarly, Ren et al. [81] demonstrated that fatty-acid-derived ester systems in emulsion-based coatings can significantly reduce water absorption (Cobb < 2 g·m^−2^), indicating effective pore sealing. Silva et al. [82] further showed that tung-oil-based coatings can increase contact angle from ~71° to ~127° and reduce water uptake from ~108 to ~17 g·m^−2^, confirming the role of improved film formation.

Chemically modified systems can also form crosslinked networks. Tambe et al. [83] demonstrated that silylated soybean oil coatings generate siloxane-based structures upon curing, improving coating cohesion, stability and moisture resistance. Consistently, Chen et al. [84] showed that castor-oil-based coatings can form continuous hydrophobic networks strongly bonded to cellulose substrates, achieving contact angles of ~114° and reducing WVTR by over 40%, while also improving mechanical strength.

These systems therefore represent a transition toward film-forming behaviour, where barrier performance progressively shifts from surface-controlled effects to structure-controlled transport mechanisms.

#### 2.3.4. Resin-Based Systems (Transition to Continuous Films)

More structured lipid-derived systems have been developed to address the intrinsic limitations of discontinuous coatings. Natural resins such as shellac represent a key example of this transition, as they can form continuous and homogeneous films on the substrate.

Khule et al. [85] reported that shellac-coated paperboard with two layers at 40% concentration exhibited very low water vapour permeability (1.3 × 10^−12^ kg·m·m^−2^·s^−1^·Pa^−1^) and oxygen permeability (3.8 × 10^−14^ kg·m·m^−2^·s^−1^·Pa^−1^), corresponding to improvements of 82.2% and 99.5%, respectively, compared to uncoated substrates. This behaviour reflects a transition toward a diffusion-controlled barrier mechanism associated with continuous film formation.

#### 2.3.5. Mechanistic Interpretation and Limitations

Despite their effectiveness against liquid water and grease, conventional lipid coatings often exhibit structural heterogeneity and limited adhesion to the substrate, resulting in non-uniform thickness and the presence of defects. Coating uniformity, surface coverage and defect control therefore represent critical parameters governing overall barrier performance, as discontinuities and microcracks act as preferential pathways for gas permeation.

From a mechanistic perspective, lipid-based coatings evolve along a continuum consistent with the framework illustrated in Figure 5 (Panel 2), ranging from surface-controlled regimes (discontinuous wax and fatty-acid systems dominated by wettability and pore blocking) to intermediate regimes (structured wax systems and modified oils with increasing continuity) and ultimately to diffusion-controlled regimes (resin-based and crosslinked systems forming continuous films that limit mass transport).

Overall, while lipid-based coatings are highly effective in limiting liquid water and grease penetration, their gas barrier performance remains limited unless defect-free and sufficiently continuous films are achieved.

Rather than achieving ultra-low permeability, these systems typically adjust moisture transport into application-relevant ranges, consistent with the permeability requirements of fresh produce and bakery packaging. The obtained WVTR values fall within the typical requirements for packaging of fruits and vegetables (~10–3000 g·m^−2^·day^−1^) and bakery products (~620–2800 g·m^−2^·day^−1^) [86].

The main structure–property relationships of lipid-based mono-coating systems are summarised in Table 6.

Discontinuous wax and fatty-acid-based systems are primarily governed by wettability and pore blocking, providing effective resistance to liquid water but a limited gas barrier. As the coating organization improves, structured wax systems and modified oils exhibit intermediate behaviour, where partial continuity enhances barrier performance. Finally, resin-based systems such as shellac approach continuous film formation, enabling a transition toward diffusion-controlled transport and significantly improved overall barrier properties.

This progression confirms that, in lipid-based coatings, performance is primarily dictated by structural organization rather than solely by the chemical nature of the material itself, distinguishing these systems from polysaccharide-based coatings, where barrier behaviour is more directly linked to intrinsic polymer properties.

### 2.4. Protein-Based Coatings

Protein-based coatings represent a distinct class of bio-based systems characterised by their ability to form dense, cohesive and structurally continuous films on paper and paperboard substrates. Unlike lipid-based coatings, which are primarily governed by interfacial phenomena, protein-based systems derive their barrier performance from the formation of compact polymer networks that limit molecular diffusion.

From a structural perspective, proteins consist of amino acid chains containing polar functional groups, which promote strong intermolecular interactions such as hydrogen bonding, electrostatic interactions and, in some cases, covalent crosslinking. As a result, protein-based coatings can form low free-volume networks that provide excellent gas barrier properties under dry conditions. However, due to their intrinsic hydrophilicity, these systems exhibit high sensitivity to moisture, which induces plasticisation and significantly reduces barrier performance, particularly against gases [87].

**Figure 9:**
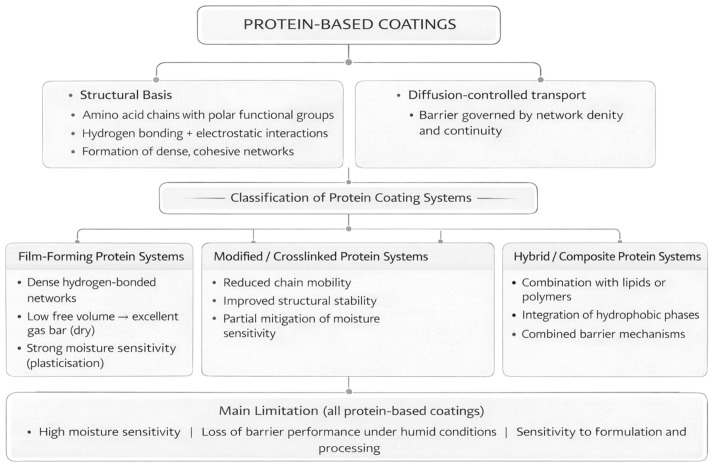
**Schematic classification of protein-based coating systems for paper and paperboard, highlighting their structural organisation and dominant barrier mechanisms**. The systems are grouped into film-forming protein coatings, modified and crosslinked systems, and hybrid or composite coatings. The diagram emphasises the transition from diffusion-controlled transport in dense protein networks to stabilised and combined mechanisms, as well as the intrinsic sensitivity of protein-based systems to moisture.

Protein-based coatings can be classified into three main typologies according to their structural organization and dominant barrier mechanism: (i) film-forming protein systems based on dense hydrogen-bonded networks, (ii) modified or crosslinked protein coatings with improved structural stability, and (iii) hybrid or composite systems combining proteins with other components such as lipids or polymers. A schematic overview of this classification and the associated barrier mechanisms is provided in Figure 9. This classification reflects the transition from diffusion-controlled behaviour in dense protein networks to combined mechanisms in hybrid systems. In these systems, barrier performance is primarily governed by the spatial distribution and continuity of the hydrophobic phase, rather than by the intrinsic properties of the protein matrix alone.

As schematically illustrated in Figure 5 (Panel 3), these systems are typically associated with a diffusion-controlled barrier mechanism, where transport is governed by network density and continuity rather than surface effects. In this context, only non-nanostructured mono-coating systems are considered in order to isolate the intrinsic behaviour of protein-based coatings.

#### 2.4.1. Film-Forming Protein Systems

The most characteristic behaviour of protein-based coatings is observed in systems capable of forming dense and continuous networks.

Whey protein isolate (WPI) coatings represent one of the most extensively studied high-barrier protein systems. Schmid et al. [88] reported oxygen transmission rates below 2 cm^3^·m^−2^·day^−1^·bar^−1^ (normalized to 100 μm), approaching the performance of synthetic high-barrier materials. This performance is directly associated with the formation of highly dense, crosslinked protein networks with reduced free volume, which significantly limit gas diffusion.

Consistently, Khwaldia et al. [87]. showed that sodium caseinate (NaCAS) coatings applied to paper form homogeneous and continuous layers, leading to significant improvements in oxygen barrier performance and mechanical properties due to the development of dense cohesive networks.

A similar mechanism was reported by Santos Lima et al. [34], who demonstrated that gelatin coatings applied to paper substrates can effectively fill surface pores and generate continuous films, resulting in very low water vapour transmission under controlled conditions and improved gas barrier performance.

More generally, protein systems such as whey, soy and gelatin exhibit excellent oxygen and aroma barrier properties under dry conditions, combined with good film-forming ability and mechanical performance.

However, their hydrophilic nature results in high sensitivity to moisture. Water uptake induces plasticization of the network, increasing chain mobility and significantly reducing barrier performance, particularly against gases.

#### 2.4.2. Modified and Crosslinked Protein Systems

To mitigate these limitations, protein-based coatings are often modified through chemical, physical or structural approaches.

Crosslinking strategies enhance intermolecular interactions and improve structural stability, resulting in reduced water sensitivity and improved barrier retention under humid conditions. In parallel, plasticizers are commonly introduced to improve flexibility and processability, although this typically leads to increased permeability due to higher free volume.

More recently, Warrier et al. [89] showed that the incorporation of microscale reinforcing phases in dairy protein coatings (caseinate and whey protein) applied to paper—such as uncoated kraft paper (UKP)—leads to reduced water uptake, increased hydrophobicity and improved mechanical stability. These effects are associated with a densification of the protein network and a reduction in accessible transport pathways, while maintaining the overall structural characteristics of protein-based coatings. Despite the extensive use of crosslinking and structural modification in protein-based films, their application to paper-based non-nanostructured mono-coating systems remains less extensively reported at the coating scale compared to material-level studies. These systems therefore represent stabilized protein networks, in which barrier properties are less sensitive to environmental conditions but remain governed by the underlying polymer structure.

#### 2.4.3. Hybrid and Composite Protein-Based Coatings

Hybrid systems combine protein matrices with other components to achieve balanced performance.

Marzbani et al. [90] demonstrated that soy protein isolate (SPI) coatings combined with polyethylene wax significantly improve moisture resistance while maintaining acceptable mechanical properties. Similarly, Nassar et al. [91] showed that whey protein coatings blended with polyvinyl alcohol lead to a significant reduction in water permeability (≈60%) and improved mechanical performance, due to the formation of more cohesive and flexible networks.

More broadly, protein-based coatings are frequently combined with lipids or polymeric phases to integrate hydrophobic barrier properties with the intrinsic gas barrier performance of proteins (Paidari, 2021) [92]. In this context, Khwaldia et al. [87] reported that the addition of a wax phase to protein coatings significantly reduces water vapour permeability, highlighting the complementary role of hydrophobic components.

These systems exhibit combined mechanisms, where diffusion-controlled behaviour of the protein network is complemented by surface-controlled effects introduced by hydrophobic phases.

#### 2.4.4. Mechanistic Interpretation and Limitations

From a mechanistic perspective, protein-based coatings are predominantly governed by diffusion-controlled transport through dense and cohesive polymer networks, although structural modification and hybridisation can introduce additional stabilising or complementary barrier mechanisms. However, their high gas-barrier performance is generally retained only under dry or controlled-humidity conditions, while moisture sensitivity remains the main limitation requiring crosslinking, blending, or hybridisation strategies.

The main structure–property relationships of protein-based coating systems are summarised in Table 7.

The systems reported in Table 7 clearly highlight the fundamental structure–property relationships governing protein-based mono-coatings. Across all cases, the dominant feature is the ability of protein matrices to form dense and cohesive networks, which consistently result in excellent oxygen barrier performance under dry conditions. This behaviour is directly associated with strong intermolecular interactions and reduced free volume, confirming the diffusion-controlled transport mechanism typical of protein-based systems.

As illustrated in Figure 5 (Panel 1), protein-based coatings are consistently governed by a diffusion-controlled regime, in which barrier performance is primarily dictated by network density and continuity. In this respect, all film-forming systems exhibit a coherent mechanistic behaviour, regardless of the specific protein source.

At the same time, Table 7 highlights the main limitation of these systems, namely their pronounced sensitivity to moisture. In all cases, increasing relative humidity leads to plasticisation of the protein network, resulting in increased chain mobility and a marked reduction in barrier performance, particularly against gases. This behaviour represents an intrinsic characteristic of protein-based coatings and is consistently observed across different systems.

Modified and crosslinked coatings partially mitigate this limitation by enhancing structural stability and reducing water uptake, thereby extending the effective operating range of the diffusion-controlled regime. However, these approaches do not fundamentally alter the governing transport mechanism.

In contrast, hybrid and composite systems introduce a transition towards combined barrier behaviour, where the diffusion-controlled mechanism of the protein matrix is complemented by surface-controlled contributions associated with hydrophobic or polymeric phases. This results in more balanced performance, particularly in terms of moisture resistance, although often at the expense of increased system complexity.

Overall, Table 7 confirms that protein-based mono-coatings are intrinsically high-performance gas barrier systems under controlled conditions, but require structural modification or hybridisation to ensure stable performance in realistic packaging environments. It should be noted that, due to the diversity of substrates used in the literature (e.g., paper, paperboard, or polymer films), reported performance values are often relative to the corresponding uncoated reference system.

### 2.5. Bio-Based and Synthetic Polymer Coatings

Bio-based and synthetic polymer coatings represent a key class of materials for paper functionalisation, enabling the formation of continuous films that effectively modify the porous structure of fibrous substrates. These systems bridge the gap between purely bio-derived coatings and industrially established coating technologies, encompassing a wide range of materials including water-soluble polymers (e.g., PVA, PVOH), bio-based polyesters, waterborne polymer dispersions such as acrylic and styrene–acrylate systems, and conventional fossil-based thermoplastics [93,94].

These materials are widely employed due to their ability to form continuous or near defect-free films with controlled thickness and strong adhesion to cellulosic substrates, enabling improvements in both barrier performance and mechanical integrity [6,7,9,14,24]. A representative example is provided by Hamdani et al. [95], who showed that functionalized crystallizable polylactic acid (CPLA) and crystallizable poly(butylene adipate-co-terephthalate) (CPBAT) waterborne coatings can improve moisture barrier performance while retaining repulpability and compostability. Unlike lipid- or polysaccharide-based systems, these coatings form continuous or semi-continuous polymer films, enabling barrier performance governed by a solution–diffusion mechanism [96]. The incorporation of CPBAT was found to suppress the intrinsic brittleness of PLA, leading to defect-free coating layers and significantly enhanced barrier properties. In particular, the optimized CPLA/CPBAT (20/80) formulation reduced water absorption from approximately 62.6 to 9.2 g/m^2^ (Cobb600) and decreased WVTR by up to 95. However, it is widely recognised that single-component bio-based coatings rarely provide balanced resistance to moisture, oxygen, and grease simultaneously and therefore often require multilayer or composite solutions to meet the most demanding packaging requirements [97].

**Figure 10:**
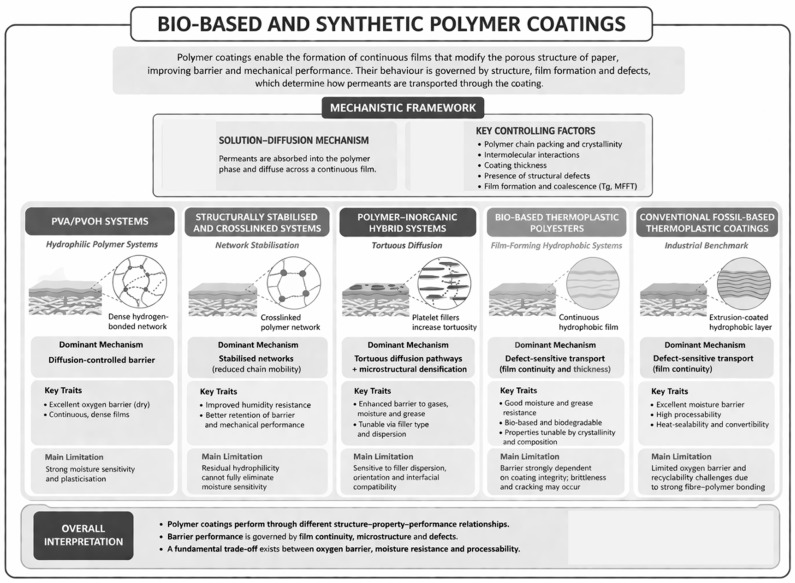
**Schematic classification of polymer coating systems for paper and paperboard, based on their dominant structure–property relationships and barrier mechanisms**. The systems are grouped into hydrophilic polymers (PVA/PVOH), structurally stabilised and crosslinked systems, polymer–inorganic hybrid systems, bio-based thermoplastic polyesters, and conventional fossil-based thermoplastic coatings. The diagram highlights the key mechanisms governing barrier performance, including diffusion-controlled transport, network stabilisation, tortuous diffusion pathways, and defect-sensitive film behaviour, providing a simplified overview of the classification adopted in this section.

Within this framework, coating systems are classified into five categories: (i) hydrophilic PVA/PVOH systems, (ii) crosslinked systems, (iii) polymer–inorganic hybrids, (iv) bio-based thermoplastic polyesters, and (v) fossil-based thermoplastic coatings. Figure 10 summarises the classification and associated barrier mechanisms, while Table 8 provides a comparative overview of their performance. Overall, these categories describe a transition from molecularly controlled barrier systems, such as hydrogen-bonded PVOH networks, to microstructure- and defect-controlled systems, such as hybrid coatings and thermoplastic films.

Across these systems, barrier performance is strongly influenced by the formation of uniform and continuous coatings, although the relative importance of molecular packing, interfacial adhesion, filler dispersion and defect control varies among the different classes. As illustrated in Figure 5 (Panel 3), permeability depends on polymer chain packing, crystallinity, intermolecular interactions, and coating thickness, as well as on the presence of structural defects.

At the same time, film formation plays a critical role in determining coating performance. In waterborne systems, particularly those based on latex dispersions, film formation occurs through particle deformation and coalescence, with key parameters such as glass transition temperature (Tg) and minimum film formation temperature (MFFFT) governing coating integrity (Solera-Sendra, 2025, Pieters, 2024) [98,99]. However, residual interparticle boundaries, incomplete coalescence, or poor wetting of the fibrous substrate may introduce preferential permeation pathways, so that the final barrier response is not governed solely by intrinsic polymer permeability. As a result, coating uniformity, substrate interaction, and defect generation can significantly influence barrier performance, even when intrinsic polymer properties are favourable [100], while stabilised and hybrid systems can reduce defect-driven permeation pathways and improve resistance to moisture and grease [101].

The representative systems discussed in the following subsections and summarised in Table 8 illustrate the diversity of barrier responses achievable through different polymer coating strategies.

#### 2.5.1. Pva/Pvoh Systems

Poly(vinyl alcohol) (PVA/PVOH)-based coatings represent the reference class of hydrophilic polymer systems for paper functionalisation, characterised by the formation of dense, continuous films with excellent oxygen barrier performance under dry conditions. In these systems, barrier properties are governed by a diffusion-controlled mechanism associated with strong intermolecular hydrogen bonding and reduced free volume within the polymer network.

This behaviour is consistent with previous studies on PVOH-based barrier coatings, where the formation of dense hydrogen-bonded networks has been identified as the primary factor controlling oxygen permeability under dry conditions [9,24].

This behaviour is well illustrated in practical coating applications, where PVOH-based systems are widely used to provide oxygen and grease barrier properties in paper and paperboard. As reported by Michel (2025) [102], increasing coating weight promotes the formation of dense and continuous films, leading to a drastic reduction in oxygen transmission rate, with oxygen transmission rate decreasing by several orders of magnitude as coating weight increases and continuous films are formed. At the same time, air resistance increases significantly and grease resistance is improved, confirming the effectiveness of these coatings in limiting mass transport through the fibrous structure.

From a structural perspective, this behaviour is directly associated with the semi-crystalline nature of PVOH and the presence of strong intermolecular interactions, which promote tight chain packing and significantly reduce the free volume available for gas diffusion. As a result, these systems represent the most effective polymer-based mono-layer coatings for oxygen barrier applications on paper substrates.

Due to their intrinsic hydrophilicity, these materials readily absorb moisture, which leads to plasticisation of the polymer network, increased chain mobility, and a progressive loss of barrier performance, particularly for gases. This sensitivity to relative humidity represents the main limitation of hydrophilic polymer coatings and restricts their applicability under realistic service conditions.

To overcome these limitations, structurally stabilised systems based on crosslinking and network modification strategies have been developed to enhance resistance to moisture and improve barrier retention, as discussed in the next section.

#### 2.5.2. Crosslinking and Structural Stabilisation

To mitigate the intrinsic humidity sensitivity of hydrophilic polymer coatings, crosslinking strategies are widely employed to stabilise the polymer network and reduce chain mobility. In these systems, the diffusion-controlled barrier mechanism typical of PVOH-based coatings is preserved, while structural stability is enhanced through the formation of additional intermolecular or covalent interactions.

This approach is exemplified by boric acid (BA)–crosslinked PVA using epichlorohydrin (ECH) as a binder coating reported by Choe et al. [103], in which the introduction of borate linkages and chemical crosslinking leads to a dense and stabilised network. As a result, a significant reduction in both water vapour and oxygen permeability is achieved, together with improved mechanical strength and oil resistance, with grease resistance increasing from kit values of 1 to 12. Importantly, these systems exhibit improved retention of barrier performance under humid conditions compared to non-crosslinked coatings. A similar strategy was reported by Kwon et al. [104], where oxalic acid-modified poly(vinyl alcohol) (POA) crosslinked with Ca^2+^ ions enabled the formation of stable barrier coatings on paper substrates. In this system, crosslinking occurred during drying, allowing compatibility with industrial water-based coating processes while improving resistance to moisture and maintaining barrier performance.

From a mechanistic standpoint, crosslinking reduces the mobility of polymer chains and limits water-induced plasticisation, thereby maintaining a lower free volume and preserving barrier properties. However, the effectiveness of this strategy depends strongly on the nature and density of the crosslinked network, as well as on coating uniformity.

Despite these improvements, crosslinked systems still retain a degree of hydrophilicity and therefore cannot completely eliminate moisture sensitivity. This limitation has driven the development of hybrid systems incorporating inorganic fillers to further enhance barrier performance, as discussed in Section 2.5.3.

#### 2.5.3. Polymer–Inorganic Hybrid Systems

Polymer–inorganic hybrid coatings represent an extension of structurally stabilised systems in which barrier performance is further enhanced through the incorporation of nanostructured fillers. In these systems, the dominant mechanism shifts from purely diffusion-controlled transport to a combined effect involving tortuous diffusion pathways generated by high-aspect-ratio fillers such as clay platelets.

This behaviour has been widely demonstrated in nanoclay-filled PVA systems, where the dispersion of layered silicates within the polymer matrix significantly reduces permeability by increasing the effective diffusion path length [36,105]. Similarly, Zhang et al. [106] reported PVA–bentonite coatings forming compact “brick-and-mortar” structures through coordination interactions between the polymer and the inorganic phase, resulting in improved resistance to oxygen, water vapour, and oil, together with enhanced mechanical performance. A further example of polymer–inorganic hybrid coatings is provided by Achrekar et al. [107], who investigated PVOH-based dispersion coatings incorporating combinations of lamellar and spherical inorganic fillers, including kaolin clay, calcium carbonate and fumed silica. In these systems, barrier performance is governed by a synergistic mechanism combining tortuous diffusion pathways and microstructural densification. While single-filler coatings exhibited limited performance, optimized hybrid systems (e.g., kaolin/silica) achieved oxygen transmission rates as low as 5.6 cc/m^2^·day and water vapour transmission rates of approximately 5 g/m^2^·day. These results highlight the importance of combining fillers with different geometries, where platy particles increase diffusion path length and spherical particles reduce voids and improve packing density, leading to significantly enhanced barrier properties.

From a microstructural perspective, barrier enhancement in these systems arises from a synergistic combination of reduced polymer mobility and increased tortuosity, which limits mass transport across the coating. A comparable behaviour has been observed in inorganic–organic hybrid coatings based on silicone oil–modified silica dispersed in a poly(methyl methacrylate) (PMMA) matrix, where the combination of low surface energy and micro/nano-scale surface roughness leads to highly hydrophobic paper substrates, with water contact angles up to ~142° and a significant reduction in water vapour transmission [108]. However, the effectiveness of this mechanism depends critically on filler dispersion, orientation, and interfacial compatibility with the polymer matrix.

Despite their high barrier performance, hybrid systems introduce additional complexity in formulation and processing, and their properties may be sensitive to aggregation or non-uniform filler distribution. These aspects distinguish them from purely film-forming systems, in which barrier behaviour is governed primarily by coating continuity, as discussed in Section 2.5.4.

#### 2.5.4. Bio-Polyesters and Film-Forming Hydrophobic Systems

Bio-based thermoplastic polyesters—such as polylactic acid (PLA), poly(butylene succinate) (PBS), poly(3-hydroxybutyrate-co-3-hydroxyvalerate) (PHBV), and polyhydroxyalkanoates (PHA)—represent a distinct class of coatings characterised by their ability to form continuous hydrophobic films on paper substrates. In contrast to hydrophilic polymer systems, barrier performance in these materials is governed primarily by film continuity and defect control rather than molecular packing effects alone.

This behaviour is clearly illustrated by PLA-based coatings, where increasing coating weight leads to improved surface coverage and a significant reduction in both water vapour transmission and air permeability, as demonstrated by Abdenour et al. [109]. A further example of bio-based thermoplastic coatings is provided by Cao et al. [110], who developed spray-coated PLA/PHA systems applied onto kraft paper substrates, followed by hot pressing to improve coating uniformity. In these systems, the two polymers play complementary roles: PLA forms a dense and continuous surface layer that effectively seals the porous structure, while PHA penetrates into the fibrous network, filling internal voids and enhancing interfacial cohesion. As a result, the optimized composition (PLA/PHA 50:50) achieved the lowest oxygen transmission rate among the tested compositions, significantly improving barrier performance compared to both uncoated paper and single-component coatings. However, excessive PHA content led to morphological defects and discontinuities, resulting in a deterioration of barrier properties, highlighting the critical role of composition in controlling film integrity and transport mechanisms. A similar mechanism has been reported by Gregor-Svetec et al. [111], where solution-applied PLA coatings formed a continuous coating layer that significantly improved water resistance compared to uncoated paper. In these systems, barrier performance is strongly correlated with coating thickness and the formation of continuous films that effectively seal the porous fibrous network.

Other bio-polyesters exhibit different structure–property relationships. For instance, PBS-based waterborne coatings reported by Hamdani et al. [112] provide good moisture and oil resistance, together with heat-sealability and industrial processability, although their gas barrier performance remains moderate. In contrast, PHBV coatings [113] show higher stiffness but are affected by brittleness and crack formation, which can significantly compromise barrier properties. More advanced systems based on PHA blends [114], mainly investigated as standalone films, demonstrate that mechanical and barrier properties can be tailored through crystallinity control, where increasing amorphous content enhances flexibility and processability while progressively increasing permeability, highlighting the need to balance barrier performance and mechanical behaviour through optimized blending ratios.

At a system level, extrusion-coated bio-polyester layers in poly(3-hydroxybutyrate) (PHB) or paperboard-based multilayer systems evaluated by Lev et al. [115] show good convertibility and moisture resistance, with low Cobb values and stable mechanical behaviour, but relatively high oxygen transmission rates, confirming the intrinsic limitation of these materials as gas barriers. A complementary approach based on waterborne processing is provided by Hämäläinen et al. [116], who developed bio-based PLA copolymer dispersions (PLAX) applied as single-layer coatings on paper substrates. The resulting coatings exhibited promising barrier performance, with water vapour transmission rates below 10 g/m^2^·day at 23 °C and 50% relative humidity (RH), depending on polymer molecular weight and formulation. In these systems, barrier performance is governed by the formation of continuous films from dispersed polymer particles, with coating integrity strongly influenced by particle size distribution and film coalescence. The results highlight the potential of aqueous dispersion-based polyesters as sustainable alternatives to extrusion coatings while also emphasizing the role of polymer design and processing conditions in controlling barrier performance.

Overall, bio-polyester coatings act as continuous film-forming systems that effectively reduce moisture transport by sealing the substrate, but whose barrier performance remains strongly dependent on coating integrity, thickness, and processing conditions. This behaviour is comparable to that of conventional thermoplastics, as discussed in Section 2.5.5.

#### 2.5.5. Conventional Fossil-Based Thermoplastic Coatings

Conventional fossil-based thermoplastics represent one of the most established industrial solutions for the functionalisation of paper and paperboard, particularly in the form of continuous layers applied by extrusion coating. The most relevant materials include polyethylene (PE), polypropylene (PP), and functional polyolefin-based copolymers such as poly(ethylene-co-vinyl acetate) (EVA) and poly(ethylene-co-acrylic acid) (EAA).

Polyethylene (PE), in both LDPE and HDPE forms, is the dominant material due to its good moisture barrier, heat-sealability, and processability [2,20,24]. When applied to paper, it forms continuous hydrophobic films that effectively seal the fibrous network, representing the industrial benchmark for moisture-resistant packaging. However, its oxygen barrier performance remains limited [9].

Polypropylene (PP) exhibits similar behaviour, with increased stiffness and thermal resistance, but its use in paper coating applications may be limited by adhesion and processing considerations [20].

Functional copolymers such as EVA and EAA are primarily used to improve adhesion and interfacial bonding rather than barrier performance [20].

Despite their performance advantages, these systems present significant recyclability challenges due to strong fiber–polymer bonding, which complicates material separation during recycling [9,24].

**Table 8:** **Representative bio-based and synthetic polymer coating systems for paper and paperboard.** Single-layer coatings are grouped into hydrophilic polymer systems, crosslinked systems, polymer–inorganic hybrids, bio-based thermoplastic polyesters, and conventional fossil-based thermoplastics. The table reports composition, processing route, and selected barrier/mechanical indicators, with quantitative values included when experimentally available.

Coating System (Reference)	Composition	Processing	WVTR (g/m^2^·day)	OTR	Mechanical/Converting	Key Functional Traits	End-of-Life/Compliance	Main Limitation
Hydrophilic polymer systems (PVOH/PVA)								
PVOH coating with fatty acid grafting—[117]	Polyvinyl alcohol (PVOH) modified via surface esterification with fatty acid chlorides (C16–C18)	Aqueous coating (rod/anilox) + drying + thermal grafting (~150 °C)	WVTR reduced from ~665–1046 to ~54–165 g/m^2^·day (BIF up to ~19)	Significantly improved under humid conditions (reduced oxygen permeability)	Stable coating; barrier retained under humidity; no significant bulk mechanical alteration	Hydrophobic surface layer formation; reduced moisture sorption; stabilization of diffusion-controlled barrier	Water-based system; compatible with paper recycling; surface modification approach	surface modification; grafting depth not determined; performance dependent on grafting density; defect-controlled transport
PVOH coating—[102]	Polyvinyl alcohol (PVOH) aqueous coating on paper	Size press/aqueous coating	Water absorption reduced from ~47.8 to ~25–39 g/m^2^ (Cobb60)	Very low OTR under dry conditions (<1 cm^3^·m^−2^·day^−1^), but strongly humidity-dependent, increasing by more than one order of magnitude above 70% RH	Air resistance increases from ~15 to up to 30,000 s; moderate improvement in grease resistance (KIT 1 → 2–3)	Dense continuous film; pore sealing; diffusion-controlled barrier strongly dependent on coat weight	Water-soluble; compatible with paper recycling	Strong humidity sensitivity; requires defect-free film and sufficient coat weight to achieve barrier
Structurally stabilised and crosslinked systems								
Oxalic acid–modified PVA coating—[104] *	Oxalic acid–modified PVA (POA) crosslinked with Ca^2+^ ions; optional CaCO_3_ nanoparticles (NPs)	Aqueous coating (bar coating) with in situ crosslinking during drying	~132 (POA–Ca^2+^), ~99 (POA–Ca^2+^/NPs) (monolayer, 3.0 g/m^2^)	~1.93 (POA–Ca^2+^), ~0.17 (POA–Ca^2+^/NPs) (monolayer, 3.0 g/m^2^)	Improved water resistance and coating stability due to ionic crosslinking; suitable for multilayer stacking	Ionic crosslinking (Ca^2+^–carboxylate) + optional tortuosity (NPs); reduced swelling vs. PVA	High repulpability (~99.7%); biodegradability up to ~91.8% (composting)	Moderate moisture barrier in monolayer; performance strongly enhanced only in multilayer configurations; values extracted from figures
Boric acid-crosslinked PVA coating—[103]	Kraft paper coated with PVA + BA + HCl, with ECH as binder (KP-P, KP-PB, KP-PBH)	Bar coating + drying/crosslinking	WVTR: N.A. (KP) → 50.67 (KP-P), 28.00 (KP-PB), 5.17 g m^−2^ d^−1^ (KP-PBH)	OTR: N.A. (KP) → 5.30 ± 0.50, 1.92 ± 0.04, 0.89 ± 0.03 cc m^−2^ d^−1^	Tensile strength: 31.4 → 53.0 MPa; 51.9 MPa retained at 80% RH; oil resistance: kit 1 → 12	Dense crosslinked coating; improved gas, vapor, and oil barrier	Biodegradable coated paper	More complex than mono-PVOH systems due to BA/HCl/ECH crosslinking
Polymer–inorganic hybrid systems								
Silicone oil-modified silica/PMMA coating—[108]	PMMA matrix with silicone oil–impregnated nano-silica (≈20 nm)	Dispersion preparation + dip-coating + drying at 60 °C	WVTR reduced from ~658 to ~395 g/m^2^·day (ASTM E96; not directly comparable with standard gas transmission data)	N.A. (gas barrier not primary; moisture-dominated behaviour)	Higher tensile strength and stiffness (tensile strength: ~1 → 3 N/mm; tensile index: ~13 → 28 N·m/g), with reduced elongation at break (~7 → 4%)	Hydrophobic hybrid coating; combined low surface energy and micro/nano roughness (Cassie–Baxter regime); pore coverage and partial sealing	Not bio-based; potential recyclability limitations due to polymer–fiber interaction	Limited gas barrier; performance primarily moisture-driven; dependent on filler dispersion and coating uniformity
PVOH + inorganic fillers (kaolin/CaCO_3_/silica)—[107]	Polyvinyl alcohol (PVOH) with lamellar (kaolin) and spherical (silica, CaCO_3_) fillers; glyoxal crosslinking	Aqueous dispersion + rod coating (two-pass) + drying	WVTR ≈ 5.0 g/m^2^·day; WVTR ≈ 150–300 g/m^2^·day; OTR > 400 cc/m^2^·day (monolayer coatings)	Tortuosity (platelets) + void filling (spherical fillers) + densified polymer network	Improved barrier and grease resistance; performance tunable via filler combinations; scalable waterborne process	Aqueous system; compatible with paper recycling; reduced plastic content	Poor monolayer performance; strong dependence on filler dispersion and defect control; formulation complexity	Barrier performance strongly dependent on filler dispersion and defect control; increased formulation complexity.
Bio-based thermoplastic polyesters								
PHBV coating—[113]	PHBV with optional plasticisers (TEC, PEG up to 15 wt%)	PHBV with optional plasticisers (TEC, PEG up to 15 wt%)	Not explicitly reported; limited barrier due to film cracking	Not reported	High stiffness; low elongation at break (~0.9–2.4%); moderate adhesion (bond strength up to ~1.2 N/15 mm)	Continuous thermoplastic film; barrier limited by structural defects (cracks)	Continuous thermoplastic film; barrier limited by structural defects (cracks)	Brittleness and crack formation; high minimum thickness (~30 µm); poor barrier reliability without modification
PBS coating—[112]	PBS (waterborne latex, PVOH-stabilised)	Dispersion coating (rod coating, thermal drying)	WVTR ≈ 113 g m^−2^ day^−1^ (significantly reduced vs. uncoated paper); Cobb ≈ 15.5 g m^−2^	Moderate OTR (not primary barrier mechanism)	Excellent heat-sealability; improved flexibility; good coating uniformity	Strong water and oil resistance (Kit 12); continuous film formation; comparable to commercial benchmarks	Biodegradable under composting; does not hinder paper degradation	Moderate gas barrier; requires sufficient coating uniformity and multilayer for full performance
Acrylic/styrene–acrylate dispersion coatings (class of materials)—[93,94,98,100,101];	Waterborne acrylic/styrene–acrylate/related latex dispersions, in some cases pigment-filled or resin-stabilized	Dispersion coating/bar or rod coating/industrial aqueous coating	Improved water barrier (values highly formulation-dependent; not directly comparable across studies)	Moderate to good oxygen barrier depending on film integrity; quantitative values vary widely across formulations	Good film formation; converting behaviour depends on coating integrity and defect generation	Continuous film via particle coalescence; tunable Tg/MFFT; adaptable industrial processing	Industrially relevant; typically not fully bio-based	Limited packaging-specific barrier data in general reviews; barrier strongly defect-sensitive on paper substrates
PLA coating—[109]	Polylactic acid (PLA) (reference: uncoated paper)	Extrusion coating/solution coating	WVTR ↓ (~419 → ~34 g·m^−2^·day^−1^)	OTR: N.A. (not reported; air permeability reduced)	Improved stiffness; continuous film; good processability	Continuous thermoplastic film; pore sealing; reduced permeability	Biodegradable; compatible with bio-based packaging systems, but industrial compostability and recycling compatibility depend on coating thickness and formulation	Limited oxygen barrier; sensitivity to pinholes and defects
PLA coating—[111]	PLA solution (15–20 wt%) applied on packaging paper	Solution coating (wire-wound bar), single-layer deposition	Cobb ≈ 0 g/m^2^ (no water absorption; strong reduction vs. base paper)	OTR: N.A. (not reported)	Reduced roughness; improved surface uniformity; slight optical variation	Continuous film formation; pore penetration and sealing of fibrous network	Bio-based and biodegradable coating; compatible with sustainable packaging	Limited oxygen barrier; performance dependent on coating uniformity and thickness
PLA copolymer dispersion (PLAX)—[116]	PLA-based copolymers (lactic acid + itaconic acid + butanediols) dispersed in water with PVA stabilizer	Solvent-free thermomechanical dispersion preparation; semi-pilot roll-to-roll rod coating	WVTR < 10 g/m^2^·day (23 °C, 50% RH); improved vs. base paper (e.g., 14.9 → 5.6 g/m^2^·day)	Not reported	Uniform coatings; performance dependent on particle size distribution and film coalescence	Aqueous dispersion system; solvent-free processing; tunable properties via copolymer design and dispersion parameters	Bio-based; compatible with paper recycling streams; scalable process demonstrated	Barrier strongly dependent on film formation and dispersion quality; moderate gas barrier; formulation complexity
PLA/PHA blend coating—[110]	PLA + PHA blends (25:75–75:25)	Spray coating + thermal drying + hot pressing (170 °C)	WVTR: not reported	OTR = 15.15 ± 1.25 cc/m^2^·day (optimal 50:50); PLA alone shows moderate barrier; blends improve performance depending on composition	Tensile strength improved vs. paper (up to 73 MPa for PHA-rich systems); 50:50 blend shows lower strength (~48 MPa) due to phase separation	Dual mechanism: PLA forms dense surface film (pore sealing); PHA penetrates fibrous matrix (void filling); morphology controlled by blend ratio	Biodegradable; bio-based system; compatible with sustainable packaging strategies	Excess PHA causes defects and discontinuities; phase separation; sensitivity to composition and processing
CPLA/CPBAT coating—[95]	Functionalized PLA (CPLA) + PBAT (CPBAT) (polyester blend)	Waterborne emulsion coating + drying (100 °C) + curing (~160 °C)	Cobb600: ~62.6 → ~9.2 g/m^2^ (~−85%); WCA up to ~77°	WVTR: ~1015 → ~40 g/m^2^·day (~−95%)	Improved flexibility (PBAT); tensile strength decreases ~10–22%; increased elongation; good sealability	Continuous polymer film; defect suppression via blending; solution–diffusion barrier	Recyclable and compostable (validated repulpability > 85%)	PLA brittleness (cracks if not blended); relatively high coating load (~40–50 g/m^2^)

* Values for Kwon et al. (2024) [104] refer to monolayer POA-based coatings extracted from graphical data (Figure 5) and are reported under specific testing conditions (OTR: 23 °C, 0% RH; WVTR: 37.8 °C, 100% RH).

Hydrophilic systems such as PVOH exhibit outstanding oxygen barrier performance under dry conditions due to dense hydrogen-bonded networks, but their effectiveness is strongly limited by moisture sensitivity.

Crosslinked and hybrid systems partially overcome this limitation by stabilising the polymer structure and, in the case of inorganic fillers, introducing tortuous diffusion pathways that further reduce permeability.

In contrast, thermoplastic coatings based on bio-polyesters and conventional fossil polymers act predominantly through pore sealing and film continuity, providing effective moisture resistance but generally limited oxygen barrier performance due to the higher free volume of polymer chains. Within this class, bio-based polyesters exhibit a wide range of behaviours depending on crystallinity, composition, and processing conditions.

It should be noted that the higher number of systems reported for bio-based thermoplastic polyesters reflects the growing research interest in sustainable alternatives to conventional coatings, rather than an intended imbalance in the selection of materials.

Overall, the table highlights a fundamental trade-off between oxygen barrier performance and environmental or processing stability, which governs the selection of polymer coatings for specific packaging applications.

### 2.6. Hybrid and Nanostructured Coatings

Hybrid and nanostructured coatings represent an advanced strategy for enhancing the performance of paper-based packaging systems, as they enable the control of transport phenomena through microstructural design in addition to the intrinsic properties of the base material. In these systems, barrier performance is governed not only by the chemical nature of the coating but also by structural organisation at the micro- and nanoscale, which determines diffusion pathways, interfacial interactions, and coating continuity.

In this context, it is essential to distinguish between nanostructured systems and hybrid systems, which are often used interchangeably but refer to fundamentally different concepts. Nanostructured coatings are defined as systems in which functional behaviour is governed by nanoscale structural features, even in the absence of multiple phases. A representative example is provided by nanocellulose-based coatings, which form dense and highly interconnected networks capable of significantly reducing free volume and enabling diffusion-controlled transport. Although derived from cellulose, these systems differ fundamentally from conventional cellulose-based coatings discussed in Section 2.2 as their performance arises from nanoscale organisation rather than from the intrinsic properties of the fibrous substrate.

This distinction is particularly important considering that paper itself is primarily composed of cellulose fibers. However, in paper substrates, cellulose is present in the form of a porous and heterogeneous fibrous network, characterised by open transport pathways and limited barrier performance. In contrast, nanocellulose-based coatings act as a functional phase capable of reorganising the microstructure, reducing pore connectivity, and enabling diffusion-controlled transport.

Hybrid coatings, in contrast, are multiphase systems in which a continuous matrix is combined with a dispersed phase, typically at the micro- or nanoscale. In these systems, barrier performance is governed by the interaction between phases, enabling additional transport-control mechanisms such as tortuous diffusion pathways, interfacial densification, and reduced segmental mobility of the polymer matrix. The term “hybrid” is used here in a broader sense than “composite”, encompassing both classical matrix–filler systems and multifunctional material combinations designed to achieve synergistic barrier properties.

**Figure 11:**
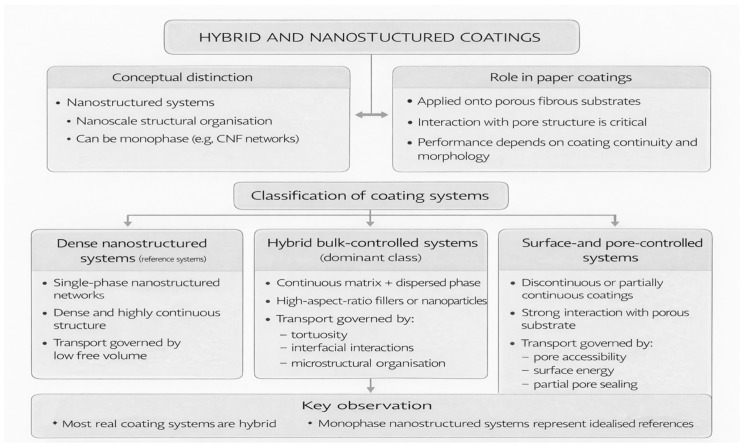
**Classification of hybrid and nanostructured coating systems for paper-based substrates.** The scheme distinguishes nanostructured systems, hybrid bulk-controlled systems, and surface- and pore-controlled systems according to the dominant transport-control mechanism.

From a mechanistic perspective, transport phenomena in hybrid and nanostructured coatings are controlled through microstructural architecture rather than through a single dominant property. The main mechanisms involved include: (i) the formation of dense and continuous nanostructured networks with reduced free volume, (ii) the introduction of tortuous diffusion pathways through high-aspect-ratio fillers, (iii) interfacial densification and restriction of polymer chain mobility induced by nanoparticle–matrix interactions, and (iv) pore sealing and surface-energy modification, which reduce wettability and limit capillary penetration into the fibrous substrate. Although these mechanisms are often described separately, in practical systems they frequently coexist, and their individual contributions are difficult to isolate. A schematic overview of the classification is provided in Figure 11.

The three classes identified in Figure 11 differ in the level at which transport control occurs, namely nanostructured monophase systems, hybrid bulk-controlled systems, and surface- and pore-controlled systems. Nanostructured monophase systems are characterised by the absence of a distinct dispersed phase despite being structurally heterogeneous at the nanoscale, with barrier performance arising from the intrinsic packing and continuity of the coating. Nanocellulose-based coatings represent the most relevant example of this class, where highly dense hydrogen-bonded networks significantly reduce gas permeability through diffusion-controlled mechanisms. In these systems, barrier performance is primarily governed by network density, crystallinity, and coating uniformity.

Hybrid systems with bulk-controlled transport include all coatings in which a continuous matrix is combined with a dispersed phase, and where the dominant barrier mechanisms act within the bulk of the coating layer. In these systems, the presence of fillers modifies the effective diffusion pathways and the microstructure of the matrix. High-aspect-ratio fillers such as nanoclays generate tortuous diffusion pathways, increasing the effective transport distance of permeating molecules, while nanoparticle-based systems, including lignin nanoparticles, nanocellulose and inorganic fillers, contribute to interfacial densification and reduction of free volume. In many cases, these mechanisms act simultaneously, resulting in combined effects that depend on filler morphology, dispersion quality, and matrix–filler compatibility. As a result, hybrid systems can achieve significantly improved barrier performance compared to single-phase coatings, although their effectiveness remains strongly dependent on formulation and processing conditions.

Surface- and pore-controlled systems represent a distinct class in which barrier performance is governed primarily by interfacial phenomena rather than by transport through a continuous film. In these systems, coatings act by modifying surface energy, reducing wettability, and partially sealing the porous structure of the paper substrate. This behaviour is typically observed in systems incorporating particulate fillers or hydrophobic components that do not form fully continuous films or form discontinuous or partially percolated structures. As a result, these coatings are particularly effective in reducing liquid water uptake and improving resistance to oils and greases, while their gas barrier performance remains limited compared to dense or hybrid bulk-controlled systems.

**Figure 12:**
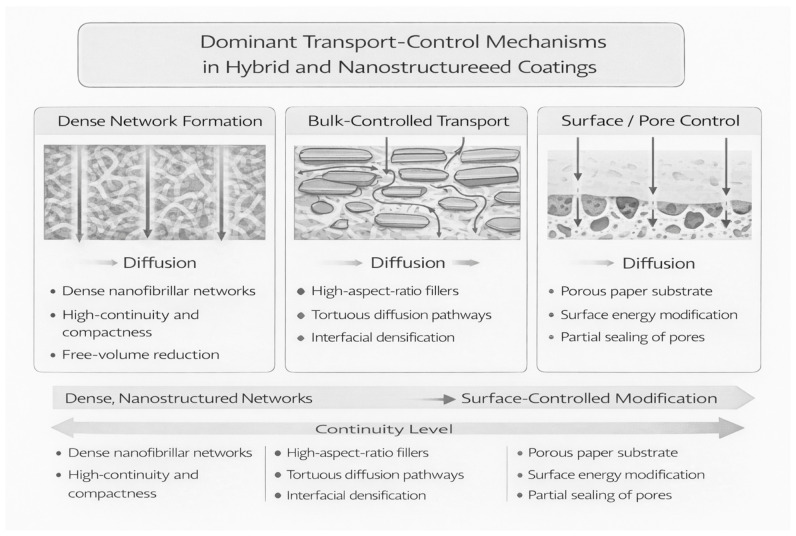
**Dominant transport-control mechanisms in hybrid and nanostructured coatings.** The scheme illustrates dense nanostructured networks, hybrid bulk-controlled systems, and surface- and pore-controlled systems, highlighting how microstructural features modify diffusion pathways and barrier behaviour.

The different transport-control regimes described above are schematically illustrated in Figure 12, which provides a unified representation of the transition between surface-controlled and diffusion-controlled regimes. Unlike purely material-based classifications, this framework highlights the central role of microstructural organisation in governing barrier performance across a wide range of coating systems.

Overall, hybrid and nanostructured coatings demonstrate that barrier enhancement can be achieved through multiple often synergistic mechanisms, including structural densification, tortuous diffusion, interfacial interactions, and surface modification. Performance ultimately depends on microstructure, filler dispersion, and coating architecture, rather than on any single design parameter.

#### 2.6.1. Nanostructured Monophase Systems (Dense Network Formation)

Nanostructured monophase systems represent the most direct expression of diffusion-controlled transport in paper coatings, where barrier performance arises from the intrinsic organisation of a continuous phase rather than from multiphase interactions. In these systems, the absence of a distinct dispersed phase implies that transport phenomena are governed primarily by network density, structural continuity, and free volume reduction.

A representative example is provided by nanocellulose-based coatings [118], in which highly entangled nanofibrillar networks form dense and cohesive films capable of significantly reducing pore size and limiting gas diffusion. This behaviour is consistent with the general mechanism reported for nanocellulose-based systems, where the formation of highly interconnected fibrillar networks leads to low porosity and diffusion-controlled transport [119,120]. The high aspect ratio of cellulose nanofibrils favours the development of percolating structures, effectively reducing pore connectivity and promoting the formation of a more continuous and compact barrier layer. As a result, substantial improvements in air and grease resistance are observed, with Gurley values exceeding 10^5^ s and Cobb values reduced to approximately 50–100 g/m^2^.

Despite these advantages, the barrier performance of nanostructured monophase systems remains intrinsically limited by their hydrophilic nature. Water uptake induces swelling and plasticisation of the network, increasing chain mobility and free volume, and thereby reducing gas barrier performance. This moisture sensitivity is widely reported for nanocellulose-based systems [121,122]. These systems therefore represent a fundamental reference for diffusion-controlled transport, although practical applications typically require additional modification or integration into more complex hybrid or multilayer architectures. From an application perspective, these systems are particularly relevant for dry food packaging, where high oxygen and grease barrier performance can be achieved under controlled humidity conditions, although their effectiveness rapidly decreases at high relative humidity.

#### 2.6.2. Hybrid Systems with Bulk-Controlled Transport

Hybrid systems with bulk-controlled transport constitute one of the most representative classes of advanced coatings, as they enable the simultaneous activation of multiple transport-control mechanisms through the interaction between a continuous matrix and a dispersed phase. In these systems, barrier performance is governed by the internal microstructure of the coating layer, where filler morphology, dispersion quality, matrix–filler interactions, and coating continuity determine the effective diffusion pathways.

A first group includes systems in which tortuous diffusion contributes to barrier performance, typically through the incorporation of high-aspect-ratio or layered fillers. In these systems, anisotropic nanostructures can increase the effective diffusion path length of permeating molecules, thereby reducing permeability when properly dispersed within the coating matrix. Representative examples include CNF–nanoclay hybrid coatings [25], PVA–bentonite systems [106], crosslinked PVA–nanoclay coatings [105], and PVA/AKD/nanoclay coatings [36], where barrier enhancement arises from the combined effect of continuous film formation, pore sealing, and nanoclay-induced tortuous diffusion pathways. In particular, Shen et al. (2021) [36] reported a decrease in WVTR from approximately 533 to 1.3 g m^−2^ day^−1^ after double coating, corresponding to a reduction of about 99.8%, associated with complete surface coverage by the coating layer. This behaviour is consistent with broader analyses reported in the literature, where nanoclay-based nanocomposites are described as layered systems that, when properly intercalated or exfoliated, generate tortuous diffusion pathways that hinder molecular transport [72].

A second group includes hybrid systems in which barrier enhancement is governed primarily by interfacial densification, reduced free volume, and coating continuity, rather than by geometric tortuosity alone. Lignin nanoparticle-based coatings [123] exemplify this behaviour. In these systems, lignin nanoparticles interact with the cationic starch matrix through hydrogen bonding, promoting the formation of a dense and continuous coating layer on the paper surface. This structure improves water resistance, oil resistance, tensile strength, and water vapour barrier performance, with WVTR decreasing from approximately 2569 to 426 g m^−2^ day^−1^, corresponding to a reduction of about 83%. Comparable mixed-mechanism behaviour is reported in polymer nanocomposite coatings incorporating cellulose nanocrystals or blended matrices, such as PLA/PMMA/m-CNC coatings [124], where barrier performance depends on filler dispersion, polymer–filler compatibility, coating grammage, and microstructural continuity. These trends reflect the broader role of interfacial interactions, filler dispersion, and structural organisation in hybrid nanostructured systems [119,125] and are consistent with recent reviews highlighting the effectiveness of nanocellulose-based fillers in enhancing both barrier and mechanical properties [122].

A third subgroup includes network-controlled hybrid systems, which differ from interfacial-densification systems in that barrier properties are governed by the formation of continuous, interconnected polymeric networks extending throughout the coating layer. In these systems, dense structural organisation and network continuity reduce porosity and limit mass transport. Polysaccharide-based coatings such as Sc–CL–CNF systems [47] illustrate this behaviour, where hydrogen bonding and electrostatic interactions between the components promote the formation of cohesive and continuous coating layers, contributing to improved mechanical and barrier performance. A similar behaviour is observed in starch–nanocrystal systems [126], where the dispersion of cellulose nanocrystals within the polymer matrix enhances matrix cohesion and promotes pore filling, leading to significant reductions in water absorption and improvements in air and oil resistance. In these systems, transport control is primarily associated with structural continuity, pore filling, and reduced free volume, rather than being dominated exclusively by tortuous diffusion pathways.

Overall, hybrid bulk-controlled systems highlight the interplay between tortuosity, interfacial interactions, and structural organisation. The relative contribution of each mechanism depends strongly on filler morphology, dispersion quality, and processing conditions, while excessive filler loading or structural complexity may lead to aggregation, defects, or reduced processability.

#### 2.6.3. Surface- and Pore-Controlled Systems

Surface- and pore-controlled systems represent a distinct class of coatings in which barrier performance is governed primarily by interfacial phenomena rather than by transport through a continuous film. In these systems, coatings may not form fully dense diffusion barriers across the entire thickness, but instead act by modifying surface energy and partially sealing the porous structure of the substrate.

A representative example is provided by hydrophobic polymer–mineral systems such as ethyl cellulose combined with bio-derived CaCO_3_ [127]. In these coatings, barrier performance is achieved through a combination of pore blocking and wettability reduction, which significantly limits liquid water penetration.

This behaviour is typical of systems in which surface hydrophobisation and partial pore sealing dominate transport control, resulting in strong resistance to liquid uptake but are generally less effective for gas barrier applications.

However, due to the absence of a continuous and dense diffusion barrier, gas permeability remains relatively high compared to bulk-controlled hybrid systems. Therefore, while surface- and pore-controlled coatings are particularly suitable for applications requiring liquid resistance, they are less effective in applications demanding high gas barrier performance. For this reason, these systems are often combined with additional coating layers in multilayer architectures when both liquid resistance and gas barrier performance are required.

The systems reported in Table 9 show that transport control in paper coatings evolves from intrinsic material behaviour to increasingly complex microstructural architectures. Nanostructured monophase systems provide a useful reference for understanding diffusion-controlled transport, but practical applications are largely dominated by hybrid bulk-controlled coatings, in which tortuous diffusion pathways, interfacial densification, and network continuity often operate simultaneously. Surface- and pore-controlled systems, although simpler in design, remain relevant for applications primarily requiring liquid water resistance and surface hydrophobisation.

Overall, barrier enhancement depends on the combined influence of microstructure, interfacial interactions, and coating architecture. Rather than depending on a single dominant mechanism, effective coating design requires the combination of complementary transport-control strategies tailored to specific barrier targets and processing constraints.

### 2.7. Transition to Multilayer, Extrusion-Coated and Laminated Architectures

Paper-based coating systems discussed in Section 2.1, Section 2.2, Section 2.3, Section 2.4, Section 2.5 and Section 2.6 highlight the intrinsic limitations of monolayer approaches, which are often unable to simultaneously satisfy all key performance requirements. These limitations arise from the conflicting behaviour of individual material classes and cannot be fully addressed through single-component optimisation.

As schematically illustrated in Figure 5 (Panel 5), multilayer architectures represent an advanced level of transport control, where barrier performance emerges from the spatial separation and integration of complementary functions. In this framework, different materials are combined to exploit distinct transport-control mechanisms within a single system.

This transition encompasses multilayer coating strategies, extrusion-coated configurations, and laminated systems, which represent complementary routes for achieving integrated performance. In multilayer and extrusion-coated systems, functional layers are formed directly on the paper substrate, whereas in laminated structures, pre-formed films or foils are coupled to the paper through adhesive bonding or thermal lamination. Despite these differences, all these approaches rely on the combination of materials with distinct properties to achieve improved barrier behaviour, mechanical integrity, and processability.

In such architectures, performance emerges from the interaction between layers and interfaces, rather than from the intrinsic properties of individual materials. Defects such as microcracking, delamination, or incomplete bonding may generate preferential pathways for gas and moisture transport, significantly affecting barrier performance under real conditions.

From a design perspective, this evolution represents a shift from material-driven to architecture-driven design, where performance depends on the interaction between layers, interfaces, and processing conditions. However, increasing structural complexity introduces constraints related to processability, recyclability, and regulatory compliance.

The structure, processing, and performance of multilayer, extrusion-coated, and laminated systems are analysed in detail in Section 3. Before considering these more complex architectures, it is important to recognise that no coating technology can simultaneously maximise barrier performance, mechanical robustness, processability, recyclability, and end-of-life compatibility. Polysaccharide-based coatings generally offer favourable renewable sourcing and fiber recovery but often remain sensitive to moisture. Polymeric coatings provide more stable barrier and sealing performance, whereas hybrid and nanostructured systems can achieve superior functionality through microstructural control at the expense of increased formulation and processing complexity. Consequently, coating selection should be guided by the specific balance required between functional performance, industrial applicability, and circularity objectives.

## 3. Multilayer Architectures in Paper-Based Packaging

The transition from single-layer coatings to multilayer architectures represents a key step in the evolution of paper-based packaging systems, enabling the integration of complementary functional properties within structurally engineered configurations. The limitations of biodegradable and bio-based materials, particularly in terms of oxygen and water vapour barrier performance, have been widely documented, highlighting the need for combined strategies capable of overcoming the intrinsic trade-offs between gas barrier, moisture resistance, and mechanical integrity [14].

In this context, multilayer design has emerged as a fundamental approach, in which materials with distinct transport and mechanical characteristics are combined to achieve balanced performance [13,129]. Typically, hydrophilic or polar layers provide effective oxygen barrier properties, while hydrophobic or non-polar layers improve resistance to water vapour and enhance mechanical stability. This functional complementarity, widely adopted in both conventional and emerging systems, enables performance levels that cannot be achieved by single-material coatings alone [130].

From a structural perspective, multilayer packaging relies on the spatial separation of functions across the thickness of the material, where inner sealant layers, intermediate barrier layers, and outer structural layers contribute to the overall performance of the system [131].

As a result, barrier behaviour is governed not only by the intrinsic properties of individual materials, but also by interfacial adhesion, layer continuity, and defect control, which determine the effective transport pathways through the structure.

However, the increasing structural complexity associated with multilayer architectures introduces significant challenges in terms of end-of-life management. The combination of multiple materials, often with different chemical and physical properties, complicates recycling processes and limits material recovery efficiency, highlighting a fundamental trade-off between barrier performance and circularity [132].

Building on these considerations, multilayer architectures in paper-based packaging can be broadly classified into three main categories: (i) bio-based multilayer coating systems, (ii) polymer–paper multilayer and extrusion-coated systems, and (iii) laminated and high-performance multilayer structures.

These systems differ not only in composition but also in structural organisation, interfacial behaviour, and response to industrial processing operations such as folding, sealing, and thermoforming.

Table 10 provides a comparative framework of these architecture classes, highlighting the trade-offs between barrier performance, mechanical behaviour, processability, and end-of-life constraints. Detailed case studies are reported in Table S1 (Supplementary Materials), which illustrates the variability of multilayer configurations and materials within each class.

**Figure 13:**
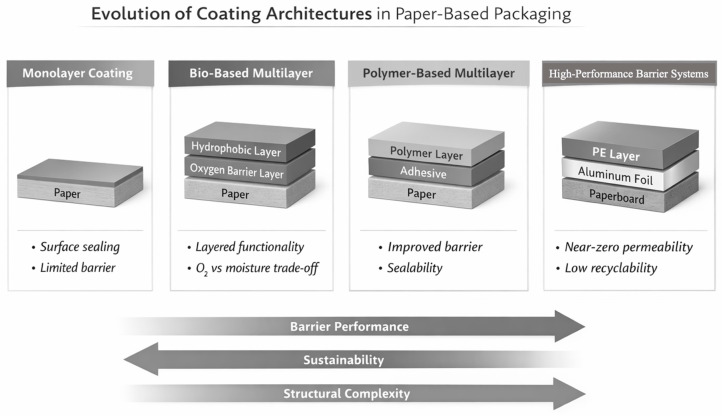
**Evolution of coating architectures in paper-based packaging, from monolayer coatings to high-performance multilayer systems.** The progression highlights the increasing use of structural design to combine complementary barrier functions, transitioning from bio-based multilayer coatings to polymer-based systems and finally to high-performance barrier architectures, including aluminum-based laminates, metallized coatings, and inorganic thin layers. This evolution is associated with improved barrier performance but increased structural complexity and reduced recyclability.

The structural progression from surface coatings to integrated multilayer architectures is schematically illustrated in Figure 13, which highlights the increasing role of interfacial design and functional integration in determining overall performance. This progression reflects the transition from material-driven approaches to architecture-driven design, where performance is governed by the interaction between layers rather than by the intrinsic properties of individual materials.

The following subsections examine the main classes of multilayer systems developed on paper-based substrates, with particular attention to how structural design influences barrier behaviour, mechanical response, and end-of-life compatibility.

### 3.1. Bio-Based Multilayer Coating Systems

Bio-based multilayer coatings represent the first level of structural design in paper-based barrier systems, where performance arises from the spatial organisation of multiple layers and their interfacial interactions.

These systems do not constitute a homogeneous category, but include configurations with different degrees of architectural complexity. Based on layer function, deposition strategy and structural organisation, they can be divided into three main groups: functionally stratified multilayer systems, simple bilayer or multilayer coatings, and sequential or layer-by-layer assemblies [7,34,133]. The systems reported in Table S1 span a continuum from sequential coatings, in which performance evolves progressively through repeated deposition and microstructural densification, to functionally stratified multilayer architectures, in which distinct layers perform complementary roles such as oxygen barrier, moisture protection, adhesion or heat sealability.

#### 3.1.1. Functionally Stratified Multilayer Systems

In functionally stratified multilayer coatings, individual layers are designed to fulfil specific roles, such as oxygen barrier, moisture protection, or interfacial adhesion. This approach enables a partial decoupling of transport properties and represents the closest analogue to conventional multilayer laminates.

This behaviour is observed in hybrid and multi-component systems such as the thermoplastic starch (TPS)-based multilayer structures reported by Eslami et al. [27], where a hydrophilic starch layer contributes to oxygen barrier while external biodegradable polymer layers improve moisture resistance and mechanical performance. A similar layered diffusion control mechanism is observed in potato fruit juice (PFJ)–PHB systems [134], where the interlayer enhances oxygen barrier and adhesion, while the PHB coating provides moisture protection.

More complex architectures are represented by hybrid inorganic–organic systems such as PLAX/bioORMOCER^®^/PLAX coatings [135], where barrier performance arises from the combination of PLAX protective layers and a hybrid inorganic–organic barrier layer. A further level of structural hierarchy is achieved in fully engineered multilayer systems such as those reported by Ma et al. [136], where distinct layers contribute to pore sealing, tortuous diffusion pathways, hydrophobic barrier formation, and defect suppression. In these systems, performance is governed by the synergistic interaction between layers rather than by the properties of a single phase.

#### 3.1.2. Simple Multilayer and Bilayer Coatings

A second group includes systems in which multiple layers are present, but with a lower degree of functional differentiation compared to fully stratified architectures. In these cases, multilayer behaviour arises from the combination of materials with partially overlapping roles rather than from a strict allocation of functions.

Typical examples include CNC/chitosan (Cht) bilayer coatings [137], where a dense nanocellulose layer provides structural barrier properties, while a chitosan top layer enhances surface sealing and moisture resistance. Similarly, CNF/PLA–CNF/cocoa butter (CB) systems [138] combine film-forming and hydrophobic phases in a sequential configuration, resulting in improved water vapour resistance and surface properties.

Protein–polymer bilayers such as PVOH–zein coatings [139] also belong to this category, where barrier improvement is associated with pore sealing and surface hydrophobization rather than with fully separated transport functions.

#### 3.1.3. Sequential Multilayer and Layer-by-Layer Coatings

A third group includes systems in which the multilayer character derives from repeated deposition rather than from a predefined architecture. In these coatings, the first layers interact with and partially penetrate the porous structure of paper, while subsequent layers progressively consolidate the surface, leading to the formation of a continuous film.

This behaviour is clearly observed in chitosan multilayer coatings [140], where barrier and mechanical properties improve significantly only after multiple deposition cycles, reflecting the progressive transition from a porous to a film-like structure. A similar mechanism is observed in cellulose-based multilayer systems such as methylcellulose (MeNC), microfibrillated cellulose (MFC), and hydrophobically modified ethyl hydroxyethyl cellulose (HM-EHEC) coatings [141], where barrier performance is governed by hydrogen-bonded networks and progressive pore closure, but remains strongly affected by humidity due to the hydrophilic nature of the materials.

### 3.2. Polymer–Paper Multilayer Systems

Polymer–paper multilayer systems represent a transition from coating-based approaches to fully engineered packaging architectures, in which a continuous polymer phase is integrated with a fibrous substrate to achieve enhanced barrier and mechanical performance. In industrial practice, multilayer and laminated structures combining paperboard with polyethylene, aluminum foil, and polymer films are widely employed to provide simultaneous protection against oxygen, moisture, light, and contaminants, particularly in liquid food and dairy packaging applications [142]. The effectiveness of these systems arises from the integration of materials with complementary functions within a single structure, enabling performance levels beyond those attainable with monolayer configurations [27,131,134,143,144].

In these architectures, thermoplastic polymers such as PHB, PHBV, poly(butylene adipate-co-terephthalate) (PBAT), PLA, or starch-based blends are applied through extrusion coating, lamination, or compression techniques to form continuous films on the paper surface. From a structural perspective, performance primarily depends on the formation of a dense polymer layer that reduces surface porosity and stabilises transport pathways, coupled with the mechanical support provided by the fibrous substrate. Compared with fully bio-based multilayer coatings, these systems generally exhibit improved resistance to moisture and grease, as well as enhanced sealability, flexibility, and robustness under converting conditions.

The behaviour of the polymer phase plays a critical role in determining overall performance. Aliphatic polyesters such as PBAT are associated with higher flexibility and improved sealing behaviour, making them suitable for applications requiring mechanical compliance. By contrast, PHB and PHBV offer more favourable gas barrier performance but are limited by brittleness, narrow processing windows, and a tendency to develop defects under deformation [143,144]. PLA-based and starch-containing systems typically occupy an intermediate position, often requiring blending or multilayer combinations to balance stiffness, moisture resistance, and processability.

A key aspect of these systems is the functional separation across the thickness of the structure. The paper substrate provides stiffness, dimensional stability, and printability, while the polymer layer acts as a continuous barrier phase and enables heat sealing. As a result, performance depends strongly on coating continuity, layer thickness, and interfacial adhesion. Insufficient adhesion may lead to delamination or microdefect formation, which in turn compromises barrier integrity and mechanical reliability under processing and service conditions.

Representative systems confirm these trends. PHB-coated paperboard structures exhibit significant reductions in water vapour transmission rate, together with improved interfacial adhesion [134]. PHBV-based multilayer configurations provide enhanced mechanical resistance and barrier properties, although with limitations related to rigidity and reduced flexibility [143]. PBAT-containing multilayers are more effective in ensuring sealing performance and mechanical compliance, particularly under deformation [144]. TPS-based multilayer assemblies combined with additional biodegradable polymers demonstrate improved moisture barrier, grease resistance, and wet mechanical performance while maintaining a partially bio-based character [27].

Polymer–paper multilayers bridge the gap between coating-based systems and high-performance industrial laminates. Their improved structural stability and converting compatibility are achieved at the cost of increased material complexity, which complicates recycling and often limits end-of-life options to energy recovery or downcycling pathways (Kaiser, 2017; Eissenberger, 2023) [131,133].

Representative examples of polymer–paper multilayer systems are summarised in Table 10.

### 3.3. Laminated and High-Performance Multilayer Systems

High-performance multilayer systems represent the industrial benchmark for barrier functionality in paper-based packaging, enabling near-complete protection against gases, moisture, light, and volatile compounds through the integration of dedicated barrier layers. Unlike the bio-based multilayer coatings discussed in Section 3.1 and the polymer–paper multilayer systems described in Section 3.2, these architectures are explicitly engineered to maximise barrier performance and shelf life through the spatial separation of functions across distinct material layers.

Within this class, laminated architectures constitute a specific configuration in which pre-formed films or foils are coupled to paper substrates through adhesive bonding or thermal lamination. These systems include ethylene vinyl alcohol copolymer (EVOH)-based multilayers, metallized structures, inorganic-coated films, and aluminum-containing laminates, each providing targeted barrier performance depending on the application.

Among these, EVOH-based multilayer systems play a central role as high-performance oxygen barrier solutions. EVOH exhibits extremely low gas permeability due to its polar structure and dense molecular packing, making it one of the most effective polymeric barriers currently available [145]. For this reason, it is typically integrated within multilayer structures such as paper/PE/EVOH/PE, where it functions as the primary gas barrier layer. However, its performance depends on environmental conditions, and therefore it is generally protected by external hydrophobic layers to ensure stability under service conditions [145].

Aluminum-based laminates represent the upper limit of barrier performance in industrial packaging systems. These multilayer structures, typically composed of paperboard, polyethylene, and aluminum foil, combine mechanical support with nearly complete barrier functionality. The paper layer provides stiffness and structural integrity, the polymer layers enable sealing and interfacial adhesion, and the aluminum foil acts as an effective barrier against oxygen, light, moisture, and volatile compounds [146]. In typical beverage carton systems, the composition is approximately 75% paperboard, 20% polyethylene, and 5% aluminum, reflecting an optimized balance between structural performance and barrier efficiency [147].

Alternative high-barrier strategies have been developed to reduce material usage and improve flexibility. Metallized systems, based on vacuum-deposited aluminum layers, provide oxygen barrier properties with reduced thickness, although their performance may be affected by defects such as pinholes and coating discontinuities, as commonly reported for metallized barrier layers [9]. Similarly, inorganic coatings such as silicon oxides (SiOx) and aluminum oxides (AlOx) offer thin, transparent barrier layers with low gas permeability, but their effectiveness depends on coating integrity and resistance to mechanical damage.

Across all these architectures, overall behavior reflects a clear functional differentiation of layers: the paper substrate provides mechanical stability, polymer layers ensure processability and sealing, and metallic or inorganic layers deliver barrier performance. This design strategy reflects a fully engineered multilayer approach, in which barrier properties are no longer an emergent effect of coating structure, but the result of deliberate material integration.

Despite their superior performance, these systems present significant challenges in terms of end-of-life management. Their multi-material nature complicates recycling processes, as efficient recovery requires the separation of strongly bonded and often incompatible layers. As a result, high-performance multilayer systems highlight a fundamental trade-off between maximum barrier functionality, structural complexity, and circularity [131,133].

Nanostructured and hybrid features should therefore be interpreted as cross-cutting design strategies that can be incorporated across bio-based coatings, polymer–paper multilayers, and high-performance laminated systems. From an industrial and recycling-oriented perspective, these architectures cannot be evaluated solely on the basis of barrier efficiency. EVOH-based, metallized, inorganic-coated, and aluminium-containing systems provide different balances between protection level, defect sensitivity, layer separability, fiber recovery, and compatibility with conventional recycling streams. Consequently, multilayer architectures generally provide higher barrier and functional performance than coated paper systems, but these gains are achieved at the expense of increased structural complexity and more demanding end-of-life management.

## 4. Design Trade-Offs in Paper-Based Packaging Systems

The evolution of coating and multilayer strategies for paper-based packaging highlights a fundamental trade-off between barrier performance, structural complexity, circularity, and converting compatibility, consistently reported across recent studies on sustainable paper-based packaging systems. This trade-off arises from the need to combine materials with complementary properties in order to overcome the intrinsic limitations of cellulose-based substrates, often resulting in multi-material systems that are more difficult to recycle [9,27,131].

As discussed in Section 2 and Section 3, different material systems and design strategies address these limitations through distinct mechanisms, including dense film formation, surface hydrophobisation, microstructural control, and multilayer functional decoupling. Each of these approaches enables specific performance improvements but also introduces corresponding limitations related to mechanical behaviour, processability, and end-of-life compatibility.

In this context, barrier performance cannot be interpreted as an intrinsic property of a given material class, but rather as the result of the interaction between material composition, microstructural organisation, and architectural design. Strategies based on dense hydrophilic networks, such as polysaccharide and protein coatings, provide excellent oxygen barrier properties under dry conditions but are highly sensitive to moisture. Conversely, hydrophobic systems such as lipid-based coatings effectively reduce water permeability while offering limited resistance to gas transport.

More advanced solutions, including nanostructured coatings and hybrid systems, exploit microstructural design to improve barrier performance through tortuous diffusion pathways and enhanced interfacial interactions, although their effectiveness depends strongly on dispersion quality and structural continuity. Multilayer and laminated architectures represent the most effective approach for achieving high overall barrier performance, as they enable the spatial separation and integration of complementary functions. However, this increased performance is accompanied by higher structural complexity, greater sensitivity to interfacial defects, and reduced circularity due to the presence of multiple material components.

Overall, these considerations highlight that no single design strategy can simultaneously optimise all performance criteria. Instead, effective packaging design requires the selection and integration of material systems according to specific application requirements, balancing barrier performance, mechanical integrity, converting compatibility, and circularity constraints. In this perspective, the systems analysed in Section 2 and Section 3 should be interpreted as complementary solutions within a broader design space, rather than as directly comparable alternatives.

The comparative trends discussed in this section should therefore be interpreted within the limitations of the available literature, since reported values are often influenced by differences in substrate characteristics, coating thickness, processing conditions, relative humidity, and testing methodology. Consequently, the analysis aims to identify recurring structure–performance relationships rather than establish direct quantitative comparisons among materials.

### 4.1. Evaluation Criteria for Functional Performance

The analysis presented in Table 11 is based on a set of key performance dimensions selected to capture the main functional and industrial constraints governing the design of paper-based packaging systems. These include oxygen barrier performance, water vapour resistance, mechanical behaviour, converting compatibility, circularity, and structural complexity.

Barrier performance is distinguished into oxygen barrier and water vapour barrier in order to reflect the fundamentally different transport mechanisms governing gas and moisture permeation. Mechanical behaviour describes the stiffness, flexibility, and structural integrity of the system, which are critical for both handling and end-use performance. Converting compatibility refers to the processability of the system under industrial conditions, including sealing behaviour, flexibility, and integration within existing packaging lines. Circularity is evaluated in terms of compatibility with recycling, composting, or material recovery pathways, taking into account the effects of coatings, additives, and multi-material configurations.

Structural complexity reflects the degree of material heterogeneity, interfacial organisation, and architectural design, including the presence of multiple layers, dispersed phases, or functional interfaces. This parameter is particularly relevant in understanding the trade-offs between performance enhancement and end-of-life management, as well as the susceptibility of systems to defects such as delamination or microcracking.

The qualitative levels reported in Table 11 (e.g., low, moderate, high) are derived from the performance trends observed in the literature discussed in Section 2 and Section 3, including the analysis of representative experimental data. These levels do not correspond to averaged or normalized values, but rather to typical functional behaviour under relevant conditions. Accordingly, the table is intended to provide a comparative framework for interpreting the capabilities and limitations of different design strategies, rather than a direct quantitative ranking of systems.

### 4.2. Mechanical Performance, Barrier Integrity and Converting Compatibility

The performance of coated and multilayer paper-based packaging systems cannot be evaluated solely in terms of intrinsic material properties, but must also be interpreted in relation to their behaviour under real processing and application conditions, as widely recognised in packaging and adhesion science [2,148].

In this framework, functional behaviour arises from the combined effects of interfacial adhesion, layer sequence, coating quality, and microstructural organisation, which together influence mechanical response, barrier performance, and convertibility [27,115]. Improvements in barrier properties are often associated with changes in mechanical behaviour and converting performance, while increasing material and structural complexity may introduce additional challenges for recyclability and end-of-life management [2,9].

Building on the functional capabilities and design trade-offs summarised in Table 11, the following analysis provides an engineering-level interpretation of how different material systems and design strategies translate into mechanical behaviour, barrier integrity, interfacial stability, and converting response under real operating conditions. In particular, systems based on bio-based coatings often exhibit strong oxygen barrier performance but limited moisture resistance [32,44,149], whereas multilayer configurations improve barrier properties through layer combination and structural design [27,134,143]. High-barrier laminates represent the upper performance benchmark due to the presence of dense or metallic layers enabling near-zero permeability.

**Figure 14:**
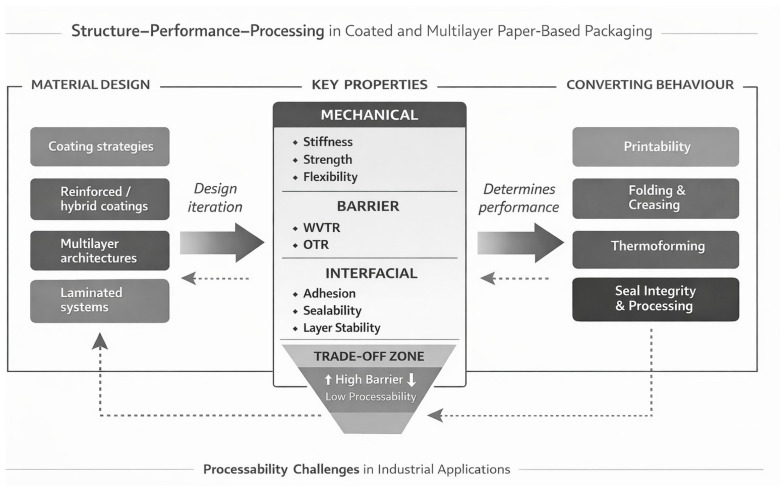
**Structure–performance–processing relationships in coated and multilayer paper-based packaging systems.** Material design strategies determine key functional properties, including mechanical behaviour, barrier performance, and interfacial characteristics, which in turn govern converting behaviour such as printability, folding, thermoforming, and sealing. The figure highlights the trade-offs between barrier performance, processability, and structural complexity, emphasising the need for integrated design approaches.

These considerations highlight the central role of interfacial design, coating continuity, and layer architecture in determining the overall performance of paper-based packaging systems. The corresponding structure–performance–processing relationships are schematically summarised in Figure 14. On this basis, the following sections analyse paper-based packaging systems through four complementary perspectives—mechanical response, mass transport behaviour, interfacial phenomena, and converting compatibility—allowing a consistent interpretation of how structural design translates into performance under real operating conditions. These aspects are strongly interconnected and governed by common structure–property–process relationships.

#### 4.2.1. Mechanical Reinforcement and Structural Integrity

Mechanical performance is a fundamental requirement for paper-based packaging systems because it governs load-bearing capacity, dimensional stability, and resistance to damage during processing, handling, and use. In the present review, mechanical behaviour is interpreted within the structure-based framework developed in Section 2 and Section 3, progressing from fiber-dominated substrates to multilayer and laminated architectures where performance increasingly depends on interfacial adhesion, layer continuity, and structural integration.

**Figure 15:**
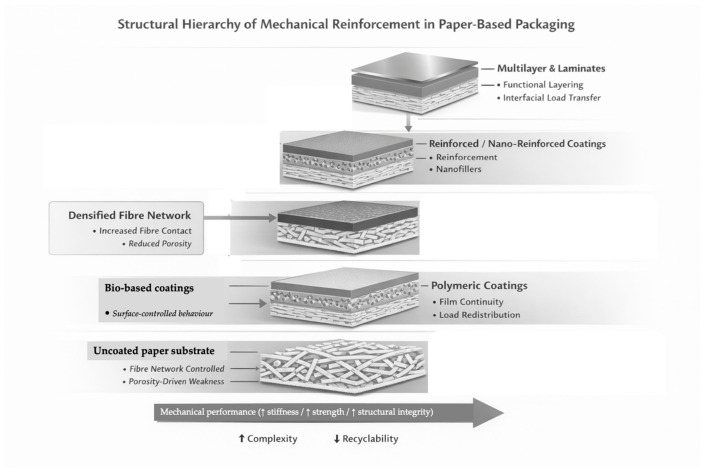
**Structural hierarchy of mechanical reinforcement in paper-based packaging systems.** The figure illustrates the transition from fiber-dominated behaviour in uncoated paper substrates to structurally integrated multilayer and laminated systems. Mechanical performance increases with structural complexity through improved stress transfer, interfacial interactions, and layer integration, while recyclability decreases due to multi-material configurations.

This structural hierarchy and the corresponding reinforcement mechanisms are schematically illustrated in Figure 15.

Direct quantitative comparison is not attempted because of differences in testing conditions, structural configurations, and reporting metrics. Nevertheless, representative mechanical data reported in the coating- and multilayer-specific tables provide the experimental basis for the structure–property relationships analysed in the present section.

At the lowest level of structural complexity, mechanical behaviour is primarily governed by the intrinsic properties of the paper substrate. Strength and stiffness originate from the fiber network, while porosity, structural heterogeneity, and moisture sensitivity represent the main limiting factors. Classical fiber bonding theories indicate that mechanical performance is controlled by the number and strength of inter-fiber bonds and by the effective contact area between fibers, which is strongly influenced by refining and densification processes [150,151]. In fiber-based substrates, tensile failure is governed by progressive bond breakage and damage localisation within the network, rather than by the failure of individual fibers. As a result, uncoated paper systems typically exhibit moderate stiffness and limited tensile strength, defining the baseline mechanical performance against which all subsequent structural modifications can be compared.

A first effective reinforcement pathway emerges when the strategy promotes fiber network densification or fibrillar consolidation within the paper structure itself. Systems based on cellulose filaments or refined fibrillar structures exhibit increased tensile strength and stiffness due to improved fiber–fiber bonding, reduced porosity, and enhanced stress transfer within the network. This behaviour has been clearly demonstrated in engineered cellulose-based papers, where increasing the filament fraction leads to significant improvements in tensile index and structural cohesion [152]. This approach directly modifies the bulk fibrous structure and therefore produces intrinsic improvements in load-bearing capacity and stiffness.

Once the behaviour of the substrate and its bulk-modified variants is defined, further improvements can be achieved through surface engineering strategies, primarily based on coating technologies. In these systems, the fibrous network remains the main load-bearing component, while the coating layer modifies surface properties, defect sensitivity, and interfacial stress transfer. At the simplest level, mono-component bio-based coatings provide limited mechanical reinforcement. Their primary function is surface sealing and partial consolidation, which can reduce defect initiation and improve surface cohesion, but without significantly altering the internal fiber network. Consequently, improvements in tensile strength and stiffness are typically modest, and the overall mechanical performance remains close to that of the uncoated substrate [34,153]. Polymeric coatings represent a further step in surface-based reinforcement. Due to their ability to form continuous and more homogeneous films, these coatings improve mechanical continuity at the surface and contribute to local stress redistribution. In particular, film-forming polymers such as PHBV, PLA, PVA, or PBAT can enhance ductility and reduce brittleness, especially under conditions where the fibrous substrate alone would exhibit limited deformation capability. However, even in these systems, the coating does not act as the primary load-bearing phase in most coating-dominated systems, and the overall mechanical response remains largely influenced by the substrate and by the quality of adhesion at the interface [134,143].

An intermediate level of complexity is represented by reinforced coatings and hybrid systems, in which micro- or sub-micrometric fillers (e.g., mineral particles such as CaCO_3_ or clay platelets, short cellulose fibers, starch granules, or microfibrillated cellulose aggregates) are incorporated into the coating matrix. In these systems, reinforcement arises from composite effects, improved film cohesion, and partial bridging of surface defects. The resulting mechanical improvements are more pronounced than in simple coatings, particularly in terms of resistance to crack initiation and propagation. However, in most cases, these effects remain primarily confined to the coating layer and the immediate interface, without substantially modifying the bulk load-bearing capacity of the fibrous network. The effectiveness of this reinforcement depends mainly on film continuity, filler–matrix interactions, and dispersion quality, while coating thickness alone is generally not sufficient to ensure significant mechanical contribution [154].

A further evolution is represented by nanoreinforced coatings, in which reinforcement is introduced at the nanoscale through elements such as cellulose nanofibers (CNF), cellulose nanocrystals (CNC), or mineral nanofillers. These systems exhibit a true nanostructured organisation, where the reinforcing phase forms an interconnected network that enhances stress transfer efficiency and mechanical continuity within the coating. This approach can be applied to both bio-based and polymeric matrices: in bio-based coatings, nanostructures compensate for limited film continuity by strengthening intermolecular interactions, whereas in polymeric coatings, they primarily increase stiffness and load transfer efficiency. Experimental evidence shows that nanostructured coatings lead to measurable increases in tensile strength and elastic modulus of the coated paper system, mainly due to improved interfacial bonding and more efficient stress redistribution [152,153,154]. Nevertheless, their reinforcing effect is primarily associated with the coating layer and the interface, and the overall load-bearing behaviour continues to be controlled by the substrate.

A further increase in mechanical performance is achieved in multilayer architectures, where different layers provide complementary mechanical functions. In these systems, the paper substrate provides stiffness and structural support, while additional layers contribute to mechanical continuity, flexibility, and stress redistribution. These layers are most commonly polymeric but can also consist of sequentially deposited bio-based components forming multilayer architectures. In such bio-based multilayer systems, the extent of mechanical reinforcement depends strongly on layer continuity, interfacial adhesion, and the ability to form cohesive films and is often more limited compared to fully continuous polymeric layers. As a result, the mechanical response is governed by load redistribution across layers and by interfacial adhesion.

Multilayer systems based on PHBV coatings have shown increased tensile strength and improved ductility compared to uncoated paper, confirming the role of continuous layers in enhancing structural integrity [143]. Within this class of systems, interfacial adhesion plays a decisive role. The introduction of intermediate layers or compatibilising strategies can significantly improve bonding between the paper substrate and coating layers, reducing delamination and enhancing stress transfer.

For instance, multilayer paperboard systems based on PHB and potato fruit juice (PFJ) exhibit improved cohesion and converting performance due to enhanced interfacial bonding [134]. At the highest level of structural complexity, industrial multilayer laminates combine paperboard, polymers, and, in some cases, metallic layers to achieve optimised performance. In beverage packaging laminates, paperboard provides stiffness and load-bearing capacity, polymer layers ensure flexibility and sealing, and aluminum primarily provides barrier performance while also contributing to bending stiffness due to its position within the laminate, even at very low thickness. However, its role in tensile load-bearing remains secondary due to its limited thickness and low deformability [146]. In these systems, mechanical optimisation is achieved through functional integration and through the spatial distribution of materials across the thickness rather than through the intrinsic performance of a single component.

Overall, mechanical behaviour follows a hierarchical progression from fiber-dominated substrates to fully integrated multilayer laminates. Increasing structural complexity generally improves strength, stiffness, and damage tolerance but is often accompanied by trade-offs in processability, recyclability, and flexibility. Mechanical design must therefore balance structural performance with system-level constraints.

#### 4.2.2. Barrier Performance and Transport Properties

Barrier performance in paper-based packaging systems is intrinsically limited by the porous and hydrophilic nature of the fibrous network. In uncoated paper substrates, mass transport occurs through an interconnected system of voids and fiber interfaces, enabling rapid permeation of gases and water vapour. As a result, oxygen and water vapour transmission rates remain high, and the material cannot provide adequate barrier performance for most packaging applications [36,96]. A first level of improvement can be achieved through fiber network densification and structural consolidation. By reducing porosity and increasing fiber–fiber contact, densified paper exhibits lower permeability compared to conventional substrates. However, this approach remains limited, as transport pathways are reduced but not eliminated, and diffusion through the fibrous structure continues to govern barrier behaviour. Consequently, improvements are moderate and insufficient for applications requiring strict permeability control [36,121].

A more effective transition occurs with the introduction of continuous coating layers, which represent the first shift from pore-controlled to diffusion-controlled transport. Film-forming materials, both synthetic and bio-based, seal surface pores and create a more compact phase that reduces permeability. In these systems, barrier performance is no longer governed solely by the substrate but increasingly depends on coating integrity and continuity. However, localized defects in the coating layer can significantly affect transport behaviour, leading to defect-controlled permeation that overrides the intrinsic barrier properties of the coating [36,96].

Further enhancement is achieved through nanostructured coatings, where fillers such as nanocellulose or nanoclays are incorporated into the matrix. These systems modify transport at the microscale by increasing the tortuosity of diffusion pathways, forcing permeating molecules to follow longer and more complex trajectories. This mechanism effectively reduces diffusivity, particularly for oxygen, without necessarily requiring substantial increases in coating thickness. The resulting barrier improvement strongly depends on filler dispersion, orientation, and interfacial interactions within the coating [36,121].

One of the highest levels of barrier performance is typically obtained in multilayer architectures, where multiple functional layers are combined to create a hierarchical resistance to mass transport. In these systems, barrier behaviour results from the cumulative effect of sequential layers, each acting as an additional resistance step. This configuration reduces the probability of defect alignment across the thickness and introduces multiple diffusion barriers, explaining why multilayer systems often outperform thicker single-layer coatings [26,155,156].

From a quantitative perspective, optimized coated and multilayer systems have been shown to achieve in optimized cases oxygen transmission rates below 1 cc/m^2^·day and water vapour transmission rates close to or below 1 g/m^2^·day, approaching the performance of conventional polymer-based packaging materials [104]. However, barrier performance spans several orders of magnitude depending on coating composition, structural architecture, and processing conditions, highlighting the strong variability of these systems [51].

Despite these advancements, a key limitation of many bio-based coatings remains their sensitivity to moisture. Under high relative humidity, hydrophilic materials such as starch, nanocellulose, and polyvinyl alcohol absorb water, leading to swelling, increased free volume, and a consequent increase in permeability. This behaviour can significantly compromise barrier performance under realistic service conditions and often necessitates the use of multilayer or hybrid configurations [96,121].

Barrier performance is not governed by a single transport mechanism, but emerges from the interaction between the permeating species and the material structure. Gases such as oxygen generally follow solution–diffusion mechanisms, but their transport is strongly influenced by coating continuity, polarity, and the presence of tortuous pathways introduced by fillers or multilayer architectures. In contrast, water vapour transport is highly sensitive to sorption phenomena, as hydrophilic materials absorb moisture, leading to swelling and structural changes that increase permeability.

Grease resistance is primarily controlled by surface-related mechanisms, including wettability and surface energy, and is therefore less dependent on bulk diffusion compared to gas transport. Hydrophobic and low-energy surfaces can effectively prevent oil penetration even in systems with moderate gas barrier performance. Similarly, light barrier properties are governed by absorption and scattering phenomena, which depend on thickness, morphology, and the presence of pigments or metallic layers rather than on molecular transport processes.

As a result, barrier behaviour cannot be attributed to a single mechanism for each permeant, but must be interpreted as the outcome of combined effects involving material chemistry, structural architecture, and interfacial quality. These structure–property relationships are summarised in Table 12, where the dominant transport mechanisms governing barrier behaviour across different material systems are compared. Table 13 complements this analysis by providing a system-level comparison of barrier performance for uncoated paper substrates, densified systems, coated materials, nanostructured layers, and multilayer architectures. Qualitative levels are expressed using the following unified scale: Low—Moderate—Good—Very good—Excellent. These levels are derived from trends reported across the literature rather than from a single dataset.

Overall, barrier performance follows a hierarchical trend associated with the progressive control of mass transport pathways. However, this trend is not universal across all permeating species. Gas barrier is primarily governed by diffusion and tortuosity effects, whereas water vapour transport is strongly influenced by sorption and swelling phenomena. Grease resistance is controlled by surface properties, while the light barrier depends on absorption and scattering mechanisms. As a result, the optimal material architecture depends on the specific barrier requirement rather than on a single performance metric.

#### 4.2.3. Sealability, Adhesion and Layer Stability

Sealability, adhesion, and layer stability represent critical functional requirements in paper-based packaging systems, as they govern the integrity of joints, the cohesion between layers, and the overall durability of multilayer architectures during processing and service. Unlike mechanical strength, which is largely controlled by the fibrous substrate, these properties are primarily dictated by interfacial phenomena and by the physicochemical compatibility between materials [133,149].

From a mechanistic perspective, the behaviour of these properties is governed by the combined effects of interfacial energy, molecular mobility, and the formation of cohesive or adhesive interactions across material boundaries. In thermoplastic systems, seal formation is driven by temperature-activated processes including softening, wetting, and subsequent molecular interdiffusion across the interface, leading to the development of interpenetrated polymer networks whose strength increases with diffusion time and temperature [157,158]. The evolution of seal strength is therefore intrinsically linked to processing parameters such as temperature, pressure, and dwell time, which control both the extent of interfacial contact and the kinetics of chain mobility [157,159].

At the simplest level, uncoated paper exhibits poor sealability and limited interfacial adhesion due to its porous and heterogeneous surface. The absence of a continuous phase prevents uniform stress transfer and limits the formation of stable interfacial bonds. In such systems, bonding is primarily governed by mechanical interlocking and localized fiber entanglement rather than true intermolecular adhesion, resulting in low seal strength and poor reproducibility [133,156].

Fiber-network densification improves surface uniformity, increases fiber–fiber contact, and enhances intrinsic adhesion through stronger hydrogen bonding and mechanical interlocking. However, the absence of a thermoplastic phase prevents wetting, chain mobility, and interdiffusion across interfaces. Consequently, densified paper provides better interfacial quality than untreated substrates but remains unsuitable for effective thermoplastic heat sealing.

The introduction of surface coatings represents the first effective strategy to improve both sealability and adhesion by creating a more uniform and continuous interface. Bio-based coatings, such as polysaccharide or protein-based systems, enhance surface smoothness and reduce porosity, enabling improved contact between layers. However, their sealing performance remains limited by restricted molecular mobility and strong sensitivity to moisture, which affects both interfacial interactions and mechanical stability. In these systems, bonding is mainly associated with hydrogen bonding and secondary interactions, resulting in moderate adhesion strength and limited resistance to environmental variations [96,160].

Polymeric coatings provide a significant enhancement in sealability due to their thermoplastic behaviour and ability to form continuous films. During heat sealing, these materials undergo softening or melting, allowing wetting of the interface followed by diffusion of polymer chains and the formation of interpenetrated networks across the contact region. The development of interfacial entanglements leads to cohesive bonding, with seal strength increasing as a function of temperature and contact time due to diffusion-controlled kinetics [157,158]. This mechanism enables higher seal strength, improved process stability, and wider operating windows compared to bio-based systems. Additionally, the concept of hot tack—defined as the seal strength immediately after formation before full solidification—plays a critical role in high-speed packaging operations, where insufficient melt strength may lead to premature failure even when final seal strength is adequate [158].

Reinforced and nanostructured coatings introduce additional complexity in interfacial behaviour. The incorporation of fillers or nanostructures can, in some cases, enhance mechanical integrity and reduce defect formation, but may simultaneously restrict chain mobility and hinder interfacial diffusion during sealing. As a result, sealability depends on a balance between reinforcement and processability, and excessive filler content can lead to incomplete bonding or reduced seal strength due to limited molecular interpenetration [26,36].

In multilayer systems, adhesion becomes a dominant factor controlling structural integrity. The presence of multiple interfaces introduces potential failure planes, making interfacial compatibility and adhesion strength critical for preventing delamination. In these systems, adhesion is often enhanced through tie layers, compatibilisers, or surface treatments that promote intermolecular interactions across otherwise incompatible materials. The effectiveness of these strategies depends on the ability to establish strong interfacial interactions and to minimise defects such as voids or incomplete wetting [26,104,158].

Layer stability further depends, among other factors, on the resistance of interfaces to mechanical stress, thermal cycling, and environmental exposure. Insufficient interfacial bonding may result in, in some cases, delamination, crack propagation along interfaces, or loss of barrier continuity. Conversely, well-designed interfaces enable efficient stress transfer and contribute to the overall durability of multilayer structures. The presence of defects such as pinholes, coating discontinuities, or drying-induced stresses can further compromise structural stability, highlighting the importance of processing conditions and coating uniformity [133,156].

At the highest level of complexity, laminated systems rely on carefully engineered interfacial architectures to ensure both sealability and long-term stability. In these systems, polymer layers typically provide sealing functionality, while paperboard and other layers contribute structural support and barrier performance. However, strong interfacial bonding may conflict with end-of-life requirements, as highly compatible or chemically bonded interfaces can hinder layer separation and reduce recyclability. This introduces a fundamental trade-off between performance and circularity, which must be addressed through material selection and interfacial design strategies [104,133].

A hierarchical progression can be identified across paper-based packaging systems, ranging from uncoated and densified fiber networks to coated and multilayer architectures. Sealability generally increases with structural complexity due to the introduction of thermoplastic phases enabling interfacial diffusion. In contrast, adhesion and layer stability may show non-linear behaviour, as they are strongly influenced by interfacial compatibility, defect formation, and architectural design. Overall, sealability, adhesion, and layer stability are governed by interfacial and diffusion-driven phenomena rather than bulk material properties alone. Their optimisation requires a system-level approach that integrates material compatibility, thermal behaviour, and processing conditions. As structural complexity increases, the role of interfaces becomes progressively more critical, making interfacial engineering a key factor in the performance and sustainability of advanced paper-based packaging systems.

#### 4.2.4. Converting Compatibility of Paper-Based Systems

Converting compatibility in paper-based packaging systems refers to the ability of a material to undergo industrial operations such as creasing, folding, thermoforming, and sealing while maintaining structural integrity, surface quality, and functional performance. Unlike barrier or mechanical properties evaluated under static conditions, converting behaviour emerges from the interaction between material structure, process parameters, and deformation mechanisms activated during manufacturing.

From a mechanistic perspective, converting operations involve complex stress states combining tensile, compressive, bending, and shear components. In paperboard systems, these stresses are accommodated by the fibrous network through fiber reorientation, bond breakage, and local densification. As a result, convertibility is intrinsically linked to fundamental mechanical properties such as elongation at break, compressive strain, bending stiffness, and frictional behaviour at the tool–material interface [161].

A first key parameter governing converting behaviour is formability, defined as the ability of paperboard to sustain multidirectional deformation without fracture. Formability correlates strongly with tensile strain and elongation at break, which describe the capacity of the fiber network to accommodate deformation before failure [161]. In addition, compressive properties and out-of-plane stiffness play a crucial role in controlling wrinkle formation and structural stability during forming processes. These parameters determine whether deformation is distributed uniformly or localised into defects such as buckling or cracking.

The role of moisture and temperature is central in controlling formability and, more generally, converting behaviour. Moisture acts as a plasticizer for the fiber network, weakening hydrogen bonds and increasing chain mobility, leading to a marked increase in deformability. Significant improvements in formability have been reported when the moisture content increases from ambient to conditioned states [161,162]. However, this effect is strongly non-linear: while moderate moisture enhances deformation capability, excessive moisture reduces tensile strength and structural integrity, promoting instability and premature failure [162]. Temperature further amplifies this behaviour by affecting both moisture transport and interfacial friction, leading to coupled thermo-hygro-mechanical effects that must be carefully controlled.

Creasing and folding behaviour represent another fundamental aspect of convertibility. Creasing induces controlled delamination within the fiber network, reducing bending stiffness and enabling folding along predefined lines. However, an optimal creasing window exists: insufficient creasing does not adequately reduce stiffness, whereas excessive creasing introduces structural damage and crack initiation, ultimately compromising mechanical integrity [162]. This explains why foldability does not increase monotonically with creasing depth but instead reaches a plateau followed by degradation.

In coated and multilayer systems, converting behaviour is further influenced by the presence of functional layers. Coatings modify both mechanical response and failure mechanisms. Flexible coatings, including dispersion-based and plasticized bio-based systems, can enhance extensibility and reduce stress concentration, improving convertibility. In contrast, rigid or brittle coatings tend to crack under deformation, leading to loss of barrier performance and surface defects. Experimental evidence indicates that coating damage is often more sensitive to processing conditions than to intrinsic material properties, with forming speed, temperature, and dwell time playing a decisive role [94]. Higher forming speeds increase damage due to limited stress relaxation, while moderate temperatures and lower speeds promote more uniform deformation. However, excessively high temperatures may induce softening, delamination, or sticking phenomena, highlighting again the existence of optimal processing windows rather than monotonic trends.

These trends are summarised in Table 14, which integrates both converting-related indicators and the qualitative evolution of mechanical performance across the main paper-based packaging architectures.

Printability is primarily governed by surface properties such as roughness, porosity, and coating continuity, which control ink transfer and fixation. Improvements along the structural hierarchy—from uncoated paper to coated and multilayer systems—are associated with increased surface uniformity and reduced absorption variability. Thermoformability reflects the ability of the material to undergo large, temperature-assisted deformations and is strongly enhanced by the presence of polymeric phases and thermally responsive components, which enable stress redistribution during forming [163,164]. Process stability describes the robustness of the material under industrial conditions, including sensitivity to temperature, humidity, and processing speed, and generally improves with increasing structural control and material uniformity [94,162].

In contrast, folding and creasing resistance exhibit a non-monotonic behaviour. While uncoated paper benefits from high intrinsic flexibility, the introduction of coatings may reduce deformability due to increased stiffness or brittleness. Polymeric coatings can partially restore flexibility, whereas multilayer architectures may introduce interfacial constraints and differential strain, leading again to reduced foldability. This competing behaviour reflects the balance between flexibility, coating properties, and interfacial adhesion.

Overall, converting compatibility emerges as a balance between deformability and structural stability. Systems that maximise extensibility through plasticization or soft coatings may suffer from reduced strength and dimensional stability, while highly engineered multilayer systems may fail due to interfacial stress concentration and mismatch. As a result, optimal performance is not achieved at the extremes but within a defined processing window governed by material composition, moisture content, temperature, and processing conditions. This balance underlies the trends reported in Figure 12 and provides the framework for interpreting the comparative results summarised in Table 14.

## 5. Mass Transfer, Migration, Safety and Regulatory Constraints

Mass transfer phenomena represent a fundamental aspect governing the performance, safety, and functional reliability of paper-based packaging systems. In this context, migration is treated as a specific manifestation of mass transfer occurring in food-contact systems, while in non-food applications, similar transport processes are more appropriately described as release, emission, or permeation phenomena. Unlike dense and non-porous materials, paper and paperboard are inherently porous, hygroscopic, and structurally heterogeneous, which makes them particularly susceptible to the transport of gases, vapours, and low-molecular-weight compounds [2,165]. In coated and multilayer configurations, mass transfer behaviour is no longer dictated solely by the fibrous substrate, but emerges from the combined effect of material composition, layer architecture, and interfacial integrity [2].

From a physical standpoint, mass transfer in paper-based systems involves multiple concurrent mechanisms, including diffusion through continuous phases, permeation across layered structures, and sorption–desorption processes within the fiber network [166,167]. The relative contribution of each mechanism depends on the chemical nature of the migrating species, the polarity and crystallinity of the coating materials, and the presence of structural discontinuities such as pores, microcracks, or imperfect interfaces. As a result, the effectiveness of a given packaging solution is not determined exclusively by the intrinsic properties of individual layers but by the overall coherence of the multilayer architecture.

Although migration phenomena are most extensively studied in food-contact materials, the underlying mass transfer mechanisms are more general and are also relevant in other paper-based packaging contexts. From an application perspective, it is therefore important to distinguish between migration in food-contact systems and mass transfer phenomena occurring in non-food applications, although both originate from the same underlying physicochemical processes. In food packaging, migration typically refers to the transfer of chemical substances from the packaging material into the food, with direct implications for consumer safety and regulatory compliance. For example, low-molecular-weight compounds such as mineral oil hydrocarbons (MOSH) and mineral oil aromatic hydrocarbons (MOAH) or additives may diffuse through paperboard and contaminate dry food products.

In contrast, in non-food applications, similar transport processes manifest in different forms. In industrial paper sacks used for cement or mineral powders, mass transfer is primarily associated with the release of particulate matter due to mechanical stresses and structural discontinuities. In corrugated board used for electronic or technical products, the emission of volatile organic compounds (VOCs) from recycled fibers, inks, or adhesives may lead to contamination of sensitive components. In other cases, paper-based materials may act as sorption media, temporarily retaining chemicals such as agroactive compounds and subsequently releasing them over time through desorption-driven mechanisms.

The section first introduces the fundamental mechanisms governing mass transfer in paper-based systems, followed by migration phenomena in food packaging, regulatory and safety aspects, non-food applications, and functional barrier design.

### 5.1. Fundamental Mechanisms of Mass Transfer in Paper-Based Systems

Mass transfer in paper-based materials results from the coupling of diffusion, permeation, and sorption–desorption processes, governed by the multiscale structure of the fibrous network.

Unlike dense polymers, paper and paperboard exhibit a porous architecture in which transport occurs through fiber walls, inter-fiber voids, and coating layers, leading to non-uniform mass transfer [165]. In this sense, the term mass transfer is used here in a broad physicochemical meaning, encompassing both migration toward food-contact media and release phenomena toward surrounding environments or confined atmospheres.

From a mechanistic standpoint, diffusion remains the fundamental driving process and is controlled by concentration gradients. However, in cellulose-based systems, diffusion is strongly coupled with sorption phenomena due to the polar nature of hydroxyl groups and the high specific surface area of fibers. Experimental studies on cellulose-based and paper materials have shown that moisture uptake follows non-linear sorption isotherms, commonly described using Guggenheim–Andersen–de Boer (GAB)-type models, with increasing water activity leading to significant changes in transport behaviour and diffusion properties [168]. This confirms that transport cannot be described as purely Fickian but must include partitioning and time-dependent retention effects within the fiber network.

The role of sorption is particularly relevant for volatile and semi-volatile compounds. Experimental investigations on cellulosic materials have shown that volatile organic compounds (VOCs) and low-molecular-weight species can be temporarily retained within the matrix and subsequently released, generating delayed emission profiles and non-steady-state transport conditions [169]. This behaviour is governed by the relative affinity between the migrating species and the cellulose matrix, as well as by environmental parameters such as temperature, which directly affects diffusivity and vapor pressure.

The introduction of coatings and nanostructured layers modifies transport by reducing pore connectivity and increasing diffusion path tortuosity. Microfibrillated cellulose (MFC) coatings, for example, have been shown to significantly decrease air permeability by forming dense fibrillar networks that reduce effective porosity and create extended diffusion pathways [170]. In such systems, transport becomes dominated by diffusion through a quasi-continuous phase rather than by flow through interconnected pores.

However, barrier performance is not solely determined by intrinsic material properties. Structural discontinuities, such as incomplete coating coverage or interfacial defects, may create preferential pathways that dominate overall mass transfer. This explains why multilayer and coated systems can still exhibit measurable migration or release even when nominal barrier layers are present.

Moisture represents a critical parameter controlling mass transfer. Experimental evidence demonstrates that increasing relative humidity leads to a sharp increase in permeability due to water-induced plasticisation and swelling of the cellulose network. In MFC-based systems, oxygen permeability has been shown to increase exponentially above critical moisture contents, as the material transitions from a rigid to a more rubbery state [170]. This effect is associated with increased free volume and enhanced molecular mobility, which accelerate diffusion processes.

Finally, mass transfer in paper-based systems must be interpreted as a system-level phenomenon, where material composition, environmental conditions, and the physicochemical properties of migrating species jointly determine transport behaviour. These mechanisms provide the basis for understanding migration in food-contact systems and release processes in non-food applications [171].

### 5.2. Migration in Food Packaging Systems

In food-contact systems, migration represents a specific manifestation of mass transfer, referring to the transfer of chemical substances from packaging materials into food or food simulants. Unlike the general transport phenomena discussed in the previous section, migration is governed not only by coupled diffusion–sorption mechanisms but also by the physicochemical properties of the contacting medium, which directly affect partitioning, driving forces, and transport kinetics. This phenomenon has been extensively reviewed in the literature, particularly in relation to fiber-based materials, where structural heterogeneity and the presence of residual contaminants complicate predictive modelling [172,173].

A first distinction must be made between uncoated paper, coated systems, and multilayer architectures, as these configurations exhibit fundamentally different transport regimes and migration profiles.

In uncoated paper and paperboard, migration is primarily governed by the intrinsic porosity of the fibrous network. The interconnected void structure enables relatively fast transport of low-molecular-weight compounds, particularly under dry-contact conditions where pore continuity remains high. In these systems, the fibrous matrix acts simultaneously as a transport pathway and as a sorption reservoir, leading to rapid initial transfer followed by slower release controlled by desorption kinetics. This behaviour is particularly critical in recycled paperboard, where mineral oil hydrocarbons (MOSH/MOAH), printing ink residues, and other contaminants can be present and migrate into food. The presence of non-intentionally added substances (NIAS) further complicates the scenario, as these compounds originate from degradation processes, recycling streams, or impurities and are often only partially identified [173,174]. Migration from these systems is therefore not only diffusion-driven but also controlled by release kinetics from the cellulose matrix, resulting in delayed and non-steady-state transfer profiles.

In coated paper systems, transport shifts from pore-controlled to diffusion-controlled behaviour. Coatings reduce pore connectivity and impose a barrier whose effectiveness depends on continuity, morphology, and thickness. Nanostructured coatings further enhance performance by increasing tortuosity and reducing effective porosity [23,167]. However, hydrophilic coatings remain sensitive to humidity-induced plasticisation, which may strongly increase diffusivity under realistic service conditions [169], whereas hydrophobic coatings may still allow selective migration of non-polar compounds depending on chemical affinity.

Multilayer systems introduce an additional level of complexity, as migration is governed by the sequential interaction of layers with distinct physicochemical properties. Functional barrier layers are designed to limit migration from underlying substrates, especially in recycled fiber-based materials; however, their effectiveness is often compromised by structural imperfections such as pinholes, incomplete coverage, or interfacial discontinuities. In such cases, migration is no longer controlled by bulk diffusion but by defect-mediated pathways, which can dominate overall transport even when the intrinsic barrier properties of the material are high [172]. This explains the frequent discrepancies observed between idealised laboratory measurements and real packaging performance, highlighting the limitations of standardised testing approaches and the need for more realistic, application-specific migration assessment protocols.

The composition of the contacting food further modulates migration behaviour. Fatty foods enhance the migration of lipophilic compounds by increasing partitioning toward the food phase, whereas aqueous systems promote transport through swelling and plasticisation of hydrophilic components. Even in dry-food applications, migration may occur via the gas phase, particularly for volatile compounds in confined packaging systems. These interactions between material, migrant, and food matrix are critical for realistic risk assessment and have been extensively discussed in modelling and regulatory studies [166,175].

Recent studies have also highlighted the presence of emerging contaminants in paper-based packaging, including per- and polyfluoroalkyl substances (PFAS), which are widely used for grease resistance. These compounds exhibit high chemical stability and mobility and have been detected in a wide range of paper and board materials. For instance, measurable concentrations of PFAS have been reported in food-contact papers and boards, with some compounds detected systematically across samples, indicating persistent contamination sources and significant migration potential [173]. However, specific data on PFAS occurrence and migration in paper-based systems are not directly covered in the present sources. Their relatively high mobility, especially for short-chain analogues, further increases the likelihood of transfer into food matrices, raising concerns for long-term exposure and regulatory compliance.

Overall, migration behaviour depends on the interaction between structural configuration, environmental conditions, and chemical affinity between migrants, packaging materials, and food matrices. Effective migration control therefore requires a system-level approach integrating material selection, coating architecture, and application-specific conditions [173,175].

### 5.3. Regulatory Framework for Migration in Paper-Based Packaging

**Figure 16:**
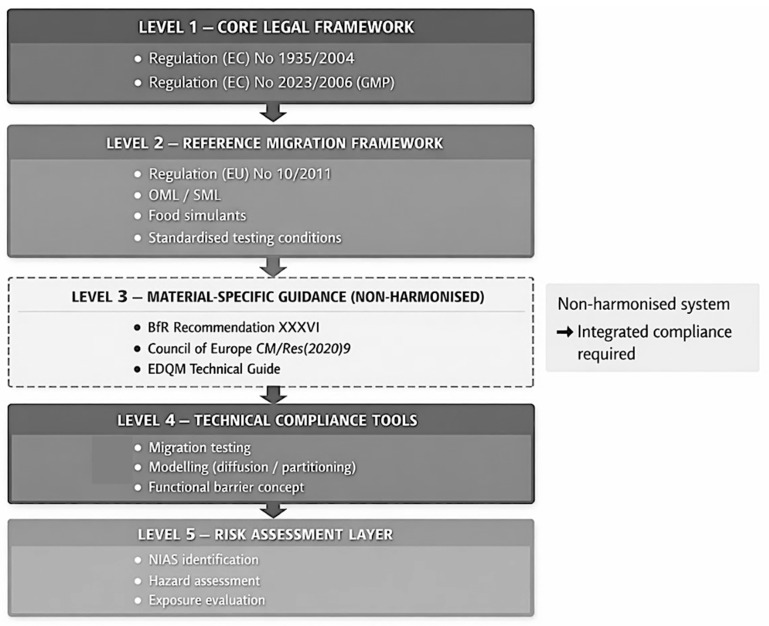
**Multi-level regulatory framework governing migration in paper-based packaging.** The scheme illustrates the hierarchical organisation of (i) European Union (EU) legislation (Reg. 1935/2004; GMP 2023/2006), (ii) reference migration frameworks (EU 10/2011), (iii) material-specific guidance (BfR, Council of Europe, CEPI), (iv) technical assessment tools (migration testing, modelling, functional barriers), and (v) risk assessment approaches (NIAS evaluation). The framework translates underlying mass transfer mechanisms into measurable compliance criteria and highlights the distinction between food-contact systems, regulated through migration limits, and non-food applications, governed primarily by containment and performance requirements.

Migration phenomena in packaging materials, described in the previous sections in terms of diffusion, sorption, permeation, and defect-mediated transport, are not evaluated solely on a physicochemical basis but are translated into regulatory compliance criteria due to their direct implications for safety and product quality. In paper-based packaging systems, this translation results in a multi-level framework integrating legislation, reference migration criteria, material-specific guidance, and technical risk assessment tools. The overall structure of this framework is schematically illustrated in Figure 16 and summarised in Table 15.

A fundamental distinction must be made between food-contact applications and non-food or transport packaging systems. In food-contact materials, migration is directly associated with human exposure and is therefore subject to strict safety requirements and, where applicable, quantitative limits. In contrast, for non-food applications, no equivalent harmonised migration limits are generally defined. Instead, material performance is assessed in terms of containment, compatibility, and resistance to permeation or release, even though the same underlying mass transfer mechanisms apply. As a result, migration becomes a regulated parameter primarily in the context of food safety, while in non-food systems, it is addressed indirectly through performance-based criteria.

At the first level, the core legal framework for food-contact materials is defined by Regulation (EC) No 1935/2004 [176], which establishes the general safety principle requiring that materials do not transfer constituents to food in quantities that could endanger human health, alter food composition, or impair organoleptic properties. This requirement is complemented by Regulation (EC) No 2023/2006 [177] on Good Manufacturing Practice (GMP), which links migration control to process consistency, traceability, and quality assurance. In paper-based systems, this aspect is particularly critical due to the variability associated with fibrous raw materials, recycled content, inks, adhesives, and converting processes.

At the second level, Regulation (EU) No 10/2011 [178], although formally applicable to plastics, provides the main reference framework for migration testing and interpretation, introducing overall migration limits (OML), specific migration limits (SML), food simulants, and standardised time–temperature conditions.

However, the direct applicability of Regulation (EU) No 10/2011 to paper-based systems remains limited, as the assumptions of homogeneous, diffusion-controlled transport do not fully capture the heterogeneous and porous nature of fiber-based materials. These concepts are widely applied by analogy to coated and multilayer paper-based systems when migration is governed by diffusion through continuous polymeric layers. The theoretical basis of such approaches is grounded in diffusion-controlled transport and partitioning phenomena, as established in modelling studies (Piringer, 1997) [179], and remains central for interpreting experimental results and predicting long-term behaviour. More recent analyses (Störmer et al., 2024) [172] highlight that, for heterogeneous systems such as paper and board, migration behaviour may deviate from idealised assumptions due to porosity, multilayer complexity, and non-uniform transport pathways.

At the third level, in the absence of harmonised European legislation specifically addressing paper and board, compliance relies on non-binding but widely adopted material-specific guidance. BfR Recommendation XXXVI [180] defines compositional requirements and restrictions for substances used in paper and board intended for food contact, while the Council of Europe Resolution CM/Res(2020)9 [181] and the associated EDQM technical guide provides a broader risk-based evaluation framework. In addition, industry guidelines such as those developed by CEPI [182] contribute to the practical implementation of safety and compliance strategies across the production chain. As discussed in the literature [172], the coexistence of these documents reflects a fragmented and partially non-harmonised regulatory landscape for paper-based materials.

At the fourth level, regulatory compliance is operationally implemented through technical assessment tools used to quantify and predict migration behaviour. These include experimental migration testing under standardised conditions, modelling approaches based on diffusion and partitioning, and the application of the functional barrier concept in multilayer and recycled systems. Functional barriers are designed to limit the transfer of substances from non-food-contact layers or recycled substrates into food; however, in paper-based materials, their effectiveness may be affected by structural heterogeneity, interfacial discontinuities, and incomplete coating coverage, leading to deviations from idealised barrier performance.

At the fifth level, compliance assessment incorporates risk evaluation strategies, particularly for non-intentionally added substances (NIAS). These substances originate from impurities, degradation reactions, recycling processes, or interactions between components. Their evaluation requires a structured approach, including hazard identification, exposure assessment, and toxicological analysis, supported by analytical screening and modelling tools [183]. This aspect is especially relevant for paper-based systems, where the fibrous matrix may act as a reservoir for contaminants originating from previous use cycles or processing steps.

The framework summarised in Table 15 highlights that migration control in paper-based packaging is governed by a hierarchical but non-harmonised system, in which legislation, guidance, modelling, and industrial practice interact. This structure reflects the intrinsic complexity of paper-based materials and establishes a direct link between mass transfer mechanisms, material design, and regulatory compliance. Consequently, migration control cannot be addressed solely through intrinsic material properties, but requires a system-level approach integrating transport phenomena, multilayer architecture, and application-specific conditions.

**Table 15:** **Regulatory documents, technical guidance, and compliance tools in paper-based food packaging systems**. Overview of the main European regulations, non-harmonised paper and board guidelines, and supporting technical documents relevant to migration assessment in food-contact applications. The table summarises their scope, relevance, and role in compliance evaluation, highlighting the combined use of general safety requirements, good manufacturing practice, reference migration criteria, and paper-specific guidance in the absence of harmonised EU legislation dedicated to paper and board materials.

Regulation/Document	Type	Scope	Key Content	Relevance for Paper-Based Systems
Regulation (EC) No 1935/2004 [176]	EU Regulation	All food-contact materials	General safety principle; no transfer that endangers health, changes food composition unacceptably, or impairs organoleptic properties	Core legal framework applicable to paper and board
Regulation (EC) No 2023/2006 (GMP) [177]	EU Regulation	All food-contact materials	Good Manufacturing Practice; documented quality assurance, process control, traceability	Critical for controlling variability and contamination in fibrous and recycled systems
Regulation (EU) No 10/2011 [178]	EU Regulation	Plastic food-contact materials	OML, SML, simulants, standardised testing conditions	Main methodological reference for coated, laminated, and multilayer paper systems with polymeric phases
BfR Recommendation XXXVI [180]	National recommendation/guidance	Paper and board food-contact materials	Requirements and restrictions for raw materials, additives, contaminants, extracts, and specific migration-related criteria	Widely used practical reference in the absence of EU harmonisation
Council of Europe CM/Res(2020)9 [181]	European non-binding resolution	Non-harmonised food-contact materials, including paper and board	Risk-based safety framework; guiding principles; link to technical guides	Important supranational framework where no specific EU measure exists
EDQM Technical Guide for paper and board [184]	Technical guidance	Paper and board food-contact materials	Supporting documentation, testing strategy, compliance evaluation	Practical implementation tool alongside CM/Res(2020)9
CEPI food-contact guidelines (2019/2021) [182]	CEPI industry guideline	Paper and board materials and articles for food contact	Recommendations for compliance work, test interpretation, limitations of extracts, case-by-case approach	Useful sector guidance for manufacturers and converters
Functional barrier concept	Technical/compliance concept	Multilayer and recycled systems	Barrier-based limitation of transfer from non-food-contact layers or contaminated cores	Essential for coated, laminated, and recycled paper structures
Migration testing protocols	Technical methodology	Food-contact systems	Simulants, time–temperature conditions, extract and migration procedures	Core operational tools, but often requiring cautious interpretation for paper
Modelling approaches (diffusion/partitioning) [179]	Technical tool	Multilayer and barrier systems	Prediction of transfer based on diffusion, partitioning, thickness, and contact conditions	Supporting tool for compliance, especially where direct testing is not fully representative
NIAS assessment [183,185]	Risk-assessment framework	All food-contact materials	Identification, screening, exposure assessment, toxicological evaluation	Particularly critical for recycled paper, inks, adhesives, and complex multilayer systems

From a broader system-level perspective, migration control represents a safety-driven constraint governing the design of paper-based packaging systems. However, this is only one dimension of regulatory compliance. End-of-life performance introduces a complementary set of requirements associated with circularity, including recyclability, compostability, and material recovery. These constraints act on the same material systems and structural features, meaning that coating design, multilayer architecture, and interfacial properties simultaneously influence both safety and circularity outcomes.

### 5.4. Mass Transfer and Release Phenomena

Mass transfer phenomena in non-food packaging applications are governed by the same transport mechanisms described for food-contact systems [172,173], but their manifestation depends strongly on the nature of the transported species and the interaction between the material and the surrounding environment. In this context, it is useful to distinguish between migration, typically associated with transfer into contacting media, and emission or release processes occurring toward surrounding environments. Transport is therefore not limited to diffusion-driven migration but includes a broader range of processes such as volatility-driven emission, sorption–desorption cycles within the fibrous network, and mechanically induced particle release.

From a mechanistic perspective, diffusion and permeation remain central for volatile and semi-volatile compounds.

These species can migrate through the porous fiber network or across coating layers, depending on the structural configuration of the material. In paper-based systems, however, diffusion is rarely a purely Fickian process, as it is strongly coupled with sorption phenomena due to the polar nature and high specific surface area of cellulose. As a result, transport is governed by a combination of diffusion through accessible pathways and partitioning between the fiber matrix and the surrounding environment.

Sorption–desorption processes play a particularly important role in non-food applications. Cellulosic materials can act as temporary reservoirs for organic compounds, including solvents, additives, or active substances, which may be absorbed during processing or storage and subsequently released over time. This leads to time-dependent and non-equilibrium transport behaviour, where release kinetics are controlled not only by diffusion coefficients but also by the affinity between the transported species and the cellulose matrix, as well as by environmental parameters such as temperature and humidity [150,165]. These mechanisms are analogous to those observed in food-contact systems, although their consequences in non-food applications are typically related to product performance or environmental exposure rather than direct ingestion.

Volatile organic compound (VOC) emission represents one of the most relevant manifestations of mass transfer in non-food packaging. Paper and board materials, particularly those containing recycled fibers, may release organic compounds originating from inks, adhesives, coatings, or degradation processes. In such systems, emission is governed by the coupling between internal diffusion and surface desorption, resulting in continuous or delayed release profiles. These processes are strongly influenced by environmental conditions, especially temperature and air exchange, and can lead to the accumulation of contaminants in confined environments. Similar transport behaviour has been widely discussed in the context of material emissions and indoor air quality, where porous materials act as both sources and sinks of volatile compounds.

In addition to molecular transport, mass transfer in non-food packaging may also involve mechanically driven processes. In paper-based systems used for packaging powdered materials, such as industrial sacks, the release of particulate matter is primarily governed by convective transport and mechanical detachment rather than diffusion. Fiber network discontinuities, inter-fiber voids, and structural defects can facilitate particle escape under dynamic conditions such as filling, vibration, and handling. Unlike diffusion-driven processes, these phenomena are controlled by mechanical stresses and airflow conditions and therefore require a different analytical framework.

Overall, mass transfer in non-food applications highlights the dynamic nature of paper-based materials, which may act simultaneously as transport media, temporary reservoirs, and emission sources. Consequently, their behaviour must be interpreted through a system-level approach integrating material structure, environmental interactions, and the physicochemical properties of the transported species.

### 5.5. Functional Barrier Design and Failure Mechanisms

The control of mass transfer in paper-based packaging systems relies on the design of functional barriers capable of limiting transport while maintaining structural integrity throughout the product lifecycle. Barrier performance is not determined solely by the intrinsic permeability of coating materials, but by the combined effects of layer thickness, continuity, and interfacial adhesion, as recognised in migration and modelling [166,172].

A key requirement in barrier design is the suppression of preferential transport pathways. Even with low-permeability materials, defects such as pinholes, microcracks, or incomplete coating coverage can dominate mass transfer by providing low-resistance routes. In fiber-based systems, deviations between expected and measured performance are frequently attributed to such structural discontinuities, indicating that transport is often governed by defect-mediated mechanisms rather than intrinsic material properties [172].

In multilayer architectures, interfacial integrity is equally critical. Barrier effectiveness depends not only on layer properties but also on adhesion between adjacent materials. Poor interfacial compatibility may lead to delamination or interfacial voids, which act as preferential transport channels. Both modelling and experimental studies highlight that transport across layered systems is strongly influenced by interfacial continuity and resistance [166,172].

Processing and converting operations introduce additional constraints. Heat sealing, folding, and mechanical deformation may locally damage coatings and multilayer structures, particularly in sealing regions and creases, where barrier disruption is more likely. Seal integrity is therefore strongly dependent on processing conditions, material compatibility, and thermal history [157,158].

Environmental conditions further modulate barrier performance during service. Moisture uptake in cellulose-based substrates induces swelling and plasticisation, increasing free volume and diffusion rates, as demonstrated for nanocellulose-based coatings under high relative humidity [166]. Temperature similarly affects diffusion coefficients and sorption–desorption kinetics, as described in classical migration models [166].

These considerations highlight that barrier performance must be addressed at the system level. Effective design requires the integration of material selection, coating formulation, interfacial optimisation, and processing conditions, with particular attention to defect control and environmental stability. Accordingly, barrier effectiveness should be evaluated not only in terms of intrinsic material properties but also under realistic operating conditions and structural reliability (Geueke et al., 2018; Muncke et al., 2021) [173,175].

These aspects also underline the strong interconnection between safety and end-of-life performance, addressed in the following section.

## 6. End-of-Life Performance and Circularity of Paper-Based Packaging

While previous sections have addressed material design, functional performance, and safety constraints through migration control, these same design strategies inherently introduce system-level implications that directly affect end-of-life (EoL) behaviour.

In paper-based packaging, improvements in barrier performance, mechanical integrity, and processability are often achieved at the expense of material separability, recyclability, and compatibility with existing waste-management infrastructures. Consequently, EoL performance should be considered an integral component of material and structural design rather than a downstream evaluation step.

End-of-life (EoL) refers to the set of processes through which packaging materials are treated after use, including recycling, composting, energy recovery, and landfill disposal. Within this framework, circularity describes the capacity of a material system to re-enter productive cycles through material recovery or controlled degradation, thereby preserving material value and reducing resource loss. In paper-based systems, this capability is governed by the interaction between the fibrous substrate, functional layers, additives, and interfaces, which collectively determine processability during recycling, degradation pathways, and material recovery efficiency.

This section adopts a system-level perspective to analyse the mechanisms governing EoL performance. Section 6.1 examines the main end-of-life pathways, with particular emphasis on recycling and repulpability constraints in coated and multilayer systems. Section 6.2 focuses on compostability and biodegradability, clarifying their definitions, operational conditions, and applicability. Section 6.3 addresses interfacial constraints and material incompatibilities, highlighting how structural design governs trade-offs between functional performance and circularity.

**Figure 17:**
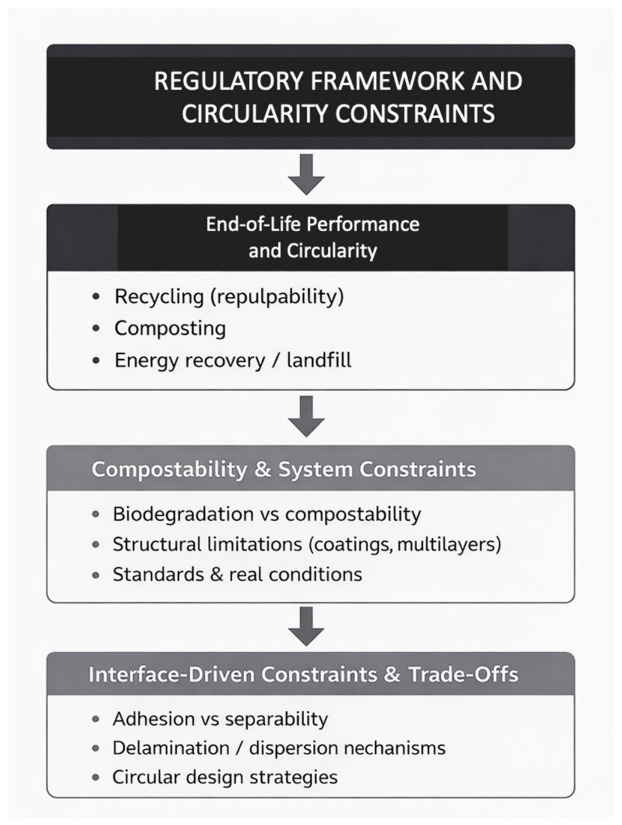
**System-level roadmap of end-of-life performance in paper-based packaging.** The diagram illustrates the progression from regulatory constraints to end-of-life pathways, compostability conditions, and interface-driven limitations. It highlights how circularity requirements, defined by current regulatory frameworks, influence material selection, structural design, and recovery efficiency in paper-based packaging systems.

The logical progression of these aspects is illustrated in Figure 17, which provides a roadmap linking end-of-life pathways, material constraints, and interface-driven design trade-offs within a unified framework.

In this context, circularity-related constraints complement the safety-driven regulatory framework discussed in Section 5. While migration regulations define requirements related to substance transfer and food-contact compliance, end-of-life frameworks introduce additional constraints associated with recyclability, compostability, and material recovery. Together, these domains define a dual set of design requirements acting on the same material systems and structural features.

### 6.1. From Compostability to Circularity: Regulatory Implications for Paper-Based Packaging

Early regulatory approaches to packaging end-of-life were established within the European framework through a set of harmonized European Committee for Standardization (CEN) standards developed under Directive 94/62/EC. These include EN 13427 (general requirements) [186], EN 13428 (source reduction) [187], EN 13430 (material recycling) [188], and EN 13432 (organic recovery) [189], which collectively define the main design and recovery criteria for packaging systems. Within this framework, minimisation, recyclability, and compostability are treated as distinct and non-interchangeable dimensions, each associated with specific technical requirements and operational pathways.

In parallel, scientific research focused on clarifying material-level degradation mechanisms. In this context, Mojo et al. [190] provided a structured distinction between degradable, biodegradable, and compostable systems, linking these definitions to standardised test methods such as ASTM D6400 [191] and ASTM D6868 [192] (published by ASTM International), particularly relevant for coated paper-based materials. However, this approach remained largely centred on intrinsic material behaviour under controlled conditions and did not explicitly address the interaction between packaging systems and real waste management infrastructures.

More recent regulatory developments have progressively shifted the perspective from material properties to system compatibility, introducing circularity as a primary design constraint. Under the Packaging and Packaging Waste Regulation (PPWR) [191], packaging performance is increasingly evaluated according to its compatibility with collection, sorting, recycling, and reuse systems, making recyclability a design outcome rather than an intrinsic material [192].

For paper-based packaging, this evolution has direct and non-trivial implications. While paper and cardboard are intrinsically compatible with fiber-based recycling streams, their effective circularity is strongly influenced by the introduction of coatings, laminates, and functional layers. According to CEPI guidelines [182], even limited amounts of non-fibrous components may interfere with pulping efficiency, contaminant removal, and fiber quality, highlighting the importance of repulpability and process compatibility as key design criteria.

As a result, different paper-based architectures exhibit markedly different circularity profiles. Uncoated fibrous systems remain the most compatible with recycling processes, ensuring efficient fiber recovery and minimal contamination. Coated systems require careful formulation and verification, as their recyclability depends on coating dispersibility, adhesion, and chemical composition. Multilayer and laminated structures, although offering superior barrier performance, represent the most critical case, as their structural complexity often limits separability and compromises recycling efficiency unless specifically engineered for disintegration or delamination.

Compostability alone does not guarantee circularity. Standardised compostability tests are performed under controlled conditions and may not reflect real waste-management environments, while degradation behaviour remains strongly dependent on both material properties and operating conditions [193,194].

From a system perspective, further limitations arise from the structure of existing waste management frameworks. The lack of harmonised regulations, insufficient coordination between stakeholders, limited composting infrastructure, and operational issues such as cross-contamination and improper waste segregation significantly constrain the effective implementation of compostable packaging [193].

At the same time, global regulatory analyses highlight that packaging sustainability is increasingly governed through integrated circular economy strategies, where end-of-life performance depends on the alignment between material design, infrastructure, and policy frameworks rather than on material properties alone [194].

Overall, current circularity-oriented regulations require paper-based packaging to be designed at the system level, considering substrates, coatings, interfaces, and multilayer architectures together rather than as independent components.

### 6.2. End-of-Life Pathways

Paper-based packaging can follow multiple end-of-life pathways, including recycling, composting, energy recovery, and landfill disposal. The selection and effectiveness of these routes depend on both the material composition and the structural complexity of the system.

Among these pathways, recycling represents the primary route for paper-based materials. In conventional recycling processes, paper is subjected to repulping, i.e., hydromechanical disintegration in water, which enables the recovery of cellulose fibers. In uncoated systems, this process is generally efficient, as fibers are easily separated and non-fibrous contaminants are minimal, allowing high fiber recovery and acceptable pulp quality (CEPI, 2019) [182].

In coated systems, the presence of functional layers alters this behaviour. Polymeric coatings, waxes, and additives may not disperse during repulping, remaining attached to fibers or forming dispersed particles. These residues can interfere with fiber recovery, reduce pulp quality, and generate operational issues such as stickies and filtration difficulties [195,196].

Recycling becomes even more challenging in multilayer architectures, where strong interfacial adhesion and material incompatibility hinder separation and generate mixed-material residues [131,197].

When recycling is not feasible, alternative end-of-life pathways are considered. Composting may represent a viable route for systems composed of biodegradable materials, although its effectiveness depends on the ability of the structure to disintegrate and degrade under controlled conditions. In practice, the presence of non-biodegradable components or persistent additives may limit the applicability of this pathway.

In cases where neither recycling nor composting is suitable, materials are typically directed to energy recovery through incineration or, less desirably, to landfill disposal. These routes do not allow material recovery and therefore represent less favourable outcomes from a circularity perspective [194].

Overall, the analysis of end-of-life pathways highlights that the behaviour of paper-based packaging is determined not only by the intrinsic properties of cellulose, but by the entire system architecture. Coatings, interfaces, and material combinations play a decisive role in defining whether a given system can effectively enter a circular loop.

### 6.3. Compostability, Biodegradability, and System Constraints

Compostability is frequently considered a key advantage of paper-based packaging systems, particularly in the context of bio-based and biodegradable materials. However, achieving effective compostability in coated and multilayer systems remains a complex challenge, as it requires the simultaneous fulfilment of material, structural, and process-related conditions.

In uncoated paper, biodegradation occurs relatively rapidly due to the natural susceptibility of cellulose fibers to microbial attack. In coated systems, however, the presence of continuous films or hydrophobic layers may act as physical barriers, limiting water penetration and delaying or inhibiting microbial degradation. As a result, the overall biodegradation rate is often governed by the least degradable component of the system [198,199].

A clear distinction must be made between biodegradability and compostability. Biodegradability refers to the ability of a material to be broken down by microorganisms over time, whereas compostability requires not only biodegradation but also disintegration within a defined timeframe and under controlled conditions.

This distinction is particularly relevant for multilayer systems, where biodegradable and non-biodegradable components may coexist. Even when individual layers are theoretically compostable, strong interfacial adhesion and structural integration may hinder disintegration, preventing the material from meeting compostability requirements [200,201].

From a design perspective, compostability must therefore be addressed at the system level. The selection of biodegradable polymers, the control of coating thickness, and the avoidance of persistent additives are essential to ensure effective degradation. At the same time, these choices may affect barrier performance, mechanical integrity, and sealability, creating a recurring trade-off between functionality and degradability in bio-based and compostable coated paper systems [149,202].

Another important constraint is related to infrastructure and standards. Compostability is typically defined under controlled industrial conditions, which may not be available in all regions. In addition, regulatory requirements may further constrain material selection and design [194].

### 6.4. Interface-Driven Constraints and Application-Oriented Design

In paper-based packaging systems, end-of-life performance is primarily governed by interfacial phenomena arising from the combination of materials with different physicochemical properties. Adhesion strength, layer continuity, and structural integration determine not only functional performance during use but also the efficiency of separation, fiber recovery, and degradation at end-of-life. In multilayer and hybrid systems, strong interfacial bonding may improve barrier integrity and mechanical stability, but can simultaneously hinder delamination and reduce recyclability. Conversely, weak or incompatible interfaces may facilitate material separation but compromise structural performance.

These interfacial constraints generate intrinsic trade-offs between barrier performance, mechanical functionality, processability, and circularity. High-performance barrier systems, typically based on dense coatings, nanostructured layers, or polymer–paper laminates, reduce permeability and extend shelf life, but often introduce challenges in fiber recovery, contaminant removal, and process compatibility during recycling. Simpler coating systems and mono-material approaches, while offering lower barrier efficiency, generally exhibit improved recyclability, reduced processing complexity, and better alignment with circular design strategies.

From a design perspective, these trade-offs imply that no single material architecture can simultaneously maximise all performance indicators. Instead, the selection of paper-based packaging systems must be guided by the specific functional requirements of the target application, considering barrier needs, mechanical constraints, processing conditions, and end-of-life compatibility as interdependent variables.

Applications requiring moderate barrier performance, such as dry food packaging, can be effectively addressed through mono-component or lightly reinforced bio-based coatings, which provide sufficient protection while maintaining high recyclability and process simplicity. In contrast, applications involving fresh produce benefit from semi-permeable systems that allow controlled gas exchange, where excessively dense or impermeable coatings may lead to product degradation. Packaging of fatty or grease-sensitive products requires hydrophobic or reinforced coating systems capable of maintaining performance under mechanical stress and exposure to lipids.

More demanding applications, including modified atmosphere packaging and extended shelf-life systems, require very low permeability levels that can generally be achieved only through multilayer architectures or hybrid systems. While these solutions provide superior barrier performance, they introduce increased structural complexity and reduced compatibility with recycling processes. Similarly, liquid packaging and beverage cartons rely on highly integrated multilayer structures to ensure long-term stability, but their composite nature significantly complicates material separation and recovery.

In contrast, secondary and transport packaging primarily rely on mechanical strength and structural stability, where simpler material architectures offer clear advantages in terms of recyclability, cost, and industrial scalability. In these cases, the use of complex multilayer systems is generally unnecessary and may introduce avoidable end-of-life constraints.

Across all application domains, increasing barrier performance and functional integration generally require greater material complexity, whereas simpler systems favour recyclability and process compatibility. Consequently, paper-based packaging design should be approached as a multidimensional optimisation problem balancing performance, processing requirements, and end-of-life behaviour.

## 7. Future Perspectives and Research Directions

The analysis developed in this review is not intended only as a retrospective synthesis of the literature, but also as a design-oriented framework for interpreting the future evolution of paper-based packaging. By starting from a consolidated state of the art and progressively linking substrates, coatings, multilayer architectures, interfaces, performance requirements, safety constraints, and end-of-life pathways, the review provides a basis for identifying where the field is moving and where future research should be directed. In this sense, the transition from cellulosic substrates to functional and hybrid architectures does not represent only a historical or technological progression, but also a roadmap for the development of next-generation paper-based packaging systems.

The evolution of paper-based packaging highlights a progressive transition from material-driven solutions toward architecture-driven design approaches. While significant advances have been achieved in coating technologies, multilayer structures, and hybrid systems, several critical issues remain unresolved. Future developments are therefore expected to be increasingly governed by structural and architectural design rather than by the intrinsic properties of individual materials alone, requiring integrated system-level strategies that simultaneously address barrier performance, mechanical integrity, converting behaviour, safety, and end-of-life management.

One of the most critical directions concerns the trade-off between functional performance and recyclability. High-barrier multilayer and hybrid systems often improve shelf-life protection but may hinder fiber recovery due to strong interfacial bonding and material incompatibility. Future research should therefore treat recyclability as a primary design constraint rather than as a post-use assessment. Within this framework, design-for-recycling and controlled delamination represent key strategies, enabling the selective separation of functional layers during recycling while preserving structural integrity during use. Reversible, water-dispersible, or stimuli-responsive interfaces may provide promising routes toward packaging systems that combine high performance with improved compatibility with fiber-based recovery pathways.

Moisture stability remains another central research priority. Although many polysaccharide- and protein-based coatings provide excellent oxygen barrier properties under dry conditions, their performance often deteriorates under high relative humidity. Future work should therefore focus on improving moisture tolerance through chemical modification, crosslinking, hybridisation, and multilayer protection strategies, while avoiding solutions that compromise biodegradability, recyclability, or food-contact safety. At the same time, greater attention should be paid to converting compatibility, including folding, creasing, forming, and sealing under realistic industrial conditions. The interaction between coating structure, mechanical response, and processing parameters remains insufficiently understood and represents one of the main gaps between laboratory-scale performance and industrial implementation.

A further development pathway concerns the integration of multiple functions within paper-based systems. Future packaging is expected not only to act as a passive barrier, but also to provide active and intelligent functions, including antimicrobial activity, controlled release, freshness indication, and monitoring of storage conditions. Hybrid systems based on bio-based polymers, nanostructured phases, and functional additives offer significant potential in this direction. However, multifunctionality must be balanced with migration control, NIAS assessment, PFAS-free design, toxicological safety, and end-of-life compatibility. For this reason, the future development of active and intelligent paper-based packaging should be guided by an integrated safety-by-design approach.

Another important limitation of the current literature is the lack of standardised experimental procedures and reporting formats. Differences in substrate type, coating thickness, processing conditions, relative humidity, temperature, and test methods often prevent direct comparison among studies. Future research should therefore adopt more consistent testing protocols and report key parameters in a transparent and comparable manner. This is particularly important for WVTR, OTR, Cobb values, grease resistance, mechanical properties, heat-sealing behaviour, and recyclability indicators. Without this harmonisation, the translation of promising laboratory results into reliable industrial solutions remains difficult.

Finally, scalability and industrial translation should become central elements of future research. Many high-performance coatings and hybrid systems are still developed under laboratory-scale conditions and may not be directly compatible with industrial coating, extrusion, lamination, printing, or converting lines. Future studies should therefore evaluate not only material performance but also processability, cost, production speed, coating stability, drying requirements, compatibility with existing equipment, and end-of-life scenarios.

Life-cycle assessment, recyclability testing, and techno-economic evaluation should be integrated more systematically into the development of new materials and architectures. This gap between laboratory-scale demonstrations and industrial implementation remains one of the main limitations of the current literature and should be addressed through more systematic pilot-scale and industrial validation studies.

Overall, the future of paper-based packaging lies in the development of integrated performance–circularity design frameworks. Instead of optimising individual properties in isolation, next-generation systems should be designed through multi-criteria approaches that simultaneously consider barrier properties, mechanical behaviour, converting compatibility, safety, regulatory compliance, recyclability, compostability, and industrial feasibility. In this perspective, the interpretation of paper-based packaging as an engineered architecture—comprising substrate, coating layers, interfaces, and end-of-life pathways—provides a coherent framework for guiding future research and supporting the rational development of sustainable, high-performance packaging systems.

## 8. Conclusions

This review has examined the evolution of paper-based packaging from conventional cellulosic substrates to functionalised and hybrid architectures, highlighting how performance is no longer determined by material properties alone but by the combined contribution of coatings, interfaces, and structural organisation. Within this framework, paper-based packaging is interpreted as an engineered system in which barrier behaviour, mechanical response, converting compatibility, and end-of-life pathways are intrinsically interconnected.

The analysis shows that coating technologies, nanostructured systems, and multilayer architectures enable substantial improvements in barrier and functional performance but also introduce increasing structural complexity and associated constraints in recyclability and fiber recovery. As a result, the development of high-performance paper-based packaging systems is governed by a fundamental balance between performance enhancement and circularity requirements.

By integrating compositional, structural, and functional perspectives, the review provides a unified interpretative framework that links material selection, microstructural organisation, and architectural design to the main performance domains relevant to packaging applications. This system-level approach allows for the identification of recurring design patterns and trade-offs, offering a consistent basis for comparing different coating and multilayer strategies.

Overall, the work highlights that sustainable paper-based packaging requires the rational design of integrated architectures in which functionality and end-of-life compatibility are considered simultaneously.

## Figures and Tables

**Table 1:** **Classification of coating systems for paper-based packaging, integrating material composition and structural design criteria.** The table highlights typical material systems, structural organisation, dominant barrier mechanisms, functional roles, and main limitations. Monolayer systems refer to configurations with a single functional layer applied to the paper substrate, whereas multilayer systems involve two or more functional layers.

Coating System Class	Typical Material Systems	Structure	Dominant Mechanism	Key Function	Processing Compatibility	Main Limitations
Polysaccharide-based systems	Cellulose, CNF/CNC, starch, chitosan, alginate	Single functional layer (dense or modified film)	Barrier governed by dense hydrogen-bonded networks and reduced free volume	High oxygen barrier under dry conditions; film formation; biodegradability	Water-based coating; casting; rod/bar coating	High moisture sensitivity; poor water vapour barrier; swelling
Lipid-based systems	Waxes (beeswax, carnauba), fatty acids, lipids, shellac	Single functional layer; discontinuous or semi-continuous hydrophobic films	Surface-controlled barrier; reduced wettability and capillary penetration	Water resistance; grease barrier; surface protection	Emulsion coating; dispersion systems; thermal treatment	Film discontinuity; defects; limited gas barrier; poor mechanical strength
Protein-based systems	Proteins (gelatin, whey, soy, caseinate)	Single functional layer; continuous cohesive films	Diffusion-controlled barrier through dense polymer networks	Excellent oxygen barrier under dry conditions; film-forming ability; good mechanical performance	Solution coating; casting; drying	Strong moisture sensitivity; plasticisation; reduced barrier under humid conditions
Synthetic and bio-polymer coatings	PVOH, PLA, PBAT, PHBV, PE, starch blends, bio-based polyesters	Continuous polymer films or composite layers	Barrier behaviour governed by polymer chain packing, crystallinity, and film continuity	Balanced barrier performance; mechanical reinforcement; improved flexibility	Extrusion coating; lamination; solution coating	Sensitivity to humidity (e.g., PVOH); limited recyclability; fossil-based components in some systems
Hybrid and nanostructured systems	CNF + nanoclays, lignin nanoparticles, mineral fillers, nanocomposites	Nanostructured or reinforced coatings	Barrier enhancement associated with tortuous diffusion pathways and nanofiller dispersion	Enhanced gas barrier; mechanical reinforcement; multifunctionality	Aqueous processing; high-shear mixing; coating formulations	Dispersion challenges; scalability; potential migration issues
Multilayer and extrusion-coated systems	Paper combined with two or more functional layers, including polymeric layers (e.g., PE, PLA, PBAT), aluminium layers, and laminates	Two or more functional layers (multilayer architectures)	Barrier and mechanical performance governed by layer combination, interfacial adhesion, and structural integration	High barrier performance; mechanical robustness; tailored functionality	Extrusion coating; lamination; co-extrusion	Limited recyclability; difficult layer separation; incompatibility with fiber recovery processes; interfacial complexity; risk of barrier loss after converting or mechanical deformation

Note: Due to the heterogeneity of materials, processing conditions, and testing protocols across the cited studies, the reported properties and mechanisms are expressed in qualitative or semi-quantitative terms. The descriptors used in this table are intended to highlight relative trends and dominant behaviours rather than to provide directly comparable numerical values.

**Table 2:** **Cellulose-based mono-coating systems for paper and paperboard.** The reference condition is specified within the composition field because the selected studies do not adopt a uniform control substrate.

Coating System	Composition	Processing	WVTR/Water Uptake	Oxygen Barrier (Oxygen Permeability OP/OTR/Proxy)	Mechanical/Converting Indicators	Key Functional Traits	Main Limitation
Model/intrinsic systems (solution-cast/ideal films)							
Nanocellulose films—[38]	CNF + plasticiser + crosslinker (reference: corresponding baseline film formulation within the study)	Solution casting/multilayer films	water vapour permeability (WVP) ↓ ~60% (plasticised systems; dependent on formulation)	Very low OP (~0.7–2 mL·µm·m^−2^·day^−1^·kPa^−1^)	TS ↑ (~12 MPa); tunable stiffness and toughness	Dense hydrogen-bonded network; low free volume	Strong humidity sensitivity; ideal films, not representative of coated paper
Cellulose-based coatings on paper (real systems; study-specific paper/paperboard references)							
Cellulose-based coating—[39]	CNC/TOCN/CMC systems (reference: uncoated paperboard used in the study)	Doctor-blade coating	Not directly reported; grease/air barrier dominant	TOCN: ~10^−11^–10^−10^ cm^3^·m/m^2^·day·Pa (low RH)	Performance depends on coating continuity and coat weight	Continuous film formation; CMC improves uniformity	Strong degradation at high RH; defect-sensitive
Cellulose nanofibril coating—[40]	CNF (1.5–3 wt%), single vs. double layer (reference: uncoated base paper; internal comparison between coating layers)	Bar coating (layer-controlled)	Cobb ↓ ~75 → 58 g/m^2^ (~20–25%)	Air resistance ↑ ~90 → 210–420 s	Tensile index ↑ (~27 → 35 Nm/g); surface strength ↑; roughness ↓	Barrier controlled by layer number and coating continuity	Single-layer coatings insufficient; incomplete surface sealing
Nanofibrillated cellulose coating—[41]	CNF (0–0.4 wt%) (reference: uncoated base paper of the same study)	Meyer rod coating	water retention value (WRV) ↓ (~40%); Cobb ↓ (~27.5 → 24)	Air resistance ↑ (~1400 → 2050 s)	Slight tensile increase (~8.3 → 8.5 kN/m)	Pore filling and densification; optimal at intermediate loading	No direct OTR; performance depends on dispersion and loading
CNF/CMC coating—[42]	rCNF, gCNF ± CMC (reference: uncoated paper and formulation variants within the study)	Rod coating (1.6–7.8 g/m^2^)	WVTR ↓ ~490 → 370–390 (g/m^2^·day) (gCNF); limited for rCNF	Air resistance ↑ up to ~4700–5800 s	Roughness ↓; aqueous resistance ↑; grease up to Kit 6	Formulation-driven performance; CMC improves dispersion	WVTR improvement limited; strong formulation dependence
Microfibrillated cellulose coating—[43]	MFC multilayer coating (×1–×10) (reference: uncoated base paper; internal comparison across coating cycles)	Repeated coating	Cobb ↑ (~43 → 94–114 g/m^2^)	OP ~12–14 cm^3^/m^2^·day; no improvement	Bending stiffness and compressive strength ↑	Structural reinforcement without effective barrier formation	No barrier improvement; poor surface sealing

**Table 3:** **Starch-based mono-coating systems for paper and paperboard.** All systems are applied to paper substrates. Barrier properties are reported when directly measured (e.g., WVTR), while functional indicators (water absorption, oil resistance, mechanical properties) are included when barrier performance is inferred indirectly from structural effects.

Coating System	Composition	Processing	WVTR/Water Uptake	Oxygen barrier (OP/OTR/Proxy)	Mechanical/Converting Indicators	Key Functional Traits	Main Limitation
Starch-based coatings on paper (real systems)							
Starch coating—[45]	C-OSA, P-OSA, C-HP, P-HP (reference: uncoated paper used in the study)	Coating on paper (20 wt% solids)	WVTR ≈ 200–250 g·m^−2^·day^−1^ (starch-rich systems; high moisture permeability)	Not directly reported; limited barrier in humid conditions	Limited reinforcement; brittle behaviour at high starch content	Film-forming ability; direct application on paper	Very poor moisture barrier; strong hydrophilicity
Starch– poly(vinyl alcohol) (PVOH) coating—[45]	Starch:PVOH blends (≥40–60 wt% PVOH) (reference: uncoated paper and composition series)	Coating on paper	Significantly reduces WVTR to ~2–10 g·m^−2^·day^−1^	Improved barrier due to dense phase-separated structure	Improved flexibility and film integrity	Composition-controlled barrier; surface enrichment of PVOH	Strong composition dependence; barrier loss at high starch fraction
Starch-based PEC (polyelectrolyte complex) coating—[46]	Cationic/anionic modified starch (SPECs) (reference: uncoated paperboard)	Dip coating (~10 g·m^−2^)	WVP ↓ ~40%; Cobb_60_ water ↓; Cobb_60_ oil ↓ (~1.85 g·m^−2^)	Not directly reported; barrier via dense and homogeneous film	Tensile strength ↑ (~+18%); modulus ↑ (~+21%); grease resistance up to Kit 12	Ionic crosslinked network; defect-free coating; improved surface sealing	Strong dependence on molecular weight and network formation
Modified starch hybrid coating —[47]	Starch + lignin derivatives + CNF (reference: uncoated substrate)	Coating/composite formulation	Reduced permeability vs. neat starch (WVTR not always reported)	Improved air barrier (via densification and tortuosity)	Tensile and burst indices ↑	Hybrid network (H-bonding + electrostatic + filler reinforcement)	Complex formulation; dispersion and scalability limitations
Starch coating (functional behaviour)—[50]	Starch (reference: uncoated paper)	Coating on paper	Water absorption ↓ ~105 → ~40 g·m^−2^	Not directly measured (barrier inferred from reduced porosity)	Tensile ↑ (~80 → 90 N/m); burst ↑ (~320 → 380 kPa); oil resistance ↑ (Kit 1 → 3)	Surface densification and pore sealing; improved functional performance	No direct WVTR/OTR data; indirect barrier evidence only
Modified starch coating—[49]	Sodium starch octenyl succinate (SSOS) or maltodextrin (MAL) + sorbitol + glycerol (reference: coated paper formulations within the study)	Bar coating (two-layer application, IR drying)	Not directly reported; focus on sealability	Not directly reported	Heat-seal performance: SIT ~112–120 °C; FTT ~125–147 °C	Plasticization-induced chain mobility; improved heat sealability; interaction with fibrous network	Performance dependent on plasticizer balance; barrier properties not directly quantified
Modified starch–chitosan coating—[48]	Cationic-acetylated glutinous rice starch + chitosan (2:1) + glycerol (reference: uncoated kraft paper)	Rod coating (9.6 g/m^2^) + drying	WVT ↓ ~2580 → 1051 g/m^2^·24 h (~−59%); hydrophilic behaviour remained	Not directly reported;	grease barrier dominant → Tensile strength ↓ (~29.5 → 22.9 MPa); elongation at break ↑ (~2.4 → 3.8%)	Dense continuous film; complete fiber coverage; grease resistance improved from Kit 0 to Kit 12	Water resistance still limited; WCA decreased (~121.7° → 94.0°), indicating residual hydrophilicity

**Table 4:** **Alginate-based coating systems for paper and paperboard.** The table distinguishes intrinsic film behaviour, coating performance on fibrous substrates, and advanced structure-controlled systems. All systems refer to single-layer or composite coatings applied directly to paper substrates; fully developed multilayer packaging laminates are not considered.

Coating System	Composition	Processing	WVTR/Water Uptake	Oxygen Barrier (OP/OTR/Proxy)	Mechanical/Converting Indicators	Key Functional Traits	Main Limitation
Model/intrinsic systems (solution-cast films)							
Alginate film —[51]	Sodium alginate (reference: neat film)	Solution casting	WVTR values typically on the order of 10^2^–10^3^ g·m^−2^·day^−1^	OTR ≈ 0.5 cm^3^·m^−2^·day^−1^	Limited mechanical resistance in humid conditions	Good oxygen barrier; continuous film formation	Very high moisture sensitivity; swelling-induced permeability
Alginate coatings on paper (real systems)							
Alginate coating—[52]	Sodium alginate ± CaCl_2_/OMMT (reference: uncoated paperboard)	Coating on paperboard	MC ≈ 3.5–4.1 g/100 g; WVP ≈ 3.7–3.9 ng·m/m^2^·s·Pa; water absorption ↓	Not directly reported; Not directly reported, inferred from dense coating/air barrier limited barrier in humid conditions	TS ≈ 33–35 MPa (vs. ~40 MPa uncoated); elongation ↑ (~4–4.7%)	Continuous film; ionic crosslinking improves cohesion; filler enhances structure	Limited moisture barrier; performance depends on crosslinking
Alginate coating—[53]	Sodium alginate (reference: uncoated PF/SF paper)	Coating on paper	WVTR ↓ ~680 → ~380 g·m^−2^·day^−1^; Cobb ↑ (~25 → ~54 g·m^−2^), indicating increased water uptake	Air permeability → 0; strong barrier to gases and volatiles	Oil resistance: Kit 0 → 12; migration ↓ ~90%	Dense coating; complete pore sealing; effective functional barrier	Limited water vapour barrier; increased water uptake
Alginate/ZnO composite coating—[54]	Alginate (high molecular weight HMW or low molecular weight LMW) + ZnO at 1/1, 1/0.5, 1/0.25 (reference: uncoated filter paper)	Dip-coating + bar coating + drying	COBB increased (higher water uptake); high water uptake remained	Air permeability reduced; no direct OTR/WVTR reported Not directly reported; inferred from dense coating/air barrier	Dry strength ↑ (~0.213 → 0.322–0.365 kN·m/g); wet strength ↓ markedly;	antibacterial activity against *S. aureus* and *E. coli* → Composite functional coating; pore filling; improved dry strength and antibacterial performance	Hydrophilic behaviour remained critical; wet strength strongly decreased after water exposure
Advanced/structure-controlled systems							
Alginate composite coating—[55].	Alginate + collagen fibers (CF/SA) ± PVB (reference: uncoated paper)	Composite/layered coating	WVTR ↓ ~980 → ~130 → ~50 g·m^−2^·day^−1^ in composite multilayer systems rather than simple alginate coatings	Barrier enhanced via pore reduction and tortuosity	Mechanical strength ↑ (~12 → ~25 MPa); improved surface stability	Multilayer/composite architecture; reduced pore size; tortuous path	Requires complex architecture; not a simple mono-coating

**Table 5:** **Chitosan-based coating systems for paper and paperboard**. The table summarises single-layer coating configurations applied directly to paper substrates, including neat, modified, crosslinked, and composite systems. Although some formulations involve multiple components or chemical modification, all systems act as a single functional coating layer and do not constitute fully developed multilayer packaging architectures.

Coating System	Composition (Incl. Reference)	Processing	WVTR/Water Uptake	Oxygen/Air Barrier	Mechanical/Converting Indicators	Key Functional Traits	Main Limitation
Chitosan coatings on paper (baseline systems)							
Chitosan coating—[59]	Chitosan (reference: uncoated paper)	Aqueous coating/drying	Not directly reported; limited moisture barrier	Very high OTR (limited improvement vs. paper); moderate aroma reduction (~30–40%)	Dependent on coating continuity and substrate interaction	Thin pore-sealing layer; substrate-dependent performance	High gas permeability; barrier dominated by paper structure
Chitosan coating—[60]	Chitosan (reference: uncoated cellulose paper)	Surface coating	Not directly reported	Reduced permeability (qualitative)	Tensile index ↑ (~65 → 90 Nm/g); TEA ↑ (~1270 → 1850 mJ/g); improved thermal stability	Fiber–fiber bonding enhancement; surface consolidation	Moisture sensitivity; limited WVTR performance
Active and modified chitosan systems							
Chitosan active coating—[61]	Chitosan + essential oils (reference: uncoated paperboard)	Emulsion coating/drying	WVTR ~235–330 g·m^−2^·day^−1^; Cobb ↓ (~16–47 g·m^−2^)	Air permeability strongly reduced (~0.01–0.02 μm·Pa^−1^·s^−1^) (as reported in original study)	Additional flexibility depending on formulation	Combined water, air and grease barrier; active functionality (anti-insect)	Moderate moisture barrier; formulation-dependent performance
Crosslinked systems							
Chitosan crosslinked coating—[62]	Chitosan + phytic acid (reference: uncoated paper)	Coating + chemical crosslinking	Water resistance time up to ~30 min (optimum crosslinking)	Not reported	Not reported	Excellent grease barrier (Kit up to 12); oil resistance up to ~24 h; tunable properties via crosslinking	Strong dependence on crosslinking degree; performance loss at excessive crosslinking
Composite/hybrid mono-coatings							
Chitosan composite coating—[64]	Chitosan + PVA (reference: uncoated paper)	Single-layer coating (bar coater + drying)	WVTR reduced from ~10.1 to ~0.75 g·m^−2^·day^−1^ (~−92%) (under controlled conditions)	OP reduced from ~18.5 to ~0.87 (~−95%) (under controlled conditions)	Tensile strength ↑ (~4.3 → ~6.9 kN/m)	Dense continuous coating; pore sealing; strong H-bond network; improved hydrophobicity	Dependence on composition; potential impact on biodegradability due to synthetic component (PVA)
Chitosan hybrid coating—[65]	Chitosan + cellulose components (reference: uncoated paper)	Composite coating	Improved moisture resistance (qualitative)	Barrier improvement via densification	Mechanical properties ↑	Hybrid network; improved structural stability	Complex formulation; dispersion control
Chemically modified/hydrophobised systems							
Modified chitosan coating—[68]	Chitosan grafted with epoxidized soybean oil (CHI-g-ESO; reference: uncoated and CHI-coated paper)	Chemical modification + coating	Cobb ↓ from ~36 to ~9–11 g/m^2^	Not directly reported	Improved durability and surface stability	Covalent crosslinked network; reduced permeability; improved hydrophobicity and grease resistance (Kit ~8–11)	Lack of standard WVTR/OTR data; performance not directly comparable
Experimental benchmark system							
Chitosan coating—[67]	Chitosan ± astaxanthin ± genipin (reference: uncoated Kraft paper)	Coating + natural crosslinking	WVP ↓ (~12.2 → ~6.8 × 10^−4^); WC ↓ (~4.6 → ~3.8%)	OP ↓ (~0.53 → ~0.43)	Tensile strength ↑ (relative increase; values reported in the study); bursting strength ↑	Dense crosslinked network; improved hydrophobicity (contact angle > 100°)	Still humidity-sensitive; formulation-dependent performance

**Table 6:** **Lipid-based mono-coating systems for paper and paperboard**. All systems refer to single-layer or structurally equivalent hydrophobic coatings applied directly to paper substrates. Barrier performance is reported when experimentally available, while functional indicators are included when barrier behaviour is inferred from surface and structural effects. The selected systems collectively represent all the main lipid-based coating typologies discussed in this section, including wax-based, fatty-acid-based, oil-based, chemically modified, and resin-based systems, spanning surface-controlled, structured, and film-forming behaviours.

Coating System	Composition (Incl. Reference)	Processing	WVTR/Water Uptake	Oxygen Barrier (OTR/Proxy)	Mechanical/Converting Indicators	Key Functional Traits	Main Limitation
WAX-BASED SYSTEMS (surface-controlled)							
Wax-based coating—[76]	Natural waxes (emulsion wax, soywax, biowax, beeswax; reference: uncoated paper)	Wax coating/emulsion coating	WVTR reduced from ~1286 to ~284 g·m^−2^·day^−1^ (depending on wax type); WVP reduced from ~874 to ~196 g·mm·m^−2^·day^−1^·kPa^−1^; oil absorption reduced from ~99% to ~29–40%	Not reported; limited gas barrier expected	Tensile strength ↑ (~31 → 39 MPa); Young’s modulus ↑ (~3.0 → 4.3 GPa); elongation at break ↓ (~6.3 → 3.1%)	Discontinuous hydrophobic layer; surface-controlled barrier; pore blocking and reduced wettability	Structural heterogeneity; defect formation; limited oxygen barrier; strong dependence on coating uniformity
Structured wax emulsion coating—[78]	Soybean wax emulsion stabilized by surfactant or Pickering system; best performance with Pickering emulsion on paper substrates	Rod coating of wax-in-water emulsions	Cobb60 reduced from ~125 to ~39 g·m^−2^; WVTR reduced by ~30%; stable water holdout with WCA ~94° to 85° over 5 min	Not directly reported; barrier improvement inferred from reduced water uptake and improved holdout	Better strength retention than surfactant system; improved fold resistance and coating integrity	Structured wax layer; improved homogeneity and barrier continuity; enhanced water resistance compared with conventional wax coatings	Performance still dependent on emulsion stability, substrate type, and coating microstructure
FATTY-ACID-BASED SYSTEMS (surface-dominated)							
Stearic-acid-based system—[79]	PCC/CaCO_3_ modified with stearic acid + polymer binder	Surface treatment/coating	Superhydrophobic surface; water uptake strongly reduced	Not reported	Not reported	Micro-rough surface; WCA ~150°; wetting suppression	No continuous film; limited gas barrier
OIL-BASED AND CHEMICALLY MODIFIED SYSTEMS (intermediate regime)							
Tung oil-based coating—[82]	Tung oil-based photopolymerizable coating formulation on paper	Coating followed by UV curing	Cobb reduced from ~108 to ~17 g·m^−2^; contact angle increased from ~71° to ~127°	Not directly reported; improved barrier inferred from reduced water uptake and improved hydrophobicity	Optical and mechanical properties largely preserved at short curing times	Bio-oil coating with improved continuity; effective hydrophobic layer; simple solvent-free curing route	Primarily optimized for water barrier; gas barrier performance not fully quantified
Silylated soybean oil coating —[83]	Silylated soybean oil obtained via VTMS grafting and moisture-curing siloxane crosslinking on Kraft paper (reference: uncoated Kraft paper grades 60 and 78)	Roll and gravure coating; curing at ~80 °C via moisture-activated condensation (Si–O–Si network formation)	WVTR reduced by ~48–53% (from ~1350 to ~580–700 g·m^−2^·day^−1^); Cobb reduced from ~40 to ~22–26 g·m^−2^ (~35–45%)	Not directly reported; gas barrier remains limited	Crosslinked network improves coating stability; Uniform coating with good adhesion; moisture resistance improved through siloxane network formation	Crosslinked hydrophobic network; improved cohesion and resistance to moisture compared to conventional lipid coatings	Requires chemical modification; processing complexity; limited gas barrier performance
Camelina oil-based coating—[80]	Maleic anhydride-grafted camelina oil (modified plant oil lipid system)	Solution coating (rod coating; double layer)	WVP reduced from 2017 ± 125 to 175 ± 64 and 121 ± 23 g mil/m^2^ day (about 91–94% reduction) (units as reported in source); strong reduction in water vapour permeability	Oxygen permeability measured only for coated samples (814 ± 124 and 1221 ± 0 cc mil/m^2^ day); barrier performance remained formulation/curing-dependent	Tensile strength decreased from 15.78 ± 0.29 to 11.24–11.35 MPa; tensile modulus decreased from 1411.54 ± 17.37 to 714.20–796.60 MPa; elongation at break increased from 1.6 ± 0.12% to 6.4–8.3%	Strong improvement in moisture barrier; increased flexibility; formation of a more continuous hydrophobic layer	Reduced strength and stiffness relative to uncoated paper; oxygen-barrier improvement versus uncoated substrate not directly demonstrable
Fatty-acid-derived ester emulsion coating—[81]	Soybean-oil-derived wax emulsions based on ethylene glycol and propylene glycol mono- and di-esters of stearic acid (EGMD/PGMD), also compounded with commercial polymer emulsions	Emulsion coating on paperboard; multiple drawdowns; drying temperature varied	5 min Cobb reduced to ~1.0 g·m^−2^ in optimized compound emulsions; <2 g·m^−2^ with three drawdowns and higher coat weight	Not directly reported; barrier mainly inferred from water and grease resistance	Rheology and coating stability strongly dependent on emulsion type and emulsifier; drying temperature markedly affects performance	Fatty-acid-derived ester system; strong pore blocking; excellent water and grease resistance when coating structure is optimized	Requires emulsion formulation and drying optimization; performance depends on drawdown number, coat weight, and polymer-emulsion combination
Castor-oil-based coating—[84]	Castor-oil-based resin coating chemically bonded to paper surface through thiol–ene click reaction (reference: uncoated paper)	Surface chemical modification/coating followed by curing	WVTR reduced by ~40.3%; water contact angle increased to ~114°	Not directly reported	Tensile strength increased to ~26.9 MPa; thermal decomposition temperature increased to ~410.5 °C	Continuous hydrophobic network; strong bonding to cellulose; improved water resistance, mechanical strength, and removability under alkaline conditions	Requires chemical modification and curing; gas barrier not quantified; recyclability depends on coating removal conditions
RESIN-BASED SYSTEMS (diffusion-controlled regime)							
Shellac coating—[85]	Shellac-coated bagasse paperboard at 24–60% concentration, 1–4 layers (reference: uncoated BP)	Bar coating; optimum at 2 layers and 40% shellac	WVP down to 1.3 × 10^−12^ kg·m·m^−2^·s^−1^·Pa^−1^; Cobb60 down to 0.004 g·m^−2^	OP down to 3.8 × 10^−14^ kg·m·m^−2^·s^−1^·Pa^−1^	No significant effect on mechanical properties; improved thermal resistance	Continuous film; diffusion-controlled barrier; high uniformity and low defect density	Strong dependence on coating thickness and layer number; potential brittleness; processing constraints

**Table 7:** **Protein-based mono-coating systems for paper and paperboard.** All systems refer to single-layer protein-based coatings applied directly to paper substrates. Barrier performance is reported when experimentally available, while functional indicators are included when barrier behaviour is inferred from structural and surface effects.

Coating System	Composition (Incl. Reference)	Processing	WVTR/Water Uptake	Oxygen Barrier (OTR/Proxy)	Mechanical/Converting Indicators	Key Functional Traits	Main Limitation
FILM-FORMING PROTEIN SYSTEMS							
Sodium caseinate coating—[87].	Sodium caseinate (NaCAS) + plasticizer (protein-based coating system)	Solution coating + drying (≈10 g/m^2^ coating weight)	WVP reduced from 0.550 ± 0.018 to 0.221 ± 0.015 g·mm/m^2^·day·kPa (≈60% reduction vs. uncoated paper)	Not directly reported; improved gas barrier inferred from dense protein network	Tensile strength increased (0.329 → 0.352 MPa); elongation at break increased (3.04 → 3.49%)	Dense cohesive protein film; pore filling; diffusion-controlled barrier; improved mechanical integrity	Strong sensitivity to moisture; barrier performance decreases under high-humidity conditions
Gelatin coating—[34] *	Gelatin + glycerol (with/without modified curcumin)	Casting coating on paper surface + drying (≈0.43 mL/cm^2^)	WVTR reduced from 0.11 to 0.05 g/h·m^2^ (~55% reduction vs. paper)	O_2_ permeability measurable only after coating; significant barrier improvement compared to uncoated paper	Young’s modulus ↓ (2663 → ~530–720 MPa); tensile strength ↓; elongation ↑ (2.67 → 6–8.6%)	Continuous gelatin film; pore coverage; diffusion-controlled barrier; improved flexibility; heat-sealability	Reduced stiffness and strength; hydrophilic nature → moisture sensitivity
Whey protein coating—[88] **	WPI + plasticizer (glycerol or sorbitol), applied on PET substrate (reference: uncoated PET film)	solution coating on corona-treated PET + drying/curing	2–3 g/m^2^·day (normalized to 100 μm)	<2 cm^3^/m^2^·day·bar (normalized to 100 μm), comparable to EVOH	high stiffness of the crosslinked protein layer; good flexibility only with plasticizer; excellent adhesion to PET substrate	dense crosslinked protein network; excellent gas barrier; high transparency; good substrate adhesion	humidity sensitivity; barrier depends on plasticizer content and environmental conditions
MODIFIED/CROSSLINKED PROTEIN SYSTEMS							
Protein coating with micro-particles—[89] ***	Sodium caseinate/whey protein Sodium caseinate or whey protein coating (with glycerol plasticizer, optional microparticles) on kraft paper (reference: uncoated kraft paper, UKP)	aqueous solution coating (brush application on both sides) + drying at 80 °C	significant reduction in water absorption compared to uncoated paper	not reported	tensile strength improved compared to uncoated samples; dependent on microparticle type; good coating continuity and adhesion	improved moisture and oil resistance; pore sealing; formation of continuous protein film; enhanced surface protection	high residual water sensitivity; performance strongly dependent on protein type (casein > whey)
HYBRID/COMPOSITE PROTEIN SYSTEMS							
Whey protein/PVA coating—[91]	Whey protein + polyvinyl alcohol + glycerol (reference: uncoated paperboard, blank sample)	aqueous coating (roll coater, single-side) + drying at 35 °C	strong reduction in water permeability (10.08 → 3.97 L/m^2^·min, ~60% decrease)	not reported	improved mechanical resistance (load: 36 → ~57 N; extension: 2.72 → 4.35 mm); enhanced flexibility	pore filling; formation of dense hydrogen-bonded network (PVA–WP–cellulose); improved water barrier and mechanical integrity	residual hydrophilicity of protein fraction; performance strongly dependent on PVA content (higher PVA required for optimal barrier)
SPI–wax coating—[90]	Soy protein isolate (SPI) blended with polyethylene wax (0–70%) in aqueous dispersion (reference: uncoated paperboard)	aqueous dispersion coating (frame applicator) + drying and conditioning	strong reduction in WVP (3.74 → 0.45 g·mm/h·kPa·m^2^, ~88% decrease at 50% wax); Cobb reduced from 30.9 to 23.5 g/m^2^ at 50% wax and to 10.8 g/m^2^ at 70% wax	not reported (protein-based systems expected to provide good O_2_ barrier)	tensile strength slightly reduced (~80 → 68–77 MPa); elongation increased (5.7 → 7.2%)	hybrid mechanism combining pore filling (protein network) and hydrophobic phase (wax); tunable wettability (contact angle up to ~120°)	phase separation and loss of coating homogeneity at high wax content (>50%), leading to barrier deterioration

* Although described as a “bilayer” system in the original study (paper + coating), it is classified here as a monolayer coating, as only one functional layer is present. ** Barrier values refer to the whey-protein coating layer, calculated from coated PET structures and normalized to a thickness of 100 μm; the reference substrate in the original study was uncoated PET. *** Single-layer protein coating applied on paper substrate (referred to as coated paper systems; control: uncoated kraft paper).

**Table 9:** **Representative hybrid and nanostructured coating systems for paper-based packaging, organised according to the dominant transport-control mechanism.** The table distinguishes between (i) hybrid systems with bulk-controlled transport, in which barrier performance is governed by the interaction between a continuous matrix and a dispersed phase through mechanisms such as tortuous diffusion pathways, interfacial densification, and reduced free volume, and (ii) surface- and pore-controlled systems, where performance is primarily determined by wettability modification and partial sealing of the porous fibrous structure. Unlike material-based classifications, this organisation highlights the role of microstructural architecture, filler morphology, and matrix–filler interactions in controlling mass transport. Quantitative barrier data are reported when available, while qualitative trends are included when derived from experimental observations. The table provides a unified framework for interpreting the diverse coating systems reported in the literature and supports the mechanistic discussion presented in Section 2.6.

Coating System [Reference]	Matrix–Filler System	Filler Morphology	Key Microstructural Mechanism	Barrier Effect	Additional Functionality	Main Limitation
DENSE NETWORK FORMATION						
CNF coatings (CNF, LCNF, CM-CNF, S-CNF, AKD-CNF)—[118]	Monophase nanofibrillar cellulose network	High-aspect-ratio cellulose nanofibrils	Dense nanofibrillar network formation with pore filling, reduced pore size, and diffusion-controlled transport	Strong improvement in air barrier (Gurley up to >10^5^ s/100 cc) and grease resistance (Kit up to 12); Cobb reduced by ~75% at ~10 g/m^2^; WVTR moderately reduced, with AKD-CNF reaching ~100 g/m^2^·day; oxygen barrier remains limited, with OTR generally high and strongly dependent on fibril diameter and coating continuity	Barrier tunable via fibril diameter, coat weight (~10 g/m^2^ threshold), and surface modification (e.g., AKD)	Hydrophilicity and incomplete coating continuity limit water vapor and oxygen barrier; performance strongly dependent on fibril diameter, coating uniformity and coat weight
HYBRID SYSTEMS (BULK-CONTROLLED TRANSPORT)						
CNF–nanoclay hybrid—[25]	Cellulose nanofibrils (CNF) matrix + nanoclay (bentonite)	High-aspect-ratio nanoplatelets (layered silicates)	Formation of dense nanofibrillar network combined with tortuous diffusion pathways induced by aligned nanoclay platelets; reduction in pore size and interfacial densification	Significant reduction in WVTR (down to ~4 g m^−2^ day^−1^ under optimal coating conditions) and strong decrease in OTR with increasing coating weight and nanoclay content. OTR reduced from unmeasurable values (base paper) to ~8800–88,600 cm^3^ m^−2^ day^−1^ depending on formulation; significant reduction compared to base paper, but still insufficient for high-barrier applications	Tensile strength increased from ~6.3 to ~8.9 kN/m depending on formulation and processing conditions; Improved tensile strength and adhesion; enhanced coating continuity and pore sealing promoted by corona treatment	Incomplete pore sealing and coating inhomogeneity limit oxygen barrier; performance strongly dependent on filler dispersion and loading (mechanical–barrier trade-off)
Lignin nanoparticle coating—[123]	Cationic starch matrix + lignin nanoparticles (LNPs)	Spherical lignin nanoparticles (~100 nm)	Interfacial densification and reduced free volume through strong hydrogen bonding between LNPs and starch matrix; formation of a dense and continuous coating layer with pore filling and surface densification	WVTR reduced from ~2560 to ~426 g m^−2^ day^−1^ (~6× reduction); Cobb reduced to ~37 g m^−2^; WCA increased up to ~118°; Kit 9	Fully bio-based system; improved mechanical integrity (tensile strength up to ~48.9 MPa); combined water, vapour and oil barrier improvement	Limited tortuosity contribution compared to platelet-type fillers; performance sensitive to dispersion quality and potential aggregation at high processing intensity aggregation at excessive shear times
PVA–bentonite coating on cellulose paper—[106]	PVA + bentonite on cellulose paper	Bentonite nanosheets	“Brick-and-mortar” structure with Al–O–C coordination, reduced porosity, and tortuous transport path	WVTR reduced from uncoated paper (~10^3^ g m^−2^ day^−1^) to 4.59 g m^−2^ day^−1^; OTR ~264.96 cm^3^ m^−2^ day^−1^ 0.1 MPa^−1^; Cobb reduced to 8.47 g m^−2^ (vs highly hydrophilic control)	tensile strength increased from 8.84 to 50.47 MPa; modulus from 0.51 to 3.21 GPa	Requires thermal treatment and relatively high filler loading; performance remains dependent on PVA matrix properties and processing conditions
Crosslinked PVA/nanoclay hydrogel coating—[105]	PVA + nanoclay (borax-crosslinked hydrogel)	Exfoliated nanoclay platelets	“Fiber–brick” architecture combining crosslinked PVA network and tortuous diffusion pathway	WVTR reduced from 1861 to 195 g m^−2^ day^−1^ at 38 °C/90% RH and from 512 to 2 g m^−2^ day^−1^ at 23 °C/50% RH (vs uncoated paper)	Kit 12; Cobb ~10 g m^−2^ (vs. high water absorption of control); initial WCA 108°, maintained >90° after 3 min	Requires nanoclay exfoliation and controlled crosslinking; increased formulation complexity and sensitivity to processing conditions
Sc–CL–CNF–AgNPs coating—[47]	Carbamate starch (Sc) matrix + calcium lignosulfonate (CL) + cellulose nanofibrils (CNF) + AgNPs	Nanofibrillar network (CNF) + dispersed nanoparticles (AgNPs)	Dense hydrogen-bonded and electrostatically assembled network filling paper microvoids and forming a continuous coating layer; reduced free volume and bulk-controlled diffusion	Air permeability reduced by ~90–92% (from ~184 to ~14–17 L m^−2^ s^−1^); contact angle increased from ~20° to ~90–96°; improved air barrier, hydrophobicity, and UV-light shielding properties	Mechanical reinforcement (tensile/tear +20–40%, burst +~60%); antibacterial activity against *E. coli* and *S. aureus*; UV shielding; extended food shelf life (up to ~7 days for cherry tomatoes)	Moisture sensitivity typical of polysaccharide-based systems; limited quantitative gas barrier data and potential concerns related to nanoparticle migration.
PLA/PMMA/CNC nanocomposite coating—[124]	PLA/PMMA polymer blend matrix + modified cellulose nanocrystals (m-CNC)	Dispersed rod-like nanocrystals (CNC, 1–5 wt%)	Bulk-controlled transport via matrix densification and tortuous diffusion pathways induced by well-dispersed nanofillers; reduced free volume and improved interfacial compatibility	Strong reduction in water vapor permeability (WVP) (from ~2.25 × 10^−6^ to ~0.49 × 10^−6^ g·m/Pa·s·m^2^) and corresponding decrease in WVTR (from ~12.1 to ~2.65 g·h^−1^·m^−2^ at 250 μm); improved oil barrier (complete resistance at high coating thickness); increased hydrophobicity (WCA up to ~80°)	Mechanical reinforcement (tensile strength up to ~48–49 MPa; bending resistance ~45–48 mN depending on thickness); tunable via CNC content and coating grammage	Strong dependence on coating thickness and filler dispersion; performance plateau or slight decrease at high CNC loading due to aggregation and reduced interfacial efficiency
HNT/PE nanocomposite film –[128] *	Polyethylene (PE) matrix + halloysite nanotubes (HNTs)	Tubular clay nanoparticles (halloysite nanotubes)	Tortuous diffusion pathways induced by tubular nanofillers; combined with ethylene adsorption within the hollow lumen of HNTs; barrier strongly dependent on filler dispersion quality	OTR reduced by ~22% and WVTR by ~32% at 1 wt% HNT; barrier performance deteriorated at higher loading due to aggregation-induced defects	Active ethylene scavenging leading to delayed ripening of bananas and tomatoes (firmness loss reduced from ~72% to ~16%); reduced weight loss of strawberries (up to ~53% reduction); and lower aerobic bacterial growth in chicken samples due to combined gas barrier effects	Strong dependence on filler loading and dispersion; aggregation at higher loading increases permeability despite improved active functionality
CS–CNC nanocomposite coating—[126]	Cationic starch (CS) matrix + cellulose nanocrystals (CNC/NCC)	Rod-like cellulose nanocrystals (length ~100–200 nm; width ~10–20 nm)	Network-controlled densification through hydrogen-bonded percolated nanostructure; pore filling and formation of a continuous coating layer with reduced surface porosity; performance governed by nanoparticle dispersion and interfacial interactions	Water absorption reduced from ~65.4 to ~33.3 g/m^2^ at 5 wt% NCC; air resistance increased from ~46 to ~721 s; oil permeability reduced from ~88% to ~44%; barrier enhancement maximized at 5 wt% NCC	Tensile strength increased from ~45 to ~54 Nm/g due to formation of a coherent reinforcing layer and strong interfacial interactions between NCC, cationic starch, and paper fibers; Reduced surface roughness (from ~79 to ~39 mL/min) and improved coating uniformity due to pore filling and formation of a smooth nanostructured layer	Agglomeration at higher CNC loading limits further improvement; starch matrix remains intrinsically moisture-sensitive
SURFACE- AND PORE-CONTROLLED SYSTEMS						
Ethyl cellulose + bio-CaCO_3_—[127]	Hydrophobic polymer + mineral filler	Plate-like bio-CaCO_3_ particles (2–5 µm diameter; ~0.5 µm thickness)	Surface-controlled hydrophobisation combined with pore sealing via EC film formation and CaCO_3_ particle deposition; performance governed by reduced porosity and increased surface hydrophobicity	Water absorption reduced from ~150 to ~9 g/m^2^ (FP) and from ~43 to ~7 g/m^2^ (PAP); WCA increased up to ~122–132° with SA-modified CaCO_3_; barrier improvement driven by combined hydrophobicity and pore blocking; Gas barrier not reported in the study	Hydrophobic surface Reduction in tensile strength compared to neat EC coating (e.g., 45 → 36–37 MPa in PAP); increased elongation retained; behaviour indicative of filler-induced discontinuities	Barrier performance governed by surface-controlled mechanisms rather than continuous film formation; particle-induced discontinuities limit mechanical integrity and coating uniformity

* Note: Included as Reference Bulk Nanocomposite System (Not Paper-Based Coating).

**Table 10:** **Design-oriented comparison of multilayer architectures in paper-based packaging.** The table summarises the main multilayer architecture types discussed in Section 3 in terms of structural logic, typical materials, barrier profile, end-of-life implications, and main limitations. The reported characteristics represent general design trends rather than fixed values associated with a single configuration. Detailed case studies supporting this comparison are reported in Table S1 (Supplementary Materials).

Architecture Type	Structural Logic	Typical Materials	Barrier Profile	End-of-Life Profile	Main Limitation
Bio-based multilayer coatings—functional multilayer systems	Distinct layers provide complementary functions, typically combining gas-barrier layers with moisture-protective or mechanically stabilising outer layers	Polysaccharides, proteins, bio-polyesters, lipids, hybrid bio-based layers; may include nanocellulose, nanoclays, or hybrid inorganic–organic interlayers	High oxygen barrier can be achieved under dry or controlled-humidity conditions; moisture barrier is moderate and strongly dependent on outer-layer design	Potentially recyclable, biodegradable, or compostable depending on formulation and substrate compatibility	Moisture sensitivity, structural complexity, and variable converting stability
Bio-based multilayer coatings—simple bilayer systems	Two-layer architectures with limited functional differentiation, generally based on surface sealing and partial hydrophobization	Polysaccharide/lipid, polysaccharide/polymer, or protein/polymer combinations	Limited to moderate barrier improvement; generally better liquid-water or moisture response than monolayers but lower performance than engineered multilayers	Generally favourable for biodegradability and paper compatibility	Limited robustness and weaker control of interfacial performance
Bio-based multilayer coatings—sequential or LbL systems	Barrier improvement is achieved through repeated deposition, progressive densification, and controlled thin-layer build-up	Chitosan, nanocellulose, polyelectrolytes, charged biopolymers, thin hybrid layers	Good gas barrier may be achieved through densification and tortuous transport paths; moisture barrier is usually limited	Potentially biodegradable, but large-scale implementation may be less straightforward	Process complexity and high sensitivity to environmental conditions
Polymer–paper multilayer and extrusion-coated systems	Paper substrate combined with continuous polymer films acting as structural and barrier layers	Paper with PLA, PBS, PBAT, PHB, PHBV, TPS-based blends, or related bio-polyesters	Generally good moisture and grease barrier; oxygen barrier ranges from moderate to high depending on polymer and layer configuration	Partially recyclable or compostable in selected cases, but often limited by multi-material structure	Interlayer incompatibility, limited recyclability, and brittleness in some systems
High-performance laminated systems (industrial benchmark)	Fully differentiated multilayer structures with separate structural, sealing, and barrier layers	Paper or paperboard combined with PE, EVOH, metallized layers, SiOx/AlOx coatings, or aluminum foil	Very high barrier performance; near-zero permeability in aluminum-based systems; excellent oxygen barrier in EVOH-based systems under controlled humidity	Generally poor to moderate recyclability due to structural complexity and difficult separation	Trade-off between maximum barrier performance and circularity

**Table 11:** **Functional capabilities and design trade-offs of paper-based packaging systems.** Qualitative performance levels are derived from trends observed in the literature discussed in Section 2 and Section 3, including representative experimental data. The reported values indicate typical functional behaviour under relevant conditions rather than directly comparable metrics. Oxygen barrier performance reflects behaviour under dry conditions unless otherwise specified, and may decrease significantly under high humidity for hydrophilic systems. Structural complexity increases from single-phase coatings to fully integrated multilayer architectures with distinct functional interfaces.

Strategy/System Type	Oxygen Barrier	Water Vapour Barrier	Grease Resistance	Mechanical Behaviour	Converting Compatibility	Circularity	Structural Complexity	Main Limitation
Uncoated paper	Low	Low	Low	High stiffness/low flexibility	Excellent	Excellent	Low	Intrinsically poor barrier performance
Polysaccharide-based coatings	High (dry; humidity-sensitive)	Low–Moderate	Moderate–High	Stiff, brittle	Moderate	High	Low	Strong moisture sensitivity
Protein-based coatings	High (dry; humidity-sensitive)	Low–Moderate	Moderate	Brittle, low flexibility	Moderate	High	Low	Moisture-induced plasticisation
Lipid-based coatings	Low	High	Very high	Low cohesion, weak structure	Good	High	Low	Limited gas barrier
Thermoplastic coatings (bio-based and synthetic)	Moderate–High (formulation-dependent)	Moderate–High (structure-dependent)	Moderate–High	Flexible/tunable	Good	Moderate	Low–Moderate	Trade-off with recyclability
Nanostructured/hybrid coatings	High (structure-dependent)	Moderate (humidity-sensitive)	High	Reinforced, variable	Moderate	Moderate	Moderate	Dispersion and structural continuity
Multilayer/laminated systems	Very high	Very high	High–Very high	Layer-dependent; interface-sensitive	Moderate	Low	High	Interfacial defects and limited recyclability

**Table 12:** **Structure–property relationships governing barrier performance in paper-based packaging systems.** The table compares the main material systems and design strategies in terms of dominant transport mechanisms and their effectiveness against different permeating agents, including gases, water vapour, grease, and light. Qualitative levels are derived from the trends discussed in Section 2 and Section 3.

System Type	Dominant Transport Mechanism	O_2_ Barrier	Moisture Barrier (WVTR)	Grease Resistance	Light Barrier	Key Structural Factor	Main Limitation
Uncoated paper substrate	Pore-controlled transport (open network diffusion)	Low	Low	Low–Moderate	Low	High porosity, interconnected fiber network	Uncontrolled permeability
Densified fiber network	Reduced pore transport	Moderate	Moderate	Moderate	Low	Reduced porosity, improved fiber packing	Transport pathways remain continuous
Bio-based coatings	Diffusion through hydrophilic film	Good (dry)	Moderate	Good	Low	Film continuity, hydrogen-bonded structure	Moisture sensitivity, swelling
Polymeric coatings	Diffusion through continuous polymer phase	Very good	Good	Very good	Moderate	Continuous film, low polarity	Defects and thickness dependence
Nanostructured coatings	Tortuous path diffusion (reduced diffusivity)	Excellent	Good	Good	Moderate	Filler alignment, dispersion quality	Sensitivity to defects, scalability
Bio-based multilayer systems	Sequential diffusion + partial defect decoupling	Very good	Good	Good	Moderate	Layer combination, partial redundancy	Interfacial compatibility
Polymeric multilayer systems	Multi-step diffusion + defect misalignment	Excellent	Very good	Very good	Good	Layer continuity, interfacial adhesion	Interfacial defects and adhesion variability
High-barrier multilayer systems (EVOH, Al, metallized)	Near-zero permeability/impermeable layer control	Excellent	Excellent	Excellent	Excellent	Dense layers, metallic barriers	Delamination risk, poor recyclability

Note: Qualitative levels are derived from trends reported across the literature rather than from a single dataset.

**Table 13:** **Qualitative comparison of sealability, adhesion, and layer stability in paper-based packaging systems.** The reported levels reflect typical behaviour observed across the main material systems and design strategies discussed in Section 2 and Section 3. Sealability generally increases with the introduction of thermoplastic phases, whereas adhesion and layer stability depend more strongly on interfacial compatibility, defect formation, and architectural design.

System Type	Sealability	Adhesion	Layer Stability
Uncoated paper substrate	Low	Low	Low
Densified fiber network	Low	Moderate	Moderate
Bio-based coatings	Moderate	Moderate	Moderate
Polymeric coatings	High	Good	Very good
Reinforced/nanostructured coatings	Good	Moderate	Good
Multilayer systems	Very good	Good (system-dependent)	Good (interface-dependent)
Laminated systems	Excellent	Very good	Very good

Note: The reported levels are derived from qualitative trends across the literature rather than from a single dataset.

**Table 14:** **Qualitative evolution of mechanical performance and converting-related parameters across paper-based material architectures.**

Material Architecture	Mechanical Performance	Printability	Folding & Creasing Resistance	Thermoformability	Process Stability
Uncoated paper	Moderate	Low	Very good	Low	Low
Densified fiber network	Good	Moderate	Good	Low	Moderate
Bio-based coatings	Moderate	Moderate	Moderate	Moderate	Moderate
Polymeric coatings	Good	Good	Good	Good	Good
Nanostructured coatings	Very good	Good	Moderate	Good	Moderate
Multilayer systems	Very good	Very good	Moderate	Very good	Very good
Laminated systems	Excellent	Excellent	Moderate	Excellent	Excellent

Note: The reported levels are based on qualitative trends observed across the literature and on the hierarchical interpretation discussed in Section 4.2.1 and Section 4.2.4. They are intended to summarise relative tendencies rather than provide direct quantitative rankings, since the systems differ in substrate type, coating formulation, processing route, structural configuration, and testing protocol.

## Data Availability

No new data were created or analyzed in this study. Data sharing is not applicable to this article.

## Supplementary Materials

The following supporting information can be downloaded at: https://www.mdpi.com/article/10.3390/ma19132801/s1, Table S1. Representative multilayer, extrusion-coated, and laminated systems for paper-based packaging, organised by architecture type. References [25,105,114,131,132,133,134,135,136,137,138,139,140,141,142,143] are cited in Supplementary Materials.

## Abbreviations

The following abbreviations are used in this manuscript:

AKD	Alkyl Succinic Anhydride
AlOx	Aluminum Oxides
ASTM	ASTM International
BA	Boric Acid
BfR	German Federal Institute for Risk Assessment (*Bundesinstitut für Risikobewertung*)
CB	Cocoa Butter
CEN	European Committee for Standardization (*Comité Européen de Normalisation*)
CEPI	Confederation of European Paper Industries
Cht	Chitosan
CMC	Carboxymethyl Cellulose
CNC	Cellulose Nanocrystals
CNF	Cellulose Nanofibrils
CPBAT	Crystallizable Poly(butylene adipate-co-terephthalate)
CPLA	Crystallizable Polylactic Acid
EAA	Poly(ethylene-co-acrylic acid)
ECH	Epichlorohydrin
EDQM	European Directorate for the Quality of Medicines & HealthCare
EFSA	European Food Safety Authority
EGMD	Ethylene Glycol Mono- and Di-esters of Stearic Acid
ESO	Epoxidized Soybean Oil
EU	European Union
EuPIA	European Printing Ink Association
EVA	Poly(ethylene-co-vinyl acetate)
EVOH	Ethylene Vinyl Alcohol Copolymer
GAB	Guggenheim–Andersen–de Boer
GMP	Good Manufacturing Practice
HDPE	High-Density Polyethylene
HCl	Hydrochloric Acid
HM-EHEC	Hydrophobically Modified Ethyl Hydroxyethyl Cellulose
LDPE	Low-Density Polyethylene
MAL	Maltodextrin
MeNC	Methylcellulose
MFC	Microfibrillated Cellulose
MFFT	Minimum Film Formation Temperature
MOAH	Mineral Oil Aromatic Hydrocarbons
MOSH	Mineral Oil Saturated Hydrocarbons
NaCAS	Sodium Caseinate
NIAS	Non-Intentionally Added Substances
OML	Overall Migration Limit
OP	Oxygen Permeability
OTR	Oxygen Transmission Rate
PAP/PAPs	Paper/Paper Substrates
PBAT	Poly(butylene adipate-co-terephthalate)
PBS	Poly(butylene succinate)
PCC	Precipitated Calcium Carbonate
PE	Polyethylene
PEC	Polyelectrolyte Complex
PEG	Polyethylene Glycol
PET	Polyethylene Terephthalate
PFAS	Per- and Polyfluoroalkyl Substances
PFJ	Potato Fruit Juice
PHA	Polyhydroxyalkanoates
PHB	Poly(3-hydroxybiturate)
PHBV	Poly(3-hydroxybutyrate-co-3-hydroxyvalerate)
PLA	Polylactic Acid
PLAX	Bio-based PLA Copolymer Dispersions
PMMA	Poly(methyl methacrylate)
POA	Acid-Modified Polyvinyl Alcohol
PP	Polypropylene
PPWR	Packaging and Packaging Waste Regulation
PVB	Polyvinyl Butyral
PVOH/PVA	Poly(vinyl alcohol)
RH	Relative Humidity
SiOx	Silicon Oxides
SML	Specific Migration Limit
SPI	Soy Protein Isolate
SSOS	Sodium Starch Octenyl Succinate
TEC	Triethyl Citrate
Tg	Glass Transition Temperature
TOCN	Tempo-Oxidized Cellulose Nanofibers
TPS	Thermoplastic Starch
UKP	Uncoated Kraft Paper
VOCs	Volatile Organic Compounds
VTMS	Vinyltrimethoxysilane
WVP	Water Vapour Permeability
WVTR/WVT	Water Vapour Transmission Rate
WPI	Whey Protein Isolate
WRV	Water Retention Value
ZnO	Zinc Oxide
