# Supplementary material for: Paper and Cardboard Packaging: From Cellulosic Substrates to Functional and Hybrid Architectures
Source: Materials (Basel). 2026 Jul 1;19(13):2801. doi: 10.3390/ma19132801 (PMC13362800; doi:10.3390/ma19132801)
Supplement: Supplementary file 1 [file materials-19-02801-s001.zip › materials-4341252-supplementary.pdf]

Supplementary Materials

Table S1. Representative multilayer, extrusion-coated, and laminated systems for paper-based packaging, organised by architecture type.

The table reports representative case studies grouped according to structural configuration, highlighting composition, processing routes, barrier behaviour, and key limitations in real systems.

Group 1 – Bio-Based Multilayer Coating Systems

Coating System	Composition	Processing	WVTR	OTR	Mechanical / Converting Behaviour	Functional Mechanism	End-of-Life / Compliance	Main Limitation
**Group 1A – Functional multilayer systems**<br>**Functionally stratified multilayer systems, where distinct layers provide complementary barrier and mechanical functions.**								
TPS-based multilayer system –[25]	Paper / TPS (~12 µm) / PLA–PBAT–PHBV–PVOH (~12 µm)	Solution coating (TPS) + second polymer coating (multilayer assembly)	WVTR reduced to 1.79 g/m²·day (PHBV multilayer)	O_2 permeance reduced to ~0.024 m³/(Bar·m²·s)	Improved tensile strength (up to ~16 MPa depending on polymer), enhanced flexibility (PBAT), and partial wet strength improvement	Multilayer diffusion control with TPS pore filling and polarity contrast between layers	Compostability dependent on polymer type (TPS/PHBV vs PLA/PBAT)	moisture sensitivity (TPS core layer); structural complexity
PFJ–PHB multilayer coating –[131]	Potato fruit juice (PFJ) interlayer + PHB outer layer(s) on paperboard; two-layer (PFJ/PHB) and three-layer (PHB/PFJ/PHB) architectures	Rod coating of PFJ + extrusion coating of PHB	Strongly reduced; sWVTR down to ~5 g·mm/(m²·day) for the best two-layer systems and ~16–21 g·mm/(m²·day) for three-layer systems	Moderate; best three-layer systems reached OP ≈ 262–367 cm³·mm/(m²·day·atm), whereas two-layer systems remained higher	Moderate peel strength (up to ~28.6 N/m in two-layer systems); three-layer systems showed better creasing and grease barrier performance, but heat sealing remained poor	Multilayer diffusion control, with PFJ providing oxygen barrier and improved interfacial adhesion, and PHB acting as moisture-protective outer layer	Bio-based and potentially biodegradable multilayer concept (depending on PHB behaviour and system integration)	Pinholes/cracks in PHB-coated layers limited oxygen barrier; two-layer systems showed poorer creasing performance and poor heat-sealing behaviour
PLAX/bioORMOCER®/PLAX multilayer coating –[132]	PLA-based dispersion + hybrid inorganic–organic lacquer (bioORMOCER®)	Industrial rod coating + reverse gravure coating	WVTR: 12–15 g/m²·day	OTR: 2.3–29 cc/m²·day·bar depending on substrate and testing conditions (0–50% RH)	Improved barrier with low coat weight; scalable processing	Multilayer diffusion control with complementary layer functions: bioORMOCER® providing oxygen barrier and PLAX layers acting as protective and moisture-barrier components	Bio-based multilayer system with potential recyclability/biodegradability; end-of-life performance not fully validated	Enhanced barrier performance at low coat weight with demonstrated scalability at pilot and industrial level
Multilayer bio-based coating –[133]	Paper/PVA/D-SiO_2/CS–CW/PA	Layer-by-layer coating (rod coating	WVTR ≈ 16.2 g/m²·day	OTR ≈ 0.51 cm³/m²·d	Improved tensile strength (~48	Hierarchical multilayer barrier: pore	Partially bio-based multilayer system; presence of	Multistep sequential coating

Coating System	Composition	Processing	WVTR	OTR	Mechanical / Converting Behaviour	Functional Mechanism	End-of-Life / Compliance	Main Limitation
	sequential coating (PVA base layer; hydrophobic silica interlayer; chitosan–carnauba wax emulsion; polyacrylate topcoat)	+ spray deposition + drying cycles)	(≈100× reduction vs paper)	ay·0.1 MPa (corresponding to ~10^5 reduction vs uncoated paper)	→ 63 MPa) and elongation (~7.5 → 10.5%); enhanced thermal stability (T_onset ↑ to ~350°C)	sealing (PVA) + tortuous path (SiO_2) + hydrophobic domains (wax) + defect sealing/top protection (PA)	polyacrylate limits full biodegradability	process; multilayer architecture may complicate recycling
PLA/CNC–PVA/PLA multilayer laminate –[105]	PLA outer layers + shear-oriented CNC/PVA inner coating (~5–6 μm)	Blade coating (CNC/PVA) + hot roll lamination (PLA layers)	WVTR ≈ 5 g/m²·day (dry-cup; strong reduction vs neat PLA ~45 g/m²·day)	OTR ≈ 10–45 cm³/m²·day (50–70% RH; ~1–2 orders of magnitude reduction vs PLA)	Good tensile performance with retained ductility; moderate interlayer adhesion (peel ≈ 24 N/m); slight delamination under strain	Functional multilayer barrier: highly aligned CNC network (low free volume) + PVA matrix (oxygen barrier) + outer PLA layers (moisture protection and mechanical support)	Fully bio-based multilayer system (PLA + CNC/PVA); potentially biodegradable depending on conditions	Humidity sensitivity of CNC/PVA layer; interfacial adhesion limits; barrier performance decreases at high RH
**Group 1B – Simple multilayer/bilayer systems**<br>**Simple bilayer systems, in which multiple layers are present but functional separation is limited.**								
CNC/Cht bi-layer coating –[134]	CNC (11 wt%) base layer (22–33 g/m²) + chitosan top layer (3–4 g/m²)	Slot-die coating + NIR drying	WVTR ≈ 68–71 g/m²·day (bi-layer)	OTR ≈ 478–257 cm³/m²·day (strong reduction vs uncoated)	Improved surface strength (z-direction tensile); formation of compact and homogeneous coating layers	Dense hydrogen-bonded CNC network (bulk diffusion barrier) + pore sealing and electrostatic interaction by Cht layer	Bio-based, recyclable, compatible with paper recycling	Moisture sensitivity of polysaccharides; performance depends on coating weight and layer uniformity
CNF/PLA–CNF/CB multilayer coating –[135]	CNF-stabilized Pickering emulsions: PLA layer + cocoa butter (CB) layer (sequential)	Rod coating (two-step deposition + drying + pressing)	WVTR reduced from ~29.2 to ~7.06 g/m²·h (Kraft); ~30.6 to ~14.37 g/m²·h (Bond)	Improved moisture barrier; gas barrier not reported (expected moderate due to film continuity)	Uniform coating, reduced porosity, improved surface hydrophobicity (contact angle ~87°)	Tortuous path (CNF) + continuous polymer film (PLA) + hydrophobic lipid barrier (CB)	Bio-based, waterborne, recyclable-compatible system	Layer adhesion and moisture sensitivity of polysaccharide phase
PVOH–Zein bilayer coating –[136]	Polyvinyl alcohol (bottom	Rod coating (two-step	WVTR reduced from ~1608	Gas barrier not	Increased tensile strength (MD	Film continuity + pore sealing	Bio-based, recyclable, food-contact compatible system	Moisture sensitivity of PVOH

Coating System	Composition	Processing	WVTR	OTR	Mechanical / Converting Behaviour	Functional Mechanism	End-of-Life / Compliance	Main Limitation
	layer, ~5 wt%) + zein protein (top layer, 12.5–20 wt%) sequential coating	deposition + drying)	to ~175 g/m²·day (~90% reduction)	directly measured; expected improvement due to dense PVOH film and pore sealing	up to ~59 MPa equivalent), burst strength (~85 lb/in²), and ring crush resistance; reduced friction angle (~20.7→11.2°); improved surface smoothness and cohesion	(PVOH) + hydrophobic surface layer (zein) → enhanced structural integrity and barrier effect		layer; performance dependent on coating uniformity and humidity
**Group 1C – Sequential / LbL coatings**<br>**Sequential or layer-by-layer coatings, where performance arises from progressive densification and film formation.**								
MeNC/MFC/HM-EHEC multilayer –[137]	MeNC + MFC + HM-EHEC (layer-by-layer cellulose-based system)	Spray coating + rod coating (multilayer deposition)	Moderate–high; no effective moisture barrier; strongly RH-dependent	OTR ~200–400 cc/m²·day at 50% RH, increasing up to ~1000–1200 at 80% RH (estimated from Fig. 8)	Increased roughness with MFC; good coating coverage; pore closure at ≥10 g/m²	Pore sealing + dense hydrogen-bonded network → near-zero air permeability; limited hydrophobic contribution from HM-EHEC	Fully bio-based; recyclable and biodegradable potential	Strong humidity sensitivity; poor water vapour barrier; performance degradation at high RH
Chitosan multilayer coating –[138]	Chitosan (1 wt% in acetic acid), 1–5 sequential coating layers on cellulose paper	Rod coating (repeated layer-by-layer deposition + thermal drying at 100 °C)	Moisture barrier not quantified; no WVTR reported	Not reported	Improved tensile strength (up to ~44.9 MPa dry; +156% in wet conditions), increased strain at break (~2.3→3.5%), enhanced wet and flexibility	Pore filling + hydrogen bonding with cellulose → progressive film formation (≥3 layers) → densification and barrier improvement	Fully bio-based, biodegradable, suitable for food-contact (migration <10 mg/dm² under specific conditions)	Limited intrinsic moisture resistance; performance dependent on humidity and number of layers

Group 2 – Polymer–Paper Multilayer and Extrusion-Coated Systems

Coating System	Composition	Processing	WVTR	OTR	Mechanical / Converting Behaviour	Functional Mechanism	End-of-Life / Compliance	Main Limitation
Bio-based extrusion coating –[114]	Paperboard + single-layer extrusion coatings (Bio-PE, PBS FX, PBS FZ)	Extrusion coating (pilot-scale) + plasma treatment	WVTR ≈ 3–4 g/m²·day (Bio-PE); PBS grades >50 g/m²·day (above measurement range)	Poor-to-moderate O_2 barrier; best ≈590 cm³/m²·day (PBS FZ)	Comparable mechanical properties; surface microcracks observed after creasing but limited impact on barrier	Continuous thermoplastic film → pore sealing + moisture barrier	Bio-PE: recyclable, non-biodegradable; PBS: compostable (home/industrial depending on grade)	Limited oxygen barrier; adhesion issues (PBS); performance variability due to coating uniformity

Coating System	Composition	Processing	WVTR	OTR	Mechanical / Converting Behaviour	Functional Mechanism	End-of-Life / Compliance	Main Limitation
PHBV/paper/PHBV multilayer –[139]	PHBV films (~12 µm each side) + paper core (~290 µm)	Heat sealing (140 °C, ~8 s, 20 bar)	Reduced vs paper; WVTR ~4.5 ± 0.8 $\times 10^{-10}$ kg/m²·Pa·s	Improved; O_2 permeance ~3.4 ± 0.8 $\times 10^{-15}$ m³/m²·Pa·s	Improved mechanical performance vs paper; increased stiffness and strength; moderate ductility; good interlayer adhesion	Continuous hydrophobic polymer layers + pore sealing + multilayer diffusion control	Biodegradable and compostable (PHBV-based); slight delay in paper disintegration	Intrinsic brittleness of PHBV; cracking/pinhole defects may affect barrier; limited sealability
PBAT/paper/PBAT multilayer –[140]	PBAT films (~50 µm each side) + recycled paperboard core (~120 µm)	Hot pressing (~150 °C, ~10 bar; lamination without adhesives)	Very low; no water absorption (Cobb ≈ 0); strong reduction of WVTR	Low oxygen permeability; strong reduction vs uncoated paper (qualitative)	Increased stiffness and mechanical resistance (bending ≈80→131 mN MD; bursting ≈250→564 kPa); improved surface smoothness and adhesion; slight decrease in tensile strength and elongation; excellent grease resistance (kit value 12)	Continuous hydrophobic polymer layers + pore sealing + dense film formation → multilayer diffusion control	Biodegradable polymer (PBAT) combined with recyclable paperboard; potential for circular packaging	Loss of barrier at elevated temperature; reduced tensile performance; multilayer structure may limit recyclability

Group 3 – Laminated and High -Barrier Systems

Coating System	Composition	Processing	WVTR	OTR	Mechanical / Converting Behaviour	Functional Mechanism	End-of-Life / Compliance	Main Limitation
Aluminum-based multilayer laminate –[141] [142].	Paperboard (~75%) + LDPE (~20%, multilayer) + aluminum foil (~5%)	Lamination + thermo-mechanical assembly (multilayer conversion with heat and pressure)	Near-zero (excellent moisture barrier due to polymer + Al layers)	Near-zero oxygen permeability; total barrier to gases, light and volatiles	High structural integrity; excellent stiffness from paperboard; good sealability via polyethylene; mechanical response governed by layered composite behaviour and interfacial adhesion	Metallic barrier (Al foil) + continuous polymer layers + functional layering → complete diffusion blocking and structural synergy	Poor recyclability; multi-material structure difficult to separate; partial recovery mainly of paper fraction (~75%)	High structural complexity; difficult end-of-life management; interlayer delamination issues
Cellulose tray with PE/EVOH/PE multilayer coating –[143]	cellulose substrate + PE/EVOH/PE coating + multilayer sealing film (PE/EVOH)	Industrial tray forming + multilayer coating (commercial) + MAP heat sealing	Not reported; moisture sensitivity and barrier instability under	OTR ≈ 0.1 mL O_2/package/day (package-level)	High initial compression strength (~115 N) with significant reduction during storage	Multilayer barrier: PE (moisture/sealing) + EVOH (oxygen barrier) enabling MAP gas retention, but performance	Hybrid structure; potentially recyclable through separation of	Mechanical instability under high humidity and fatty food contact; structural

Coating System	Composition	Processing	WVTR	OTR	Mechanical / Converting Behaviour	Functional Mechanism	End-of-Life / Compliance	Main Limitation
			humid and fatty conditions		(down to ~72 N), strongly dependent on food type, humidity and fat content	affected by moisture-induced degradation and interfacial instability	cellulose and plastic layers	degradation during storage
